# Substituent-controlled visible light photoisomerization and cyclodimerization of styryl pyrimidinones

**DOI:** 10.1039/d6ra06890a

**Published:** 2026-10-08

**Authors:** Mikhail V. Polynski, Zaruhi A. Hovasyan, Arthur A. Harutyunyan, Astghik A. Shahkhatuni, Aleksan G. Shahkhatuni

**Affiliations:** a Department of Chemical and Biomolecular Engineering, National University of Singapore E5, Engineering Drive 4 Singapore 117585 Singapore; b Scientific Technological Center of Organic and Pharmaceutical Chemistry 26 Azatutyan Street Yerevan 0014 Armenia astriksh@gmail.com

## Abstract

Visible light-driven *E*/*Z* photoisomerization is an attractive strategy for controlling reactivity and biological activity; however, targeted design of heterocyclic bioactive compounds requires a solid understanding of how substituent effects govern productive isomerization and competing photochemical pathways. Here, a series of 6-methyl-2-styryl-pyrimidine-4(3*H*)-one derivatives with previously reported antifungal and antimicrobial activity of *E*-isomers were studied by LED-NMR spectroscopy under visiblelight irradiation at 405–450 nm. Pronounced substituent-dependent differences were observed in the efficiency of *E*/*Z* photoisomerization, the stability of the corresponding *Z*-isomers, and the formation of [2 + 2] cycloaddition products. Derivatives bearing electron-donating groups showed particularly efficient photoresponse, with up to 95% conversion to the *Z*-isomer within 1–3 h at moderate irradiation power below 1 W. The resulting *Z*-isomers showed appreciable thermal stability, with half-lives ranging from several hours to several days. Quantum chemical calculations were used to rationalize the observed substituent effects and photoreaction outcomes. Among the computed descriptors, the oscillator strength *f*_S_1__(*E*) was found to be the most informative. This descriptor represented the absorption-related contribution to photoconversion, and favorable transition intensities were calculated for derivatives bearing OMe-type donor-substituted phenyl groups attached to the vinylene fragment. These results establish substituted 6-methyl-2-styryl-pyrimidine-4(3*H*)-ones as a visible light-responsive scaffold in which photoconversion, *Z*-isomer stability, and competing cycloaddition can be tuned by substituent design.

## Abstract 2

Substituent and solvent effects on photoisomerization and cyclodimerization were established for styryl pyrimidinones by LED-NMR spectroscopy. A low-cost computational workflow was developed to prioritize candidates for experimental screening.

## Graphical abstract

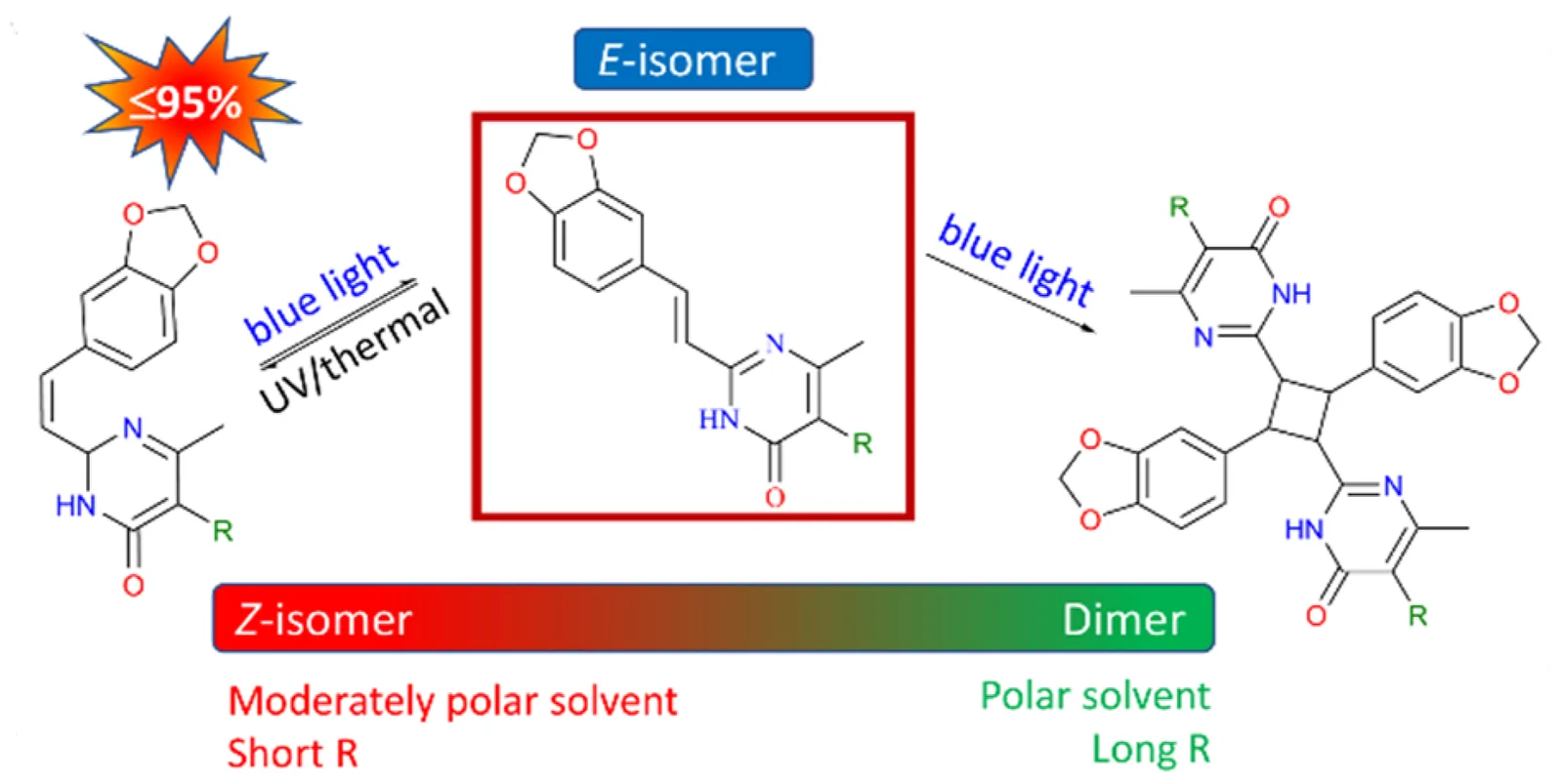

## Introduction

Visible light photochemistry of *E*/*Z* photoisomerization involving photoswitchable bioactive molecules has become an important direction in modern organic chemistry because distinct stereoisomeric states can be accessed from a single precursor, without *de novo* synthesis, and under mild conditions.^1–4^ Photoisomerizable compounds have broad use as molecular switches, smart materials, sensors, optoelectronic components, and photopharmacological tools.^5–11^ In biological systems, visible light activation is especially desirable because lower-energy irradiation is more compatible with sensitive functional groups and more suitable for biomedical contexts than ultraviolet light.^3,12^ However, efficient use of olefinic photoisomerization for biological applications requires more than demonstrating *E*/*Z* switching. The photostationary composition, *Z*-isomer stability, and competition with other photochemical processes should be understood and controlled.

The *E*/*Z* isomerization of C=C-containing scaffolds can strongly alter molecular shape, polarity, solubility, crystal packing, and chemical reactivity, and these changes may translate into modified biological or material properties.^13,14^ At the same time, photoirradiation of olefinic systems often gives competing or consecutive reactions, including intermolecular [2 + 2] photocycloaddition and intramolecular C–N photocyclization.^15–19^ These pathways can be synthetically valuable because functionalized cyclobutanes and fused heterocycles may be accessed under photochemical conditions.^20–23^ Consecutive photoisomerization and photocyclization has also been demonstrated for cyanostilbene under continuous irradiation at 365 nm in solution.^24^ Therefore, for a new photoswitchable scaffold, productive *Z*-isomer formation and competing cyclization or cyclodimerization should be considered as well.

Experimental conditions and molecular structure can strongly affect both the photostationary *E*/*Z* ratio and the reaction pathway. Solvent, temperature, irradiation wavelength, substituent electronics, and substituent position may influence the efficiency of photoisomerization, the lifetime of the *Z*-isomer, and the yield of dimer. NMR spectroscopy is well suited for this type of analysis because *E*/*Z* mixtures can be quantified directly.^25^ In addition, LED-NMR methods allow photochemical transformations to be monitored in real time inside the NMR tube, with simultaneous observation of *E*- and *Z*-isomers and any photocyclization or [2 + 2] cycloaddition products.^26–34^ This approach provides a direct experimental basis for connecting molecular structure, irradiation conditions, and the products of the photochemical process.

Styryl and pyrimidinone fragments are relevant motifs in bioactive molecular design. Compounds containing these units have been associated with anticancer activity, and photochemical conversion of a relatively inactive *E*-isomer into a biologically active *Z*-isomer has been proposed as a strategy for targeted phototherapy.^35–39^ Since the pyrimidine fragment is also a core structural element of native nucleotides, photoswitchable pyrimidine-based molecules represent an attractive class of photoresponsive compounds for bio-applications. Nevertheless, visible light photoisomerization of styryl-pyrimidinone systems has remained insufficiently characterized, particularly with respect to the substituent effect on *Z*-isomer formation, *Z*-isomer persistence, and competing [2 + 2] dimerization.

Here, LED-NMR spectroscopy was used to systematically analyze *E*/*Z* photoisomerization in a series of substituted 6-methyl-2-styryl-pyrimidine-4(3*H*)-ones. Antifungal and antimicrobial activity had recently been reported for the *E*-isomers of these compounds.^40,41^ Several derivatives underwent efficient photoisomerization under visible light irradiation, with high *Z*-isomer yields at the PSS or apparent PSS, depending on whether competing [2 + 2] cyclodimerization occurred. The effects of the adjacent substituent, wavelength, and solvent on the composition after irradiation, *Z*-isomer formation, and [2 + 2] dimerization were examined experimentally. The observed photochemical outcomes were then analyzed using a computationally efficient workflow in which conformer ensembles were generated from isomeric SMILES and Boltzmann-averaged vertical excitation energies and oscillator strengths were obtained with semiempirical methods. Using this analysis, we defined an applicability domain within which we could rationalize photoconversion using readily computed spectral descriptors without explicitly calculating photoisomerization quantum yields. Overall, substituted 6-methyl-2-styryl-pyrimidine-4(3*H*)-ones were established as visible light-responsive scaffolds in which *Z*-isomer formation, its stability, and competing cyclodimerization can be modulated by substituent and solvent effects.

## Methods

### Experimental details

A series of styryl-pyrimidines with various substituents was studied (Fig. 1). The compounds had previously been synthesized and characterized by NMR spectroscopy, with clear evidence that the synthetic methodology yielded compounds in the *E* configuration.^40,41^

**Fig. 1:**
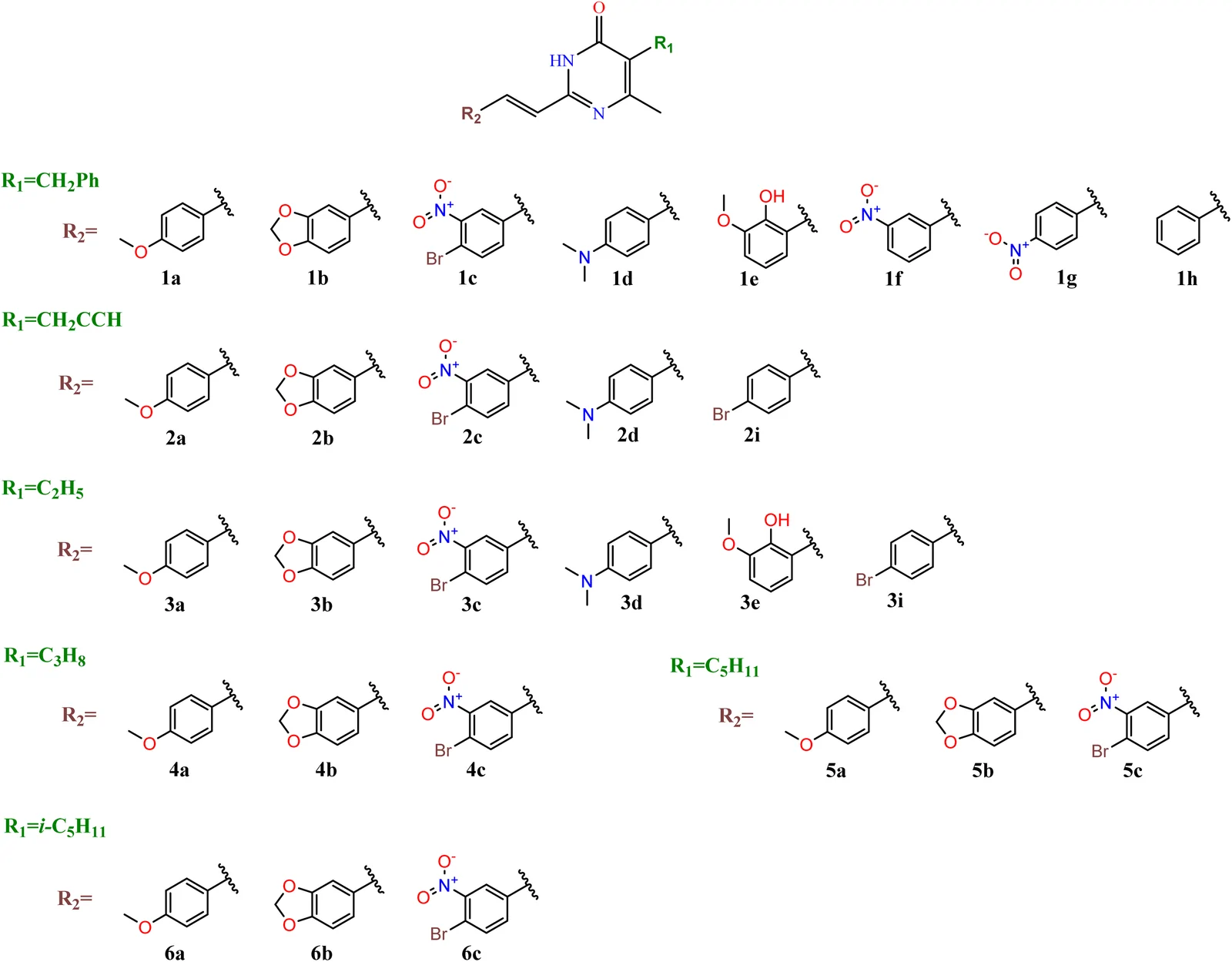
Structures and numbering of studied compounds.

LED irradiation was performed using Prizmatix Fiber Coupled Multi Wavelength LED Light Sources (FC5-LED) with LEDs at 365 nm, 395 nm, 405 nm, 420 nm, 450 nm, and 470 nm with powers of several hundreds of mW. Comparative azobenzene actinometry was used to estimate the photon fluxes of the primarily used 405, 420, and 450 nm LED sources, and values ranging from about 1.8 × 10^−7^ to 3.2 × 10^−7^ einstein s^−1^ were obtained. For illumination, a standard 5 mm NMR tube equipped with a coaxial insert was used. The sheathed tip of the optical fiber was placed inside the insert, and the sample was irradiated across its entire volume. The sample was irradiated inside the NMR tube either outside of the spectrometer (*ex situ*) or inside the spectrometer (*in situ*), and ^1^H spectra were periodically recorded to assess the effect of irradiation. If there was no effect, *i.e.* no signs of signals of other compounds or isomers, the irradiation wavelength was changed. When conversion to the *Z*-isomer was observed, irradiation was continued until further irradiation produced no qualitative changes in the composition. Depending on the compound and experimental conditions, a true *E*/*Z* PSS was not reached because of competing [2 + 2] cyclodimerization observed in some cases. In such cases, the final composition is referred to as an apparent PSS.

A 400 MHz Bruker AVANCE NEO spectrometer equipped with a Smart probe was used. ^1^H, ^13^C, HSQC, HMBC, and NOESY NMR spectra were recorded and processed with MestreNova. Typical ^1^H and ^13^C NMR spectra of the mixture containing the highest yield of the *Z*-isomer after irradiation are provided in the SI.

DMSO-*d*_6_, CDCl_3_, or DMSO-*d*_6_/CCl_4_ mixture were used depending on the solubility and purpose of the experiment. Irradiation was performed at room temperature, whereas NMR spectra were recorded at 30 °C. Studied compounds were dissolved in 0.5 ml of solvent with concentrations of compounds from 0.02 M to 0.1 M depending on solubility.

HRMS spectra were recorded using a Waters Xevo G3 UHPLC Q-Tof instrument. UV-vis spectra were recorded in chloroform using an Agilent Cary 60 spectrophotometer.

### Computational details

The computational datasets were constructed as follows. For *R*_1_ = Et, propargyl, and Bz, all nine *R*_2_ groups (a to i in Table 1) were included. For *R*_1_ = Pr, pentyl, and iso-pentyl, we considered *R*_2_ = 4-methoxyphenyl, 3,4-methylenedioxyphenyl, and 4-bromo-3-nitrophenyl. The resulting extended computational set comprised 36 compounds. Of these, 28 compounds with measured photoconversions constituted the full experimental dataset, with one selected observation per compound from Table 1. The primary fit comprised 25 compounds after exclusion of the three derivatives bearing *R*_2_ = 4-dimethylaminophenyl, as justified below. The fit including all 28 compounds is given in the SI. Data points corresponding to the DMSO-*d*_6_/CCl_4_ solvent systems were selected where available. For compounds not examined in DMSO-*d*_6_/CCl_4_, measurements in DMSO-*d*_6_ were used. When several measurements were available for the case of the preferred DMSO-*d*_6_/CCl_4_ solvent system, the result obtained after the longest irradiation time was selected. Overall, the goal was to select the most uniform solvent conditions permitted by differences in solubility and the available experimental data.

**Table 1:** Ratios of *E*-, *Z*-isomers, and of monomer-equivalent cyclodimerized material *D* (the corresponding molar fraction of the dimer is *D*/2), and total conversion (the sum of ratio of *Z*-isomer and *D*) in the mixtures obtained after reaching the (apparent) PSS

Compound	*E*, %	*Z*, %	*D*, %	Conversion, %	Wavelength, nm	Approximate time, h	Solvent
1a	19	79	2	81	405	10	DMSO-*d*_6_/CCl_4_ 1/1
	20	70	10	80	405	3	DMSO-*d*_6_/CCl_4_ 1/3
	17	67	16	83	405	4	DMSO-*d*_6_/CCl_4_ 1/3
1b	11	89	0	89	420	2	DMSO-*d*_6_/CCl_4_ 1/3
	68	17	15	32	420	3	DMSO-*d*_6_
1c	60	35	5	40	420	3	DMSO-*d*_6_/CCl_4_ 1/1
1d	55	40	5	45	450	2	DMSO-*d*_6_
1e	87	13	0	13	420	1	DMSO-*d*_6_
1f	90	4	6	10	420	3	DMSO-*d*_6_
1g	90	10	0	10	420	2	DMSO-*d*_6_/CCl_4_ 1/3
1h	90	6	4	10	365	2	DMSO-*d*_6_/CCl_4_ 1/3
2a	19	75	6	81	420	10	DMSO-*d*_6_/CCl_4_ 1/3
	12	71	17	88	405	4	DMSO-*d*_6_/CCl_4_ 1/3
2b	11	89	0	89	450	1	DMSO-*d*_6_/CCl_4_ 1/3
	71	12	17	29	405	3	DMSO-*d*_6_
2c	22	77	1	78	420	2	DMSO-*d*_6_/CCl_4_ 1/1
2d	60	40	0	40	420	2	DMSO-*d*_6_
2i	68	2	30	32	405	3	DMSO-*d*_6_
3a	11	75	14	89	405	3	DMSO-*d*_6_/CCl_4_ 1/3
3b	5	95	0	95	420	1	DMSO-*d*_6_/CCl_4_ 1/3
	81	6	13	19	405	4	DMSO-*d*_6_
	25	75	0	75	450	3	CDCl_3_
3c	45	55	0	55	420	2	DMSO-*d*_6_/CCl_4_ 1/1
	48	46	6	52	420	6	DMSO-*d*_6_/CCl_4_ 1/1
3d	53	45	2	47	420	2	DMSO-*d*_6_/CCl_4_ 1/1
	51	41	8	49	420	6	DMSO-*d*_6_/CCl_4_ 1/1
3e	88	11	1	12	405	2	DMSO-*d*_6_/CCl_4_ 1/1
3i	94	1	5	6	405	2	DMSO-*d*_6_/CCl_4_ 1/1
	90	1	9	10	405	6	DMSO-*d*_6_/CCl_4_ 1/1
4a	20	76	4	80	420	10	DMSO-*d*_6_/CCl_4_ 1/1
	20	77	3	80	405	2	DMSO-*d*_6_/CCl_4_ 1/3
	15	82	3	85	405	3	DMSO-*d*_6_/CCl_4_ 1/3
4b	25	3	72	75	420	7	DMSO-*d*_6_
	34	58	8	66	420	3	CDCl_3_
4c	20	80	0	80	420	2	DMSO-*d*_6_/CCl_4_ 1/3
	44	56	0	56	405	2	DMSO-*d*_6_
5a	17	79	4	83	420	10	DMSO-*d*_6_/CCl_4_ 1/1
5b	2	58	40	98	420	1	DMSO-*d*_6_/CCl_4_ 1/3
5c	62	30	8	38	405	2	DMSO-*d*_6_/CCl_4_ 1/1
6a	16	76	8	84	405	5	DMSO-*d*_6_/CCl_4_ 1/3
6b	19	2	79	81	420	4	DMSO-*d*_6_
	47	41	12	53	420	1	DMSO-*d*_6_/CCl_4_ 1/1
6c	45	50	5	55	405	3	DMSO-*d*_6_/CCl_4_ 1/1

Initial three-dimensional structures were generated from isomeric SMILES with Open Babel 3.1.0,^42^ followed by hydrogen addition and pre-optimization using UFF.^43^ Conformer ensembles were generated with CREST^44^ at the GFN2-xTB^45^ level with ALPB(DMSO) solvation.^46^ Conformer-level geometry optimizations, polarizability estimates, and Boltzmann weighting were carried out with xtb 6.7.1,^47^ while vertical excitation energies and oscillator strengths were computed with sTDA-xTB^48^ after xtb4stda preprocessing. Thermochemical calculations were conducted with ORCA 5.0.3.^49^ Statistical analysis, plotting, and cross-validation were performed with pandas,^50^ NumPy,^51^ statsmodels,^52^ scikit-learn,^53^ and Plotly.^54^ ChatGPT-5.5 was used for initial proofreading of some parts of the text and code refactoring; all scientific content, calculations, data interpretation, and final text were checked, edited, and approved by the authors.

## Results and discussion

The compounds shown in Fig. 1 can exist as *E*/*Z* stereoisomers. According to the previously reported NMR characterization, the isolated compounds were obtained as *E*-isomers using the selected synthetic procedure, consistent with the greater thermodynamic stability of the *E* configuration.^40,41^ Upon irradiation at an appropriate wavelength, *E* → *Z* photoisomerization was observed. After irradiation was discontinued, the *Z*-isomers slowly relaxed back to the thermodynamically more stable *E*-isomers, and heating accelerated the *Z* → *E* conversion. The *Z*-isomers were identified by NMR spectroscopy. The ethylene bridge protons showed a pronounced upfield shift, and the diagnostic ^3^*J*_HH_ values changed from 15.9–16.2 Hz for the *E*-isomers to 12.8–13.1 Hz for the *Z*-isomers. For example, irradiation of compound 3b at 420 nm for 70 min gave 95% conversion to the *Z*-isomer, as shown by the ^1^H NMR spectra in Fig. 2. Although this example demonstrated highly efficient photoisomerization, the *E*/*Z* conversion efficiency varied strongly across the series (Table 1). Importantly, effective photoisomerization was achieved in the visible light range, mainly at 405–450 nm.

**Fig. 2:**
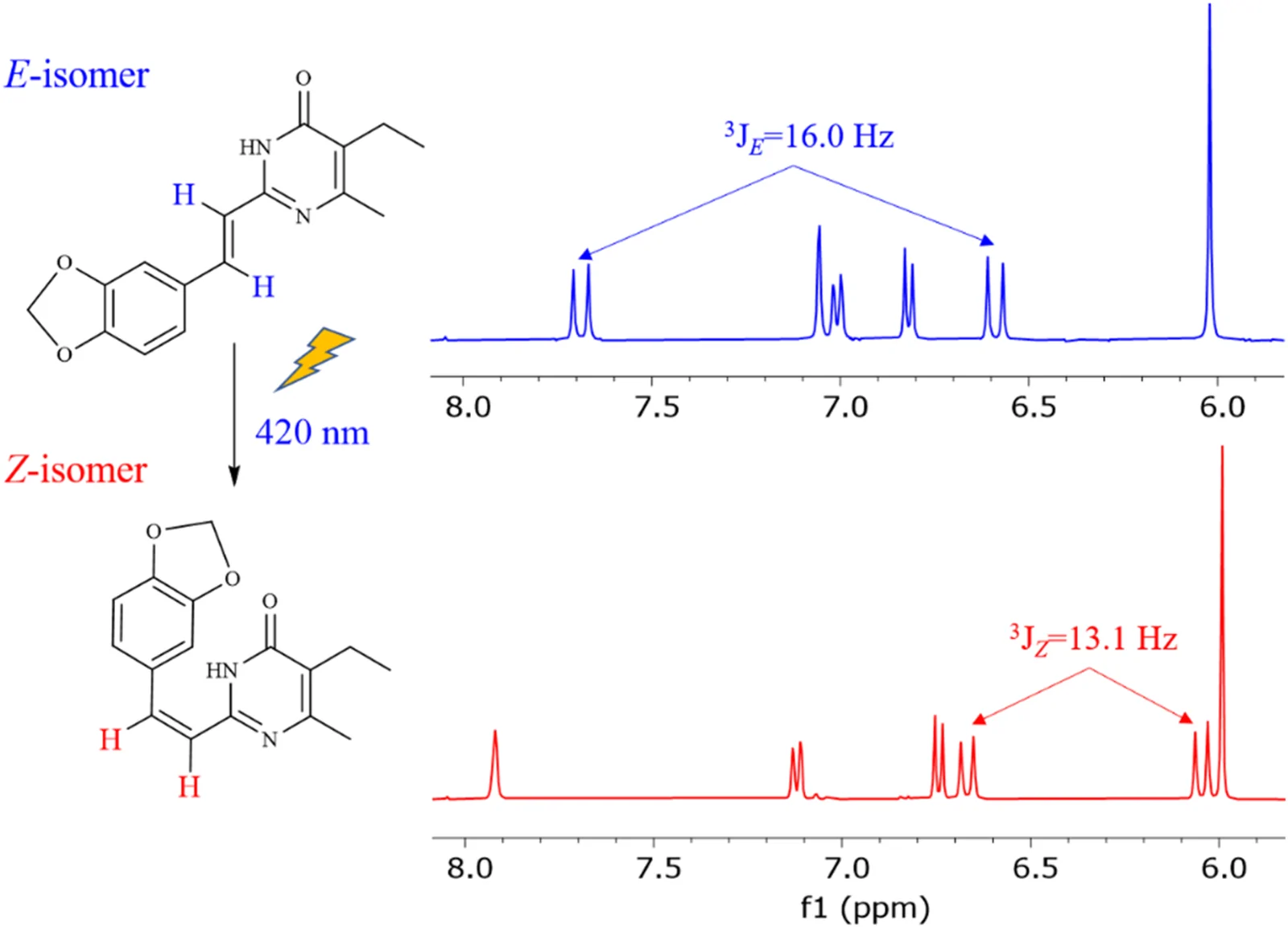
^1^H spectra of initial *E*-isomer of compound 3b (100%; top, blue) and the sample obtained after approximately 1 h of irradiation at 420 nm in DMSO-*d*_6_/CCl_4_ (95% *Z*-isomer; bottom, red). The signals of the ethylene bridge protons and their ^3^*J* spin–spin coupling constants are indicated.

In addition to *E*/*Z* photoisomerization, irradiation of several compounds initiated a subsequent [2 + 2] photocycloaddition pathway, analogous to stilbene photodimerization, and gave cyclobutane-containing dimers (Fig. 3). The dimer formation was supported by the appearance of two doublets of doublets at 4.09 and 4.65 ppm in the ^1^H NMR spectra, with coupling constants of approximately 9.9 and 7.5 Hz, assigned to cyclobutane-ring protons. The corresponding ^13^C NMR spectra contained new saturated-carbon signals at approximately 41 and 45 ppm. The assignment was further supported by 2D NMR experiments and HRMS, where ions corresponding to twice the mass of the starting monomer were detected (SI).

**Fig. 3:**
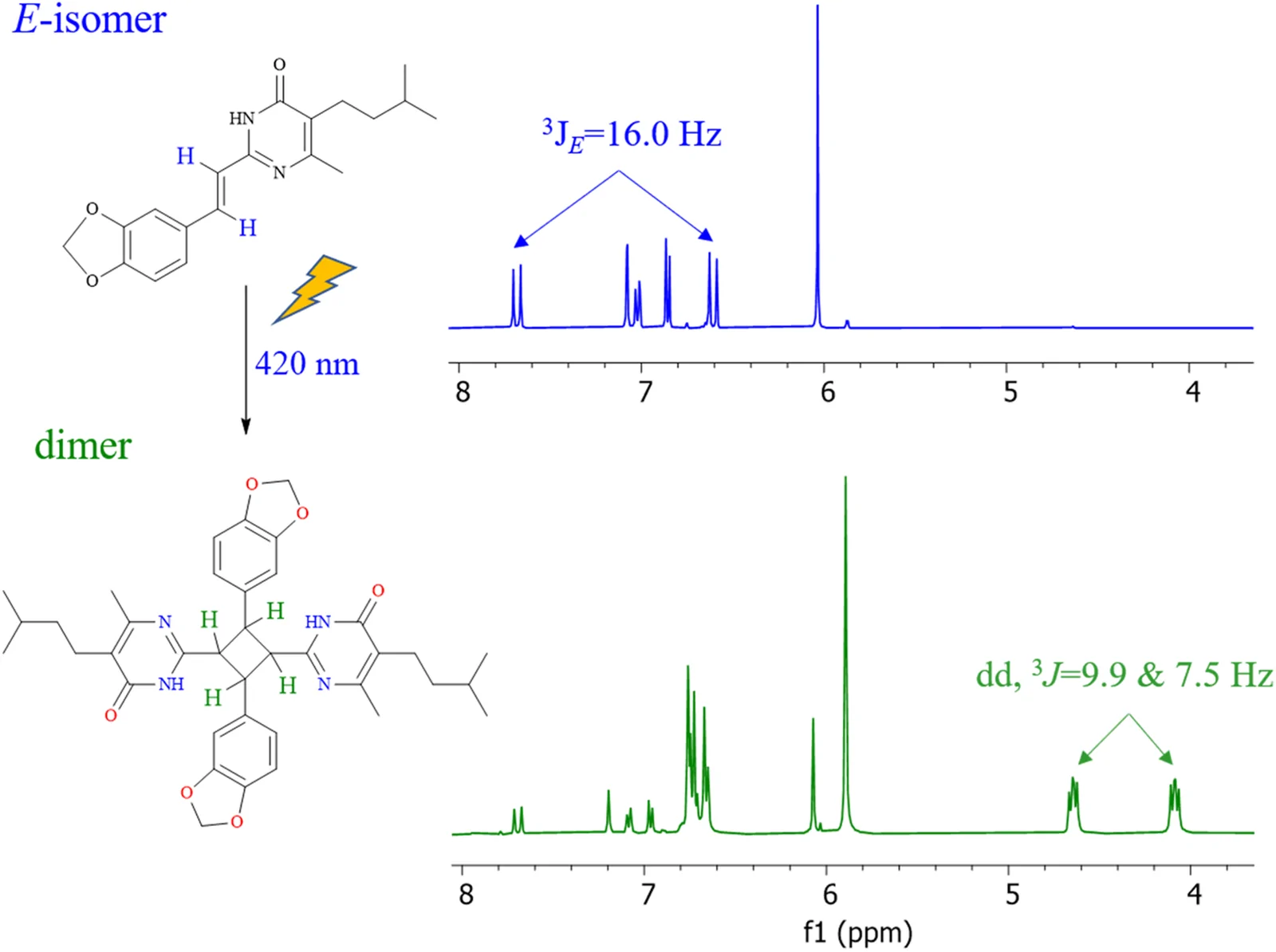
^1^H spectra of the initial *E*-isomer of compound 6b (100%; top, blue) and the sample obtained after 4 h of irradiation at 420 nm in DMSO-*d*_6_ (*D* = 79% monomer-equivalent cyclodimerized material; bottom, green). The signals of the ethylene bridge and cyclobutene ring protons and the corresponding ^3^*J* spin–spin coupling constants are indicated.

In principle, multiple regio- and stereoisomeric cyclobutane products can be formed by [2 + 2] photocycloaddition, including head-to-head and head-to-tail regioisomers and the corresponding *syn* and *anti* stereoisomers.^55^ Under the present conditions, however, a single dimeric product was formed predominantly whenever cycloaddition was observed, as indicated by the NMR spectra. The NOESY cross peaks between cyclobutane protons H_a_ and H_b_ and protons of the substituted phenyl ring were observed (Fig. S7 and S8), as previously reported for dimers formed from 2-(3,4-dimethoxystyryl)quinoxaline^55^ and other similar systems.^20^ Moreover, the measured vicinal spin–spin coupling constants of the cyclobutane protons, ^3^*J*_ab_ = ^3^*J*_a′b′_ = 9.9–10.1 Hz and ^3^*J*_ab′_ = ^3^*J*_a′b_ = 7.5–7.6 Hz, were close to those reported for these dimers (9.3–10.2 Hz and 7.1–7.6 Hz, respectively), for which the head-to-tail structure with r-ctt relative configuration was confirmed by X-ray analysis.^20,55^

The extent of cyclodimerization varied substantially across the series, from no detectable dimer to 79% monomer-equivalent conversion into the cycloaddition product. Compounds bearing the 3,4-methylenedioxyphenyl substituent were particularly prone to dimer formation, but the *R*_1_ substituent on the pyrimidinone scaffold also had a clear effect; in particular, benzyl-substituted derivatives formed significantly less dimer than the corresponding alkyl-substituted derivatives. The reaction outcome also depended strongly on solvent. In DMSO-*d*_6_, *E* → *Z* conversion was slower and dimer formation was enhanced, whereas DMSO-*d*_6_/CCl_4_ mixtures, especially DMSO-*d*_6_/CCl_4_ = 1 : 3, favored faster accumulation of the *Z*-isomer. Although the poor solubility of several derivatives in acetone and CDCl_3_ limited direct comparison, irradiation under mixing showed the same qualitative trend, in which CDCl_3_ favored *E* → *Z* photoisomerization, while DMSO-*d*_6_ and acetone-*d*_6_ favored cyclodimerization. In some cases, signals of the *Z*-isomer were absent or only minor, indicating that irradiation mainly led to the stable dimeric product rather than to accumulation of the free *Z*-isomer.

The effective irradiation wavelength and the maximum photoconversion were mainly governed by the *R*_2_ substituent. As shown in Table 1, total conversion ranged from 6 to 95%. Although an effect of electron-donating and electron-withdrawing groups was expected,^56^ no straightforward relationship was observed within the studied series. Derivatives bearing 4-methoxyphenyl, 3,4-methylenedioxyphenyl, and bromo-nitro-substituted phenyl groups showed more efficient photoisomerization and higher *Z*-isomer content, whereas phenyl, nitrophenyl, bromophenyl, and 2-hydroxy-3-methoxyphenyl derivatives gave lower *Z*-isomer ratios under irradiation at 365–470 nm. For several samples which do not tend to form dimers, the reverse *Z* → *E* photoisomerization was observed under 365 nm, although not quite effective (about 40–50% *Z*/*E*). The persistence of the *Z*-isomer was also *R*_2_-dependent. For the bromo-nitro-substituted derivative, the *Z*/*E* ratio remained nearly unchanged for several months in solution, and heating at 80 °C for 2 h had little effect. In contrast, derivatives bearing 4-methoxyphenyl and 3,4-methylenedioxyphenyl substituents underwent significant thermal *Z* → *E* back-isomerization at the same conditions (80 °C for 2 h) or when left overnight at room temperature.

The kinetic profiles also varied across the series. For compound 2b, *in situ*^1^H LED-NMR monitoring showed relatively rapid *E* → *Z* conversion under 450 nm irradiation, with no detectable side product formation during the monitored experiment (Fig. 4). In many other cases, however, several hours of irradiation were required to obtain the final compositions reported in Table 1. This timescale should be considered together with the moderate irradiation power of the LEDs used in the NMR setup, which was lower than that commonly used in preparative photochemistry. Prolonged irradiation often increased the amount of dimeric product, indicating that cyclodimerization can become more important after initial *Z*-isomer formation.

**Fig. 4:**
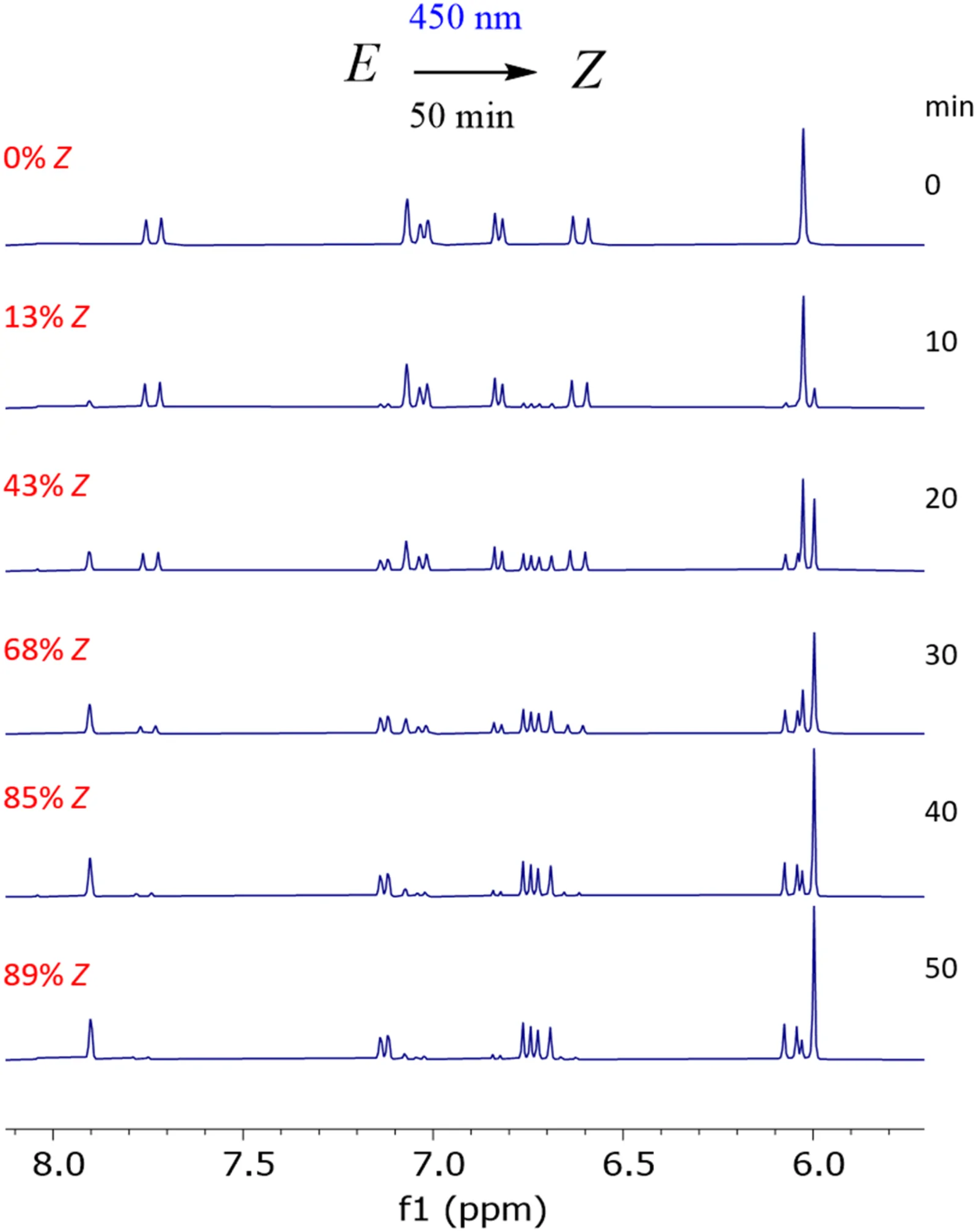
*In situ* LED-NMR monitoring of *E* → *Z* isomerization of compound 2b under irradiation at 450 nm in a DMSO-*d*_6_/CCl_4_ 1 : 3 mixture.

The real time change in the *E*/*Z* ratio for compound 3b under 420 nm and compound 2b under 450 nm irradiation is shown in Fig. 5. The profiles did not follow a simple monoexponential dependence, indicating that the process cannot be described as a simple first-order thermal reaction under the LED-NMR irradiation conditions. The profiles were consistent with bidirectional photochemical *E*/*Z* isomerization, in which both forward and reverse reactions contributed to the observed time dependence. Irradiation intensity and possible secondary photochemical pathways could also affect the profiles.

**Fig. 5:**
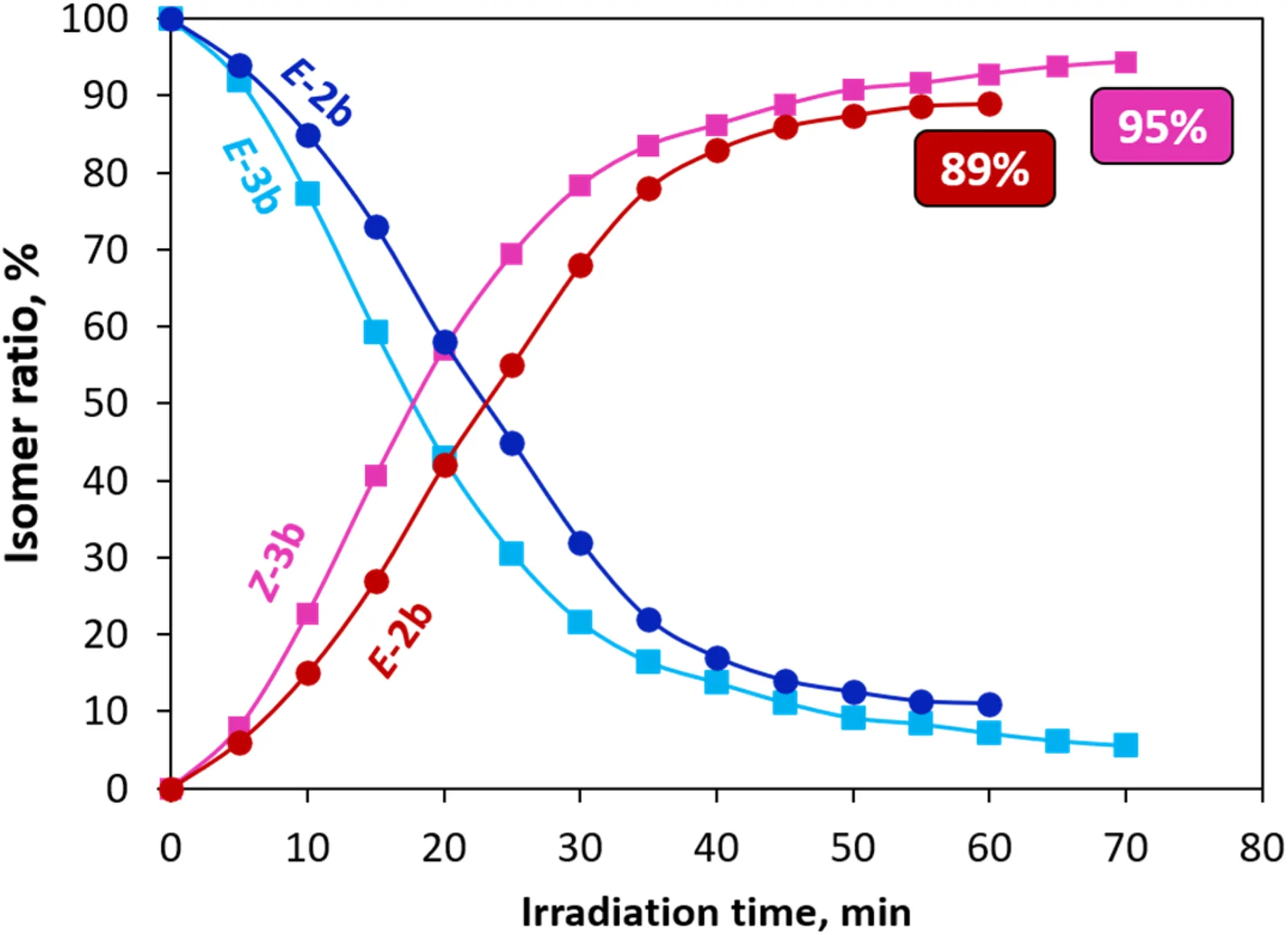
Changes in the *E*/*Z* ratios of compounds 2b and 3b during irradiation at 450 and 420 nm, respectively, in the NMR spectrometer (1 : 3 DMSO-*d*_6_/CCl_4_ mixture).

Repeated measurements for compound 3b at the same irradiation wavelength and in the same solvent system showed consistent reproducibility (Fig. S4). Although the time required to approach the PSS varied slightly among samples, the final *Z*-isomer yield was approximately 95% at concentrations from 10 to 40 mM.

The UV spectra of *E*-isomers of compounds 2a–2d are provided in the SI (Fig. S5). The absorption maxima of compounds 2a–2c were located in the 340–360 nm range, while compound 2d showed a maximum near 405 nm. Nevertheless, all four compounds underwent effective photoisomerization under irradiation in the 405–450 nm range. Therefore, no direct correlation was found between the wavelength of maximal absorption and the experimentally effective irradiation wavelength. This observation suggested that a more detailed descriptor-based analysis was required to rationalize the substituent dependence of photoconversion.

To understand why photoisomerization was more efficient for certain substituents and to identify descriptors associated with this variation, we developed a workflow for rapid computational screening of the photochemical properties of organic molecules with large one-photon absorption intensities. We applied this workflow to the *Z*/*E* photoisomerization of substituted 2-styryl-pyrimidine-4(3*H*)-ones (see Computational details and the discussion in the SI). Using the workflow, we automatically generated conformer ensembles in a model solvent with CREST^44^ and computed conformationally averaged vertical excitation energies, *E*_S_1__, oscillator strength values, *f*_S_1__, excitation wavelengths, and other photochemical and thermochemical descriptors, as well as ground state molecular polarizabilities.

As shown previously, the *Z*/*E* ratio attained under irradiation (*Z*/*E*-ratio) is determined by the relative absorption of the two isomers at the irradiation wavelength and by the forward and reverse photoisomerization quantum yields.^57–60^ Direct computational estimation of quantum yields remains challenging because excited state relaxation, competing nonradiative pathways, and access to conical intersection regions must be treated explicitly.^61–63^ We used sTDA-xTB, an ultrafast method for computing electronic spectra of large molecular systems,^48^ to estimate vertical excitation energies, with a focus on S_1_, and oscillator strengths for the conformer ensembles. Therefore, the readily computed *E*_S_1__ and *f*_S_1__ values were used as practical descriptors of the absorption-related contribution to photoisomerization efficiency. The model was designed for rapid screening of this contribution. Quantum yields, excited state relaxation, and branching among competing photochemical pathways were outside the scope for the model intended for screening within the applicability domain defined below. We allowed the irradiation wavelength to vary in the analyzed dataset because the correlation was intended to describe final photoconversion under compound-specific conditions rather than at some fixed wavelength. This treatment was consistent with the compound-dependent energy and wavelength of the calculated S_0_ → S_1_ transition, whose intensity was represented by the conformer-averaged *f*_S_1__ descriptor.

We then used the activity model dataset to examine the relationships between the computed descriptors and the selected NMR-derived photoconversions (see the supplementary spreadsheet for the underlying data). In Fig. 6a, the NMR-derived photoconversion defined as the sum of the *Z*-isomer and monomer-equivalent dimer percentages was plotted against conformer-averaged *f*_S_1__(*E*), for which the strongest correlation among the computed descriptors and photoconversion was obtained. In the SI, strong linear correlations between the *f*_S_1__ and *E*_S_1__ values of the *E*- and *Z*-isomers are discussed; therefore, *f*_S_1__ and *E*_S_1__ values of the *Z* form were not included in the correlation analysis.

**Fig. 6:**
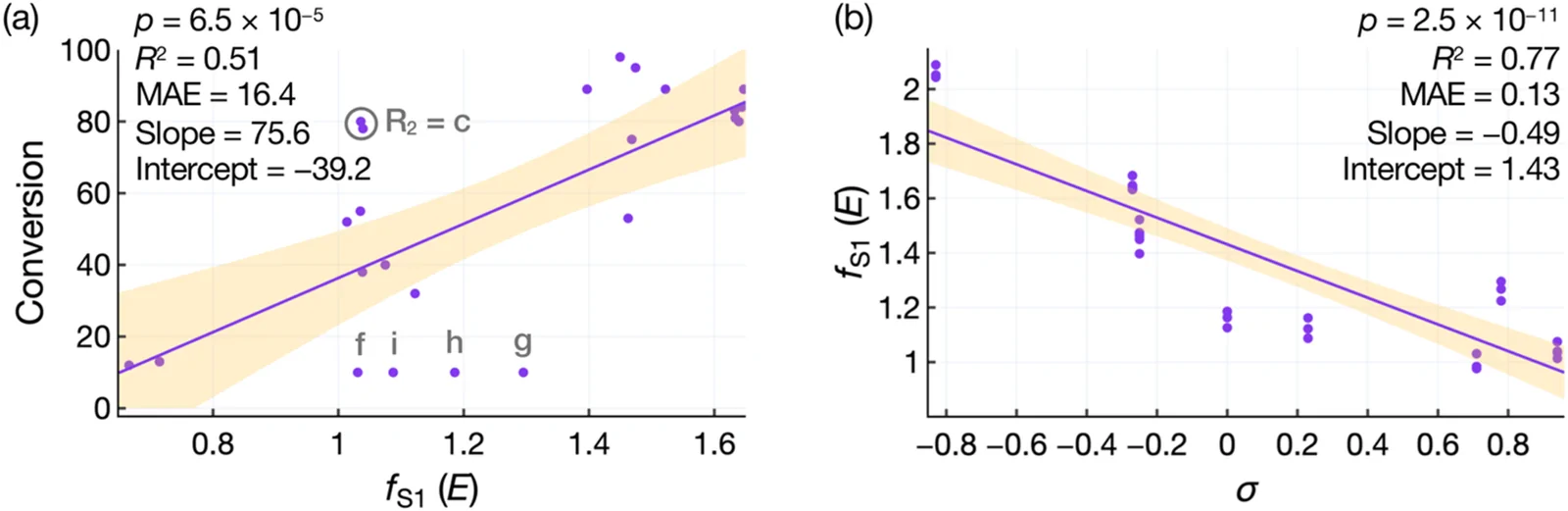
Correlation analyses for substituted 2-styryl-pyrimidine-4(3*H*)-ones. (a) NMR-derived photoconversion plotted against conformer-averaged *f*_S_1__(*E*). The labeled low-conversion entries correspond to *R*_2_ = 4-bromo-3-nitrophenyl (c), 3-nitrophenyl (f), 4-nitrophenyl (g), phenyl (h), and 4-bromophenyl (i). This linear relationship was fitted to the set of obtained experimental data (see Computational details and the supplementary spreadsheet for the dataset). (b) Linear correlation between conformer-averaged *f*_S_1__(*E*) and the sum of Hammett *σ* constants for substituents in the *R*_2_ group; the predictive quality of this model is discussed in the text. Shaded regions correspond to ordinary 95% confidence bands for the ordinary least-squares predictions.

Although a significant portion of the total variance in photoactivity remained unexplained by *f*_S_1__(*E*) alone (*R*^2^ = 0.51), as expected because quantum yields depend on excited state relaxation, competing nonradiative pathways, and access to reactive geometries, the linear fit was clearly statistically significant (*p* = 6.5 × 10^−5^). Part of the residual variance arose from moderate-*f*_S_1__(*E*) 2-styryl-pyrimidine-4(3*H*)-ones bearing Br and NO_2_ substituents, which appeared as outliers. However, this deviation may be associated with enhanced intersystem crossing reported for bromostilbene and nitrostilbene derivatives, while NO_2_-substituted push–pull stilbenes may also undergo charge transfer and NO_2_-torsional relaxation; such substituent-specific decay channels can compete with productive double bond photoisomerization.^64–66^ The three 4-dimethylaminophenyl-substituted derivatives had the highest *f*_S_1__(*E*) values among the compounds considered, and the model therefore predicted high photoconversion, whereas only moderate photoconversion was observed. Their inclusion weakened the otherwise strong statistical significance of the model (*p* = 6.5 × 10^−5^), as discussed in the SI. For the 4-dimethylaminophenyl group, deviation from the *f*_S_1__-based model can be expected because donor–acceptor stilbene-type chromophores bearing NMe_2_ substituents have been reported to undergo charge transfer-associated torsional relaxation, which can compete with productive double bond photoisomerization.^67,68^ Because this additional pathway was neither represented by the *f*_S_1__-based model nor included in its intended scope as requiring more complex calculations, the NMe_2_-substituted derivatives were excluded from the fitted dataset. The fits obtained with and without these derivatives are reported in the SI. A statistically significant dependence of the *Z*-isomer yield on *f*_S_1__(*E*) was also obtained, as discussed in the SI. Therefore, within the intended applicability domain, greater photoactivity was generally observed for substituted 2-styryl-pyrimidine-4(3*H*)-ones with higher *f*_S_1__(*E*), whereas deviations from the obtained relationship could be expected for Br- and NO_2_-substituted phenyl groups because productive *Z*/*E* photoisomerization could be suppressed by substituent-specific excited state decay pathways.

It should be noted that substituted 2-styryl-pyrimidine-4(3*H*)-ones with the donating *R*_2_ = 3,4-methylenedioxyphenyl group and longer aliphatic *R*_1_ = Pr, pentyl, and iso-pentyl substituents exhibited a stronger tendency toward dimerization following photoisomerization. For *R*_2_ = 4-methoxyphenyl, alkyl *R*_1_ substituents also drove higher dimer yields than *R*_1_ = Bz or propargyl (see Table 1). Although longer alkyl substituents clearly affect the (micro)solvation properties of these 2-styryl-pyrimidine-4(3*H*)-ones, this hypothesis was left for further research because multiple factors contributing to photoisomerization and photocycloaddition must still be delineated.

The largest contribution to *f*_S_1__(*E*) in the studied substituted 2-styryl-pyrimidine-4(3*H*)-ones appeared to arise from the electronic effect of the *R*_2_ group. In Fig. 6b, the linear correlation (*p* = 2.50 × 10^−11^) between *f*_S_1__(*E*) and the sum of Hammett *σ* constants of the substituents in the *R*_2_ group (using *σ*_p_ and *σ*_m_ values from ref. 69) is shown for the extended computational set (defined above; see the supplementary spreadsheet for data). Accordingly, with *R*^2^ = 0.77 and a low MAE of 0.13, the electronic effect of *R*_2_ was identified as the main factor associated with variation in the absorption intensity descriptor, *f*_S_1__(*E*), in the extended computational set of substituted 2-styryl-pyrimidine-4(3*H*)-ones. By contrast, no statistically meaningful dependence of the vertical S_1_ excitation energy, *E*_S_1__(*E*), on the Hammett *σ* parameter was found. The corresponding linear fit gave *R*^2^ = 0.006 and *p* = 0.668, with a slope statistically indistinguishable from zero, indicating that the *R*_2_ electronic effect primarily modulated the transition intensity rather than the vertical excitation energy. The predictive accuracy of the proposed linear approximation for *f*_S_1__(*E*) was assessed by 5-fold cross-validation after the *R*_2_ = e entries had been excluded from fitting and validation because no corresponding Hammett *σ* parameter was available (see below). Across the individual folds, MAE ranged from 0.11 to 0.18 and RMSE from 0.13 to 0.21, while the pooled out-of-fold predictions gave MAE = 0.14 and RMSE = 0.16. The fold-trained slopes were stable (−0.49 ± 0.02), indicating that the dependence of *f*_S_1__(*E*) on the *R*_2_ electronic effect was not dominated by a particular data split.

Notably, the highest *f*_S_1__(*E*) values were found for 4-dimethylaminophenyl-substituted 2-styryl-pyrimidine-4(3*H*)-ones, followed by compounds bearing donating 4-methoxyphenyl and 3,4-methylenedioxyphenyl substituents. By contrast, withdrawing Br (*σ*_p_ and *σ*_m_ equal to 0.23 and 0.39) and strongly withdrawing NO_2_ (*σ*_p_ and *σ*_m_ equal to 0.78 and 0.71) substituents, for which more complex behavior and poor predictability of photoconversion from *f*_S_1__(*E*) were observed, were associated with reduced transition intensity. The 2-hydroxy-3-methoxyphenyl-2-styryl-pyrimidine-4(3*H*)-one had a much smaller predicted *f*_S_1__(*E*) value and deviated from the trend in Fig. 6b, which we attribute to the *ortho*-position of the OH group (see the discussion in the SI). 2-styryl-pyrimidine-4(3*H*)-ones bearing donating OMe-type substituents on the Ph ring attached to the vinylene group, and possibly other electron-donating substituents, should be prioritized for experimental screening, whereas *ortho* substituents and long alkyl chains should be avoided due to the possible favorability of the subsequent dimerization. At the same time, experimental screening remains necessary because more complex photochemical processes may occur in compounds bearing the electron-donating NMe_2_ group.

## Conclusions

A series of substituted 6-methyl-2-styryl-pyrimidine-4(3*H*)-ones was shown to undergo visible light-driven *E*/*Z* photoisomerization under irradiation at 405–450 nm. LED-NMR spectroscopy allowed us to conduct direct monitoring of *E*- and *Z*-isomers together with competing [2 + 2] cyclodimerization products. The photochemical outcome depended strongly on both solvent and substituent structure. DMSO-*d*_6_ favored cyclodimerization and slowed accumulation of the *Z*-isomer, whereas DMSO-*d*_6_/CCl_4_ mixtures and CDCl_3_ favored *E* → *Z* photoisomerization. The highest *Z*-isomer yields were obtained for derivatives bearing 4-methoxyphenyl and 3,4-methylenedioxyphenyl substituents, with up to 95% conversion to the *Z*-isomer. In contrast, several phenyl, bromo-, nitro-, and 2-hydroxy-3-methoxy-substituted derivatives showed lower *Z*-isomer accumulation under the tested irradiation conditions.

To rationalize the substituent dependence, a computationally inexpensive workflow was developed that combined conformer ensemble generation, sTDA-xTB-based estimation of vertical excitation energies and oscillator strengths, and descriptor-based analysis of photoisomerization propensity. By comparing the computed descriptors with NMR-derived photoactivation outcomes, we identified an applicability domain and found that, within this domain, the conformer-averaged oscillator strength *f*_S_1__(*E*) had the strongest correlation with photoconversion. We therefore used *f*_S_1__(*E*) as a descriptor of the absorption-related contribution to photoconversion, while quantum yield estimation was outside the scope of the model. We identified the electronic effect of the *R*_2_ substituent as the main factor associated with variation in *f*_S_1__(*E*). Favorable transition intensities were obtained for derivatives bearing OMe-type donor-substituted phenyl groups attached to the vinylene fragment. From a practical design perspective, substituents on the adjacent aryl group associated with high *f*_S_1__(*E*), especially meta- and *para*-OMe-type donors, should be prioritized for experimental screening, while long adjacent alkyl substituents should be avoided when *Z*-isomer yield rather than cyclodimerization is the target. In such a way, candidates can be screened using input molecular structures in the form of isomeric SMILES strings on a standard laptop and prioritized for experimental testing or higher-level quantum chemical calculations.

## Author contributions

Mikhail V. Polynski: conceptualization, formal analysis, investigation, methodology, writing – original draft, writing – review & editing; Zaruhi A. Hovasyan: investigation; Arthur A. Harutyunyan: investigation, resources; Astghik A. Shahkhatuni – investigation, formal analysis, writing – review & editing, supervision, funding acquisition, project administration; Aleksan G. Shahkhatuni – methodology, formal analysis, conceptualization, validation, writing – original draft.

## Conflicts of interest

There are no conflicts of interest to declare.

## Supplementary Material

RA-OLF-D6RA06890A-s001

## Data Availability

The data underlying this study are available in the published article, its supplementary information (SI), and the Zenodo repository at https://doi.org/10.5281/zenodo.23167218. A description of the computational workflow is provided in the SI. The Python scripts are also available in the GitHub repository.^70^ Supplementary information: ^1^H, ^13^C and 2D NMR spectra of *E*/*Z* mixtures of compounds obtained after irradiation, and HRMS spectra of several mixtures showing [2 + 2] cycloaddition products. See DOI: https://doi.org/10.1039/d6ra06890a.
