# Supplementary material for: Substituent-controlled visible light photoisomerization and cyclodimerization of styryl pyrimidinones
Source: RSC Adv. 2026 Oct 8;16(46):60921–30. doi: 10.1039/d6ra06890a (PMC13647310; doi:10.1039/d6ra06890a)
Supplement: Supplementary file 1 [file RA-016-D6RA06890A-s001.pdf]

Supplementary Information (SI) for RSC Advances.
This journal is © The Royal Society of Chemistry

Substituent-Controlled Visible Light Photoisomerization and Cyclodimerization of Styryl Pyrimidinones

Mikhail V. Polynski,[1] Zaruhi A. Hovasyan[2], Arthur A. Harutyunyan[2], Astghik A. Shahkhatuni[2*], Aleksan G. Shahkhatuni[2]

[1]*Department of Chemical and Biomolecular Engineering, National University of Singapore; E5, Engineering Drive 4, Singapore 117585, Singapore*

[2]*Scientific Technological Center of Organic and Pharmaceutical, 26 Azatutyan street, Yerevan 0014, Armenia*

* *Corresponding author. A.A.S.: astriksh@gmail.com.*

Supplementary Information

2	Computational details
7	Reproducibility of E/Z photoisomerization for compound **3b**
8	UV-vis spectra of compounds **2a**-**2d**
9	[2+2] cycloaddition products
13	NMR spectra of compounds after irradiation
68	Mass spectra of selected [2+2] cycloaddition products
73	References

Computational Details

Input structures were generated from isomeric SMILES strings, with *E*/*Z* stereochemistry encoded. Open Babel 3.1.0[1] was used to convert the SMILES strings into three-dimensional XYZ geometries with explicit hydrogen atoms. The initial structures were then pre-optimized with the UFF force field,[2] and the resulting XYZ geometries were used as inputs for conformational sampling. Conformer ensembles were generated with CREST.[3] The CREST calculations were carried out with GFN2-xTB[4] and the ALPB implicit solvent model[5] for DMSO. The corresponding CREST output file, crest_conformers.xyz, was used in the subsequent calculations.

The conformer-level property calculations were carried out with an automated Python workflow. First, each crest_conformers.xyz file was split into individual conformer directories. Each conformer was then reoptimized with GFN2-xTB in xtb 6.7.1,[6] with ALPB(DMSO) solvation, tight geometry convergence, and Molden output generation. The total energy and static molecular polarizability were parsed from the output. Input for the simplified Tamm-Dancoff tight-binding calculations with sTDA-xTB[7] was then generated using xtb4stda. Vertical excitation energies, excitation wavelengths, and oscillator strengths up to 4.5 eV were subsequently computed. For each conformer, the lowest energy bright excitation was selected. The corresponding vertical S1 excitation energy, E_{S1}, excitation wavelength, and oscillator strength, f_{S1}, were then subjected to Boltzmann weighting. In addition, spectral "detunings" from 405, 420, and 450 nm irradiation can be computed automatically as the difference between E_{S1} and the photon energy at the corresponding wavelength. Conformer weights were obtained from GFN2-xTB electronic energies at 298.15 K, and Boltzmann-weighted ensemble values were computed for E_{S1}, f_{S1}, molecular ground state polarizability, and the detuning descriptors. For each compound, the conformer properties and the ensemble row were written to a CSV table, and the ensemble rows from all compound folders were pooled into ensembles_pooled.csv files for subsequent processing.

Thermochemical corrections were also computed at the GFN2-xTB level for lowest-energy conformers of *E* and *Z* isomers. The corresponding optimized geometries were reoptimized in ORCA 5.0.3[8] using TightOpt convergence criteria, after which vibrational frequencies were calculated using the finite-difference procedure (NUMFREQ). Quasi-rigid rotor-harmonic-oscillator thermochemical corrections were then evaluated using the otherm code[9] at 298.15 K, with the 1 M standard state. To estimate bulk solvent effects, xTB single-point calculations were

carried out on the ORCA-optimized geometry in the gas phase and with ALPB(DMSO), and the difference between these two electronic energies was added to the gas-phase QRRHO Gibbs free energy. These values were used to calculate the free energy of *E*/*Z*-isomerization.

Data analysis and plotting were carried out in a Jupyter notebook with pandas,[10] NumPy,[11] statsmodels,[12] Plotly,[13] and scikit-learn.[14] The Activity_model worksheet of the supplementary .xlsx table was used for correlations involving the NMR-derived photoconversion and *Z*-isomer yield, whereas the Raw_data worksheet was used for correlations between computed descriptors and for the Hammett-like analysis of the substituent electronic effect. Linear fits were obtained by ordinary least squares with an intercept term included. For each fit, the intercept, slope, 95% confidence intervals, F-test p-value, R^2, MAE, and RMSE were computed. Plots were prepared with Plotly as scatter plots containing the fitted OLS line and the ordinary 95% confidence band. The internal predictive consistency of the Hammett-based linear approximation for $f_{S1}(E)$ was evaluated by 5-fold cross-validation in scikit-learn. A shuffled KFold split with n_splits = 5 and random_state = 1 was used. The plotted line for this analysis was obtained from the OLS fit to all data, whereas the CV metrics were computed separately from pooled out-of-fold predictions.

Descriptor Selection and Scope of the Photoactivity Model

A statistically significant linear correlation between the f_{S1} values of the *E* and *Z* isomers was found within the extended computational set of 2-styryl-pyrimidine-4(3H)-ones (Figure S1a), and a similar correlation was found for E_{S1} (Figure S1b). The full list of R_1 and R_2 groups considered in this set is discussed in the main text and listed in the supplementary .xlsx table. Because of this linear dependence, *Z*-isomer descriptors were not included in the models presented in the main text. The primary aim was to develop a simple and computationally efficient model for screening the absorption-related contribution to photoconversion and rationalizing the electronic effect of substituents on the phenyl group attached to the vinylene moiety. Only *E*-isomer properties were therefore considered. Quantum yields and substituent-specific excited state relaxation pathways were outside the scope of the model.

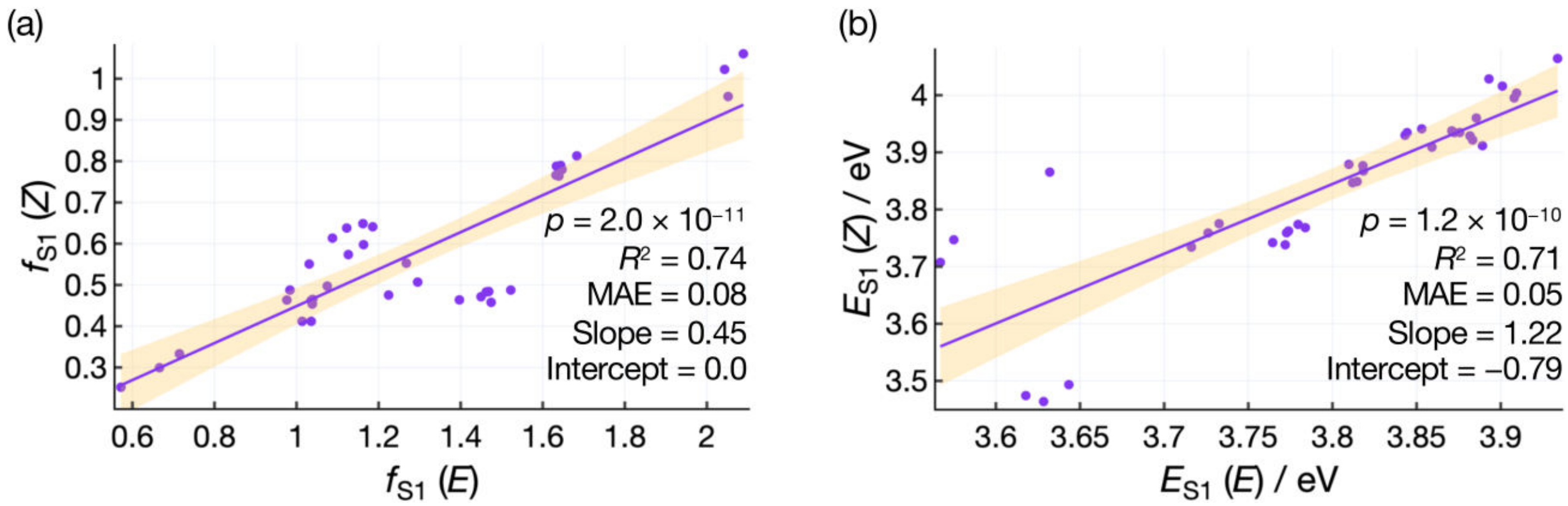

Figure S1. Linear correlations between *E*- and *Z*-isomer descriptors in the extended computational set of substituted 2-styryl-pyrimidine-4(3H)-ones. The shaded regions correspond to the ordinary 95% confidence bands for the mean ordinary least squares (OLS) predictions.

In Figure S2a, photoconversion was plotted for the dataset the entries bearing R_2 = 4-dimethylaminophenyl included in the fit. Previous studies on donor-acceptor stilbene-type chromophores bearing NMe_2 substituents showed that charge transfer-associated torsional relaxation can compete with productive photoisomerization of double bonds.[14–16] Accordingly, lower conversions were obtained for 2-styryl-pyrimidine-4(3H)-ones bearing R_2 = 4-dimethylaminophenyl than would be expected from the computational model. Nevertheless, the dependence of photoconversion on $f_{S1}(E)$ remained statistically significant, although substantially weakened, when these three entries were included (R^2 = 0.20, p = 0.017, slope = 36.3, 95% confidence interval (CI) = 7.1-65.5). After exclusion of these entries, the model presented in the main text gave R^2 = 0.51, p = $6.5 \cdot 10^{-5}$, and the slope of 75.6 (95% CI = 43.4-107.7). The confidence intervals of the two slopes overlapped. Therefore, the association between $f_{S1}(E)$ and photoconversion was also observed in the dataset including the 4-dimethylaminophenyl-substituted derivatives but was considerably stronger within the intended applicability domain. Therefore, 4-dimethylaminophenyl derivatives were considered beyond this domain and were excluded from the main analysis.

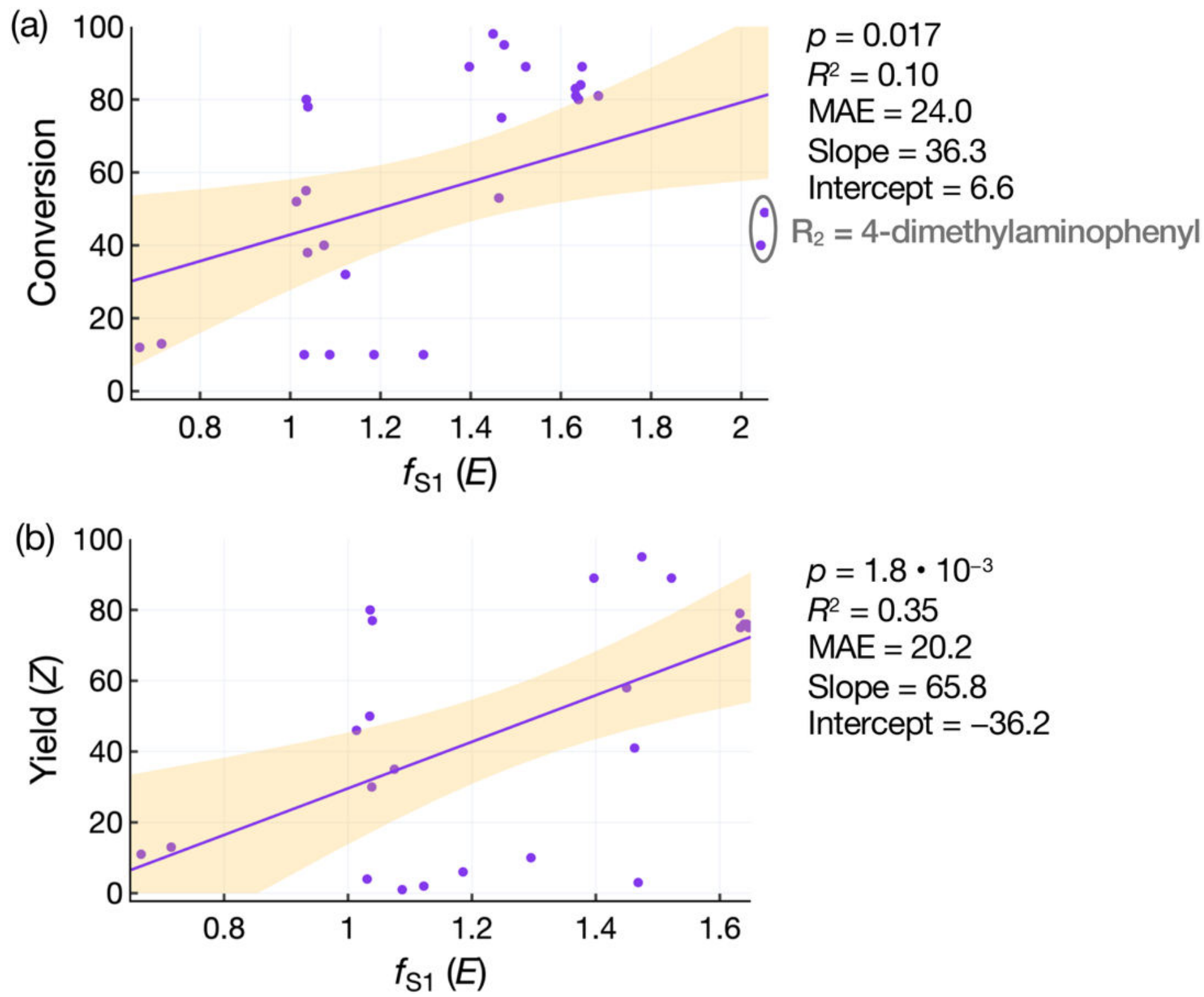

Figure S2. Correlations between conformer-averaged $f_{S1}(E)$ and NMR-derived photoactivation outcomes for substituted 2-styryl-pyrimidine-4(3H)-ones: (a) total photoconversion with the entries corresponding to 4-dimethylaminophenyl-substituted derivatives included and highlighted; (b) *Z*-isomer yield after exclusion of the same entries. The shaded regions correspond to the ordinary 95% confidence bands for the OLS predictions.

In Figure S2b, the *Z*-isomer yield, rather than total photoconversion defined as the sum of the *Z*-isomer and monomer-equivalent dimer percentages, was plotted against $f_{S1}(E)$. The resulting dependence remained statistically significant but was weaker. Using the *Z*-isomer yield data gave $R^2 = 0.35$ and $p = 1.8 \cdot 10^{-3}$, compared with $R^2 = 0.51$ and $p = 6.5 \cdot 10^{-5}$ for the case of total photoconversion. In most cases in which cyclodimerization occurred, the dimer remained a minor product. Substantial dimer formation was observed for **2i**, **4b**, and **5b**, with dimer percentages of 30%, 72%, and 40%, respectively, thereby weakening the correlation for the Z-isomer yields. The stronger association with total photoconversion was consistent with the intended utility of $f_{S1}(E)$ as the descriptor of the absorption-related contribution, while differentiating *Z*-isomer and dimer yields required the consideration of subsequent reaction pathways.

In Figure S3, the negative linear correlation between $f_{S1}(E)$ and the sum of Hammett σ constants of the para- and meta-substituents in the R_2 group is plotted with R_2 = 2-hydroxy-3-methoxyphenyl included. This analysis was used to assess why the favorable donating character of this substituent did not drive stronger photoactivity. Because no σ value was available for the ortho-OH group, the corresponding para-OH value from Ref.[17] was used. However, the conformer ensemble-averaged $f_{S1}(E)$ value remained below the main trend irrespective of its position on the horizontal axis. Consistently, the experimental yields of the Z isomer did not exceed 13%, indicating that $f_{S1}(E)$ indeed was a useful descriptor of photoactivity in this case. Therefore, despite the donating electronic effect of R_2 = 2-hydroxy-3-methoxyphenyl, this substituent was considered unfavorable for the design of efficient photoisomerizing substituted 2-styryl-pyrimidine-4(3H)-ones.

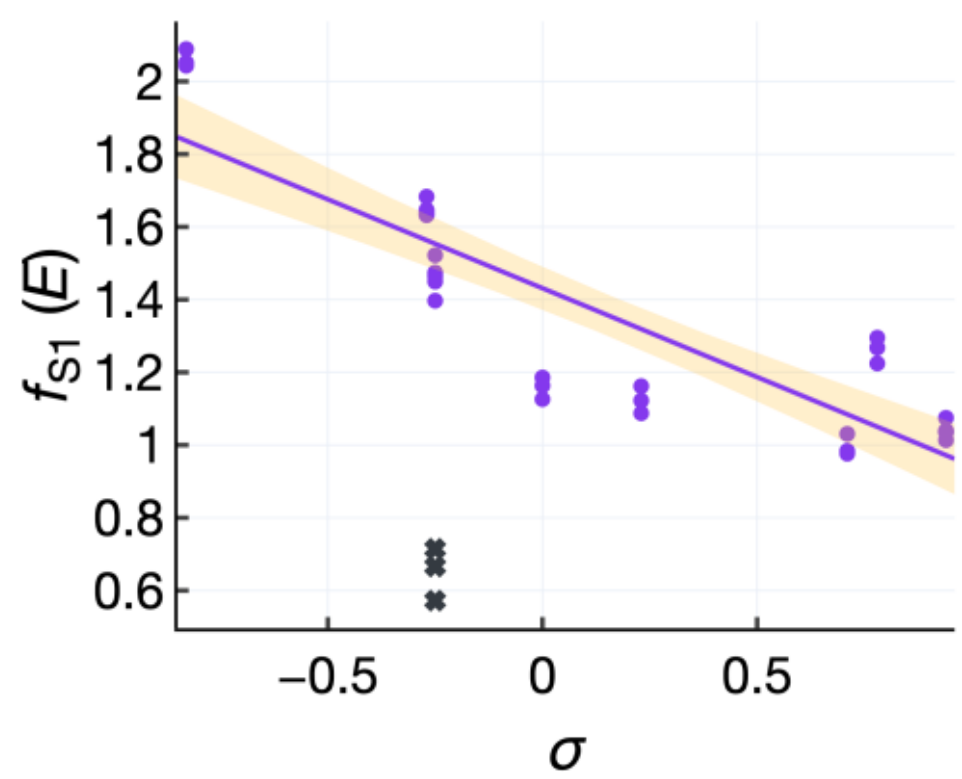

Figure S3. Linear correlation between conformer-averaged $f_{S1}(E)$ and the sum of Hammett σ constants for substituents in the R_2 group, with the R_2 = 2-hydroxy-3-methoxyphenyl entries included and shown as gray crosses. The shaded region corresponds to the ordinary 95% confidence band for the OLS prediction.

Reproducibility of *E/Z* photoisomerization for compound 3b

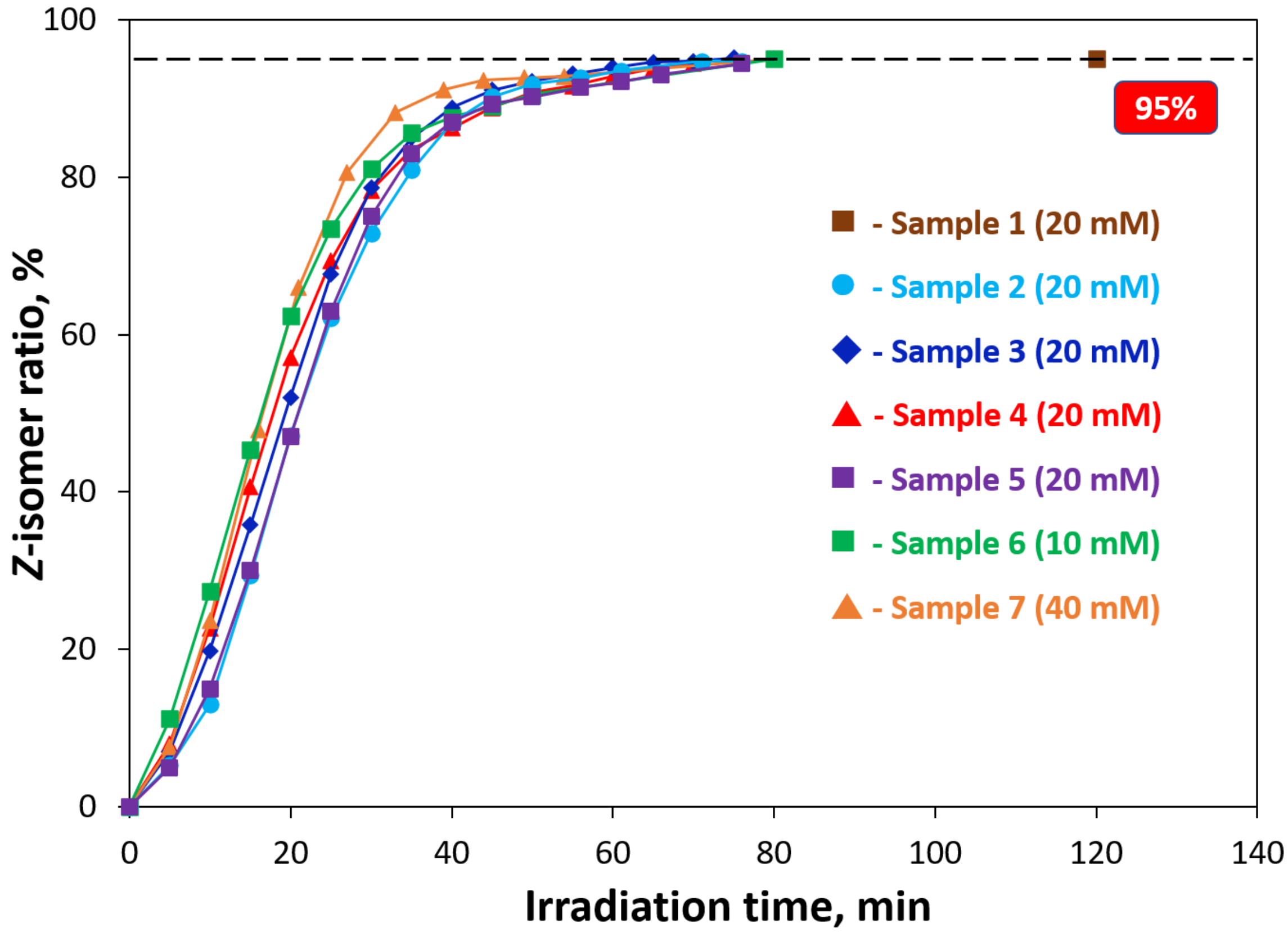

Figure S4. *In situ* LED-NMR monitoring of several samples of compound **3b** irradiated with 420 nm LED lamp in DMSO-d_6/CCl_4 1/3 at various concentrations. Sample 1 was irradiated continuously for 2 h.

UV-vis spectra of compounds 2a-2d

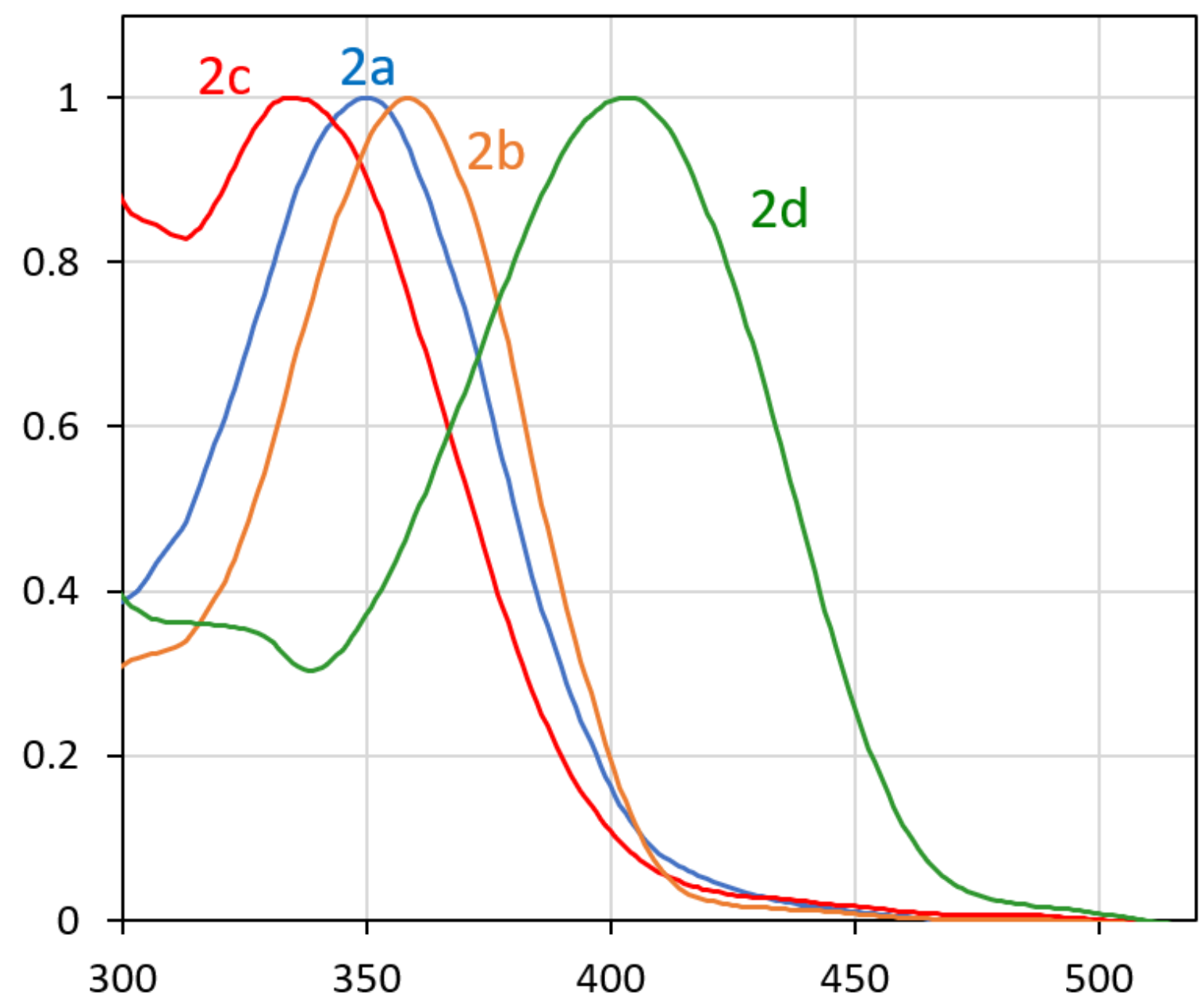

Figure S5. UV-vis spectra of the *E*-isomers of compounds **2a-2d.** Spectra were recorded in $CHCl_3$ using a 1 cm cuvette and normalized for comparison.

Table S1. Maximum absorption wavelengths λ_{max} and corresponding molar extinction coefficients ε_{max} of compounds.

Compound	λ_{max}, nm	ε_{max}, M^{-1} cm^{-1}
**2a**	350	38548
**2b**	358	32751
**2c**	336	16322
**2d**	405	16389

[2+2] cycloaddition products

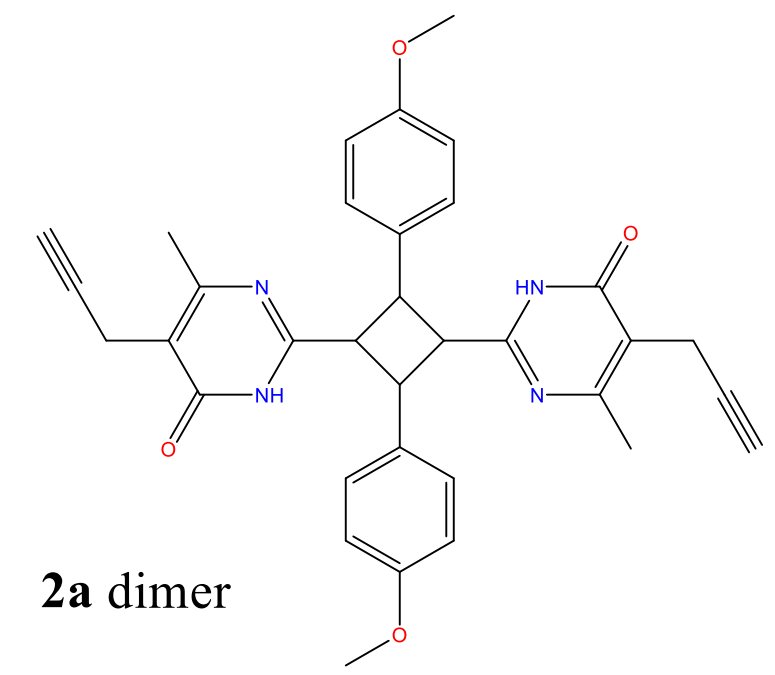

2a dimer

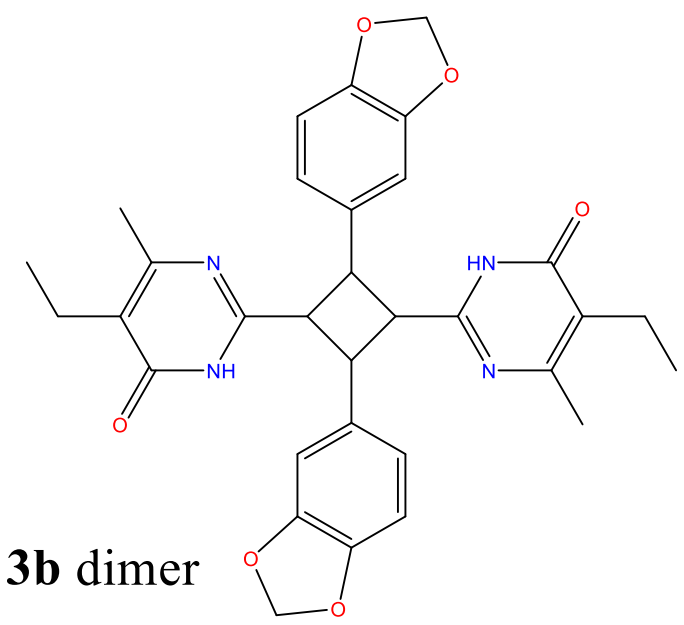

3b dimer

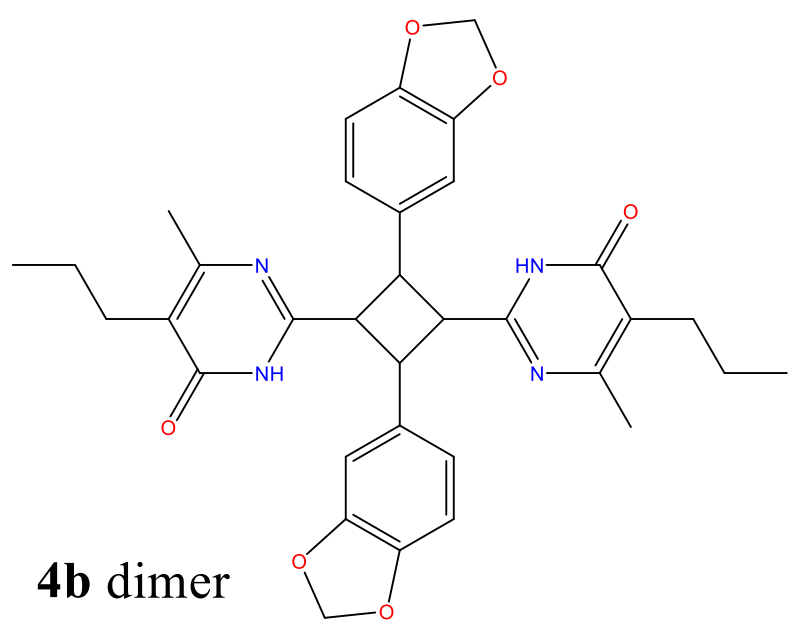

4b dimer

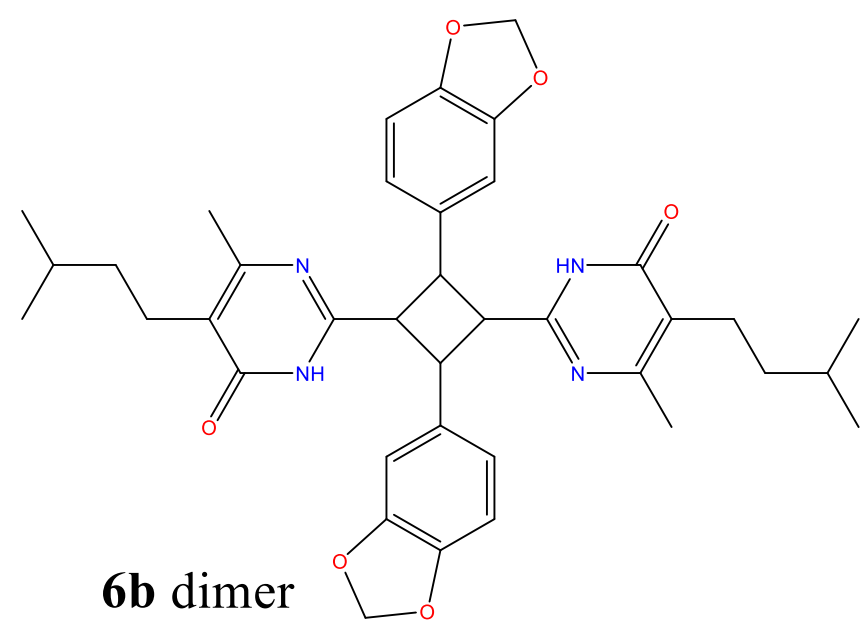

6b dimer

Figure S6. Structures of the [2+2] cycloaddition products (dimers) of studied compounds.

Table S2. Characteristic NMR and HRMS parameters of dimers, where *D* denotes monomer-equivalent cyclodimerized material.

Compound	*D*, %	NMR chemical shifts, ppm		Molecular Formula	HRMS, $[M+H]^+$	
		δ_H	δ_C		Calculated	Found
**2a**	17	4.13 & 4.73		$C_{34}H_{32}N_4O_4$	561.2502	561.2505
**3b**	19	4.09 & 4.65	44.8 & 41.3	$C_{32}H_{32}N_4O_6$	569.2400	569.2411
**4b**	72	4.09 & 4.65	44.8 & 41.3	$C_{34}H_{36}N_4O_6$	597.2713	597.2723
**6b**	79	4.09 & 4.65	44.8 & 41.3	$C_{38}H_{44}N_4O_6$	653.3339	653.3340

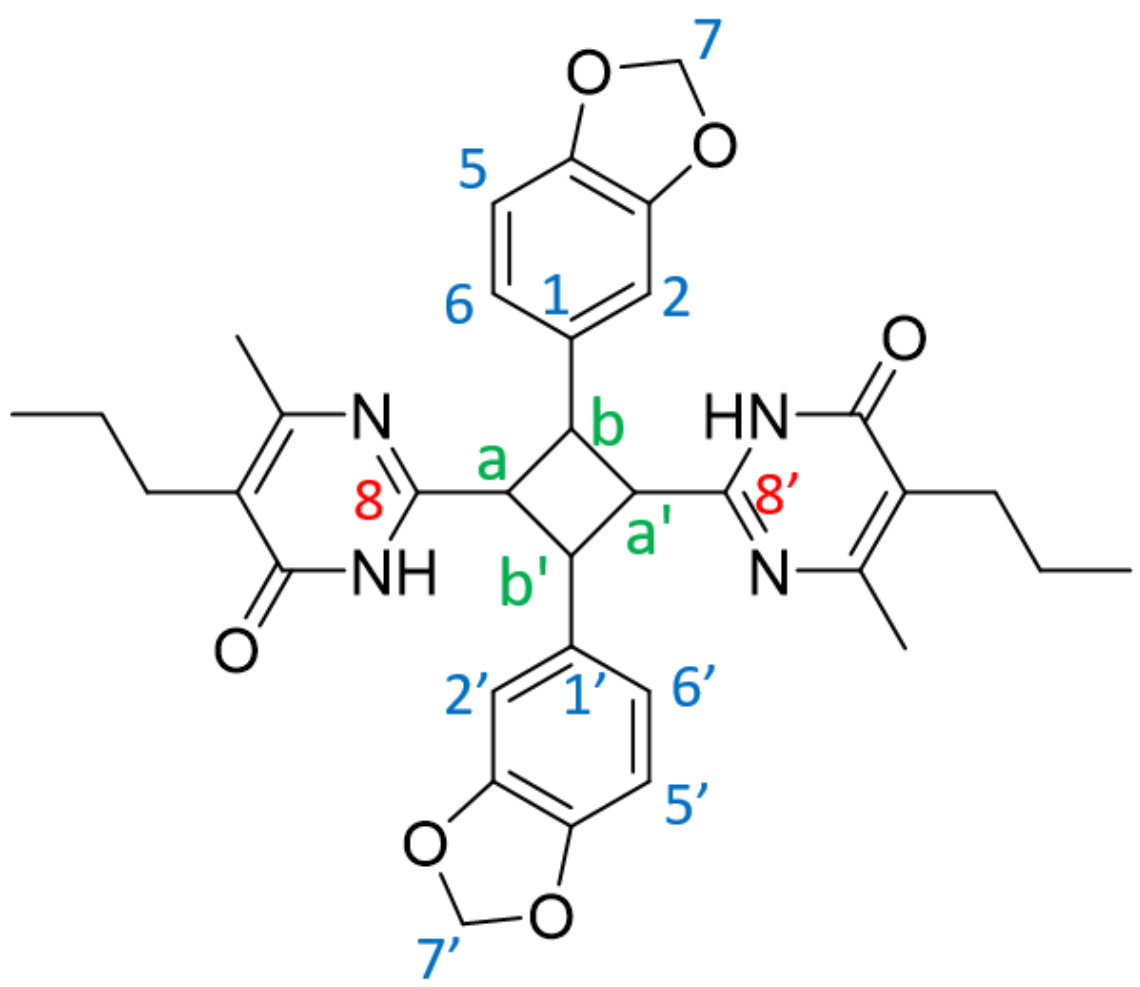

Figure S7. Numbering of [2+2] cycloaddition product of compound **4b.**

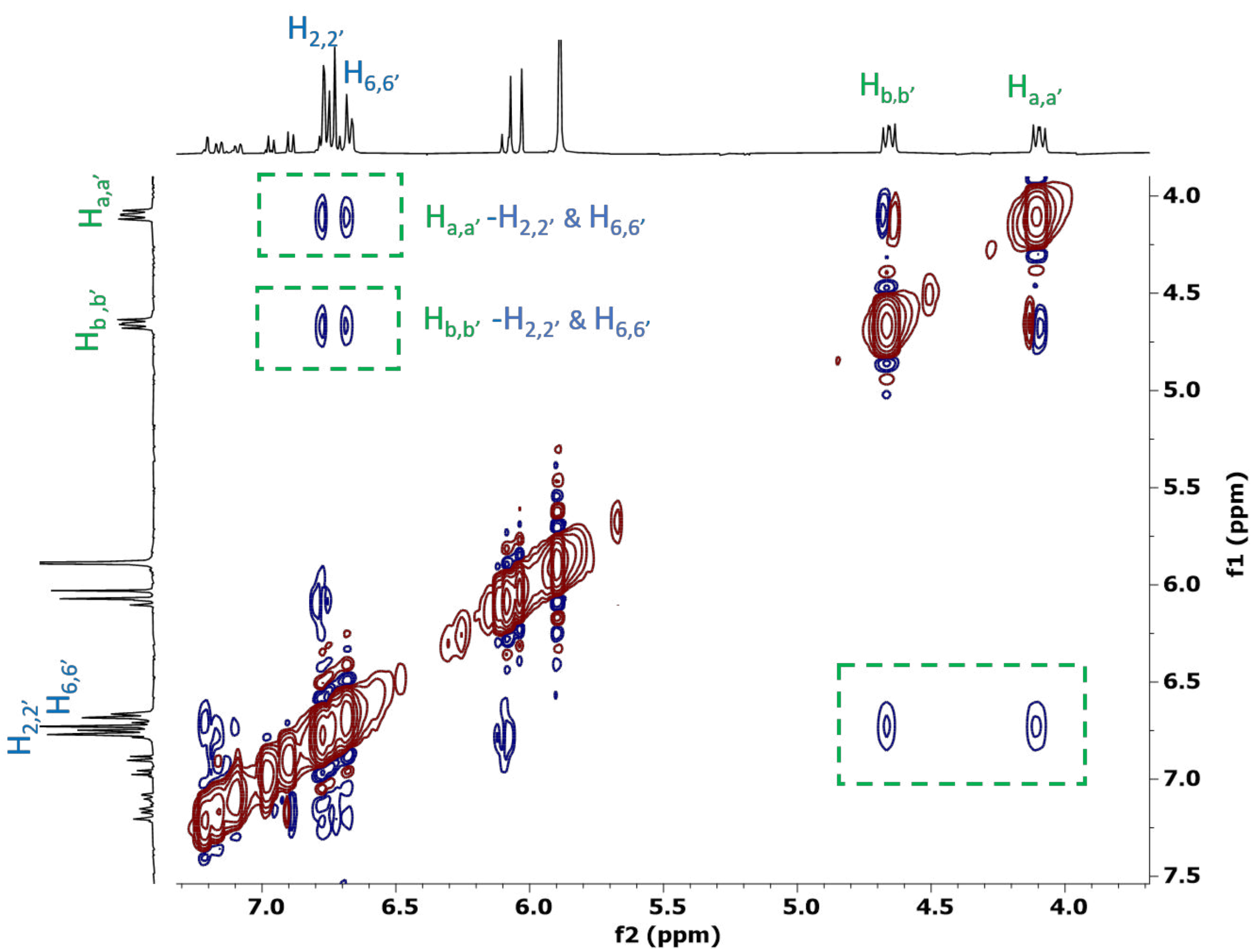

Figure S8. NOESY spectrum of compound **4b** with outlined cross-peaks between hydrogens of cyclobutane and aromatic rings.

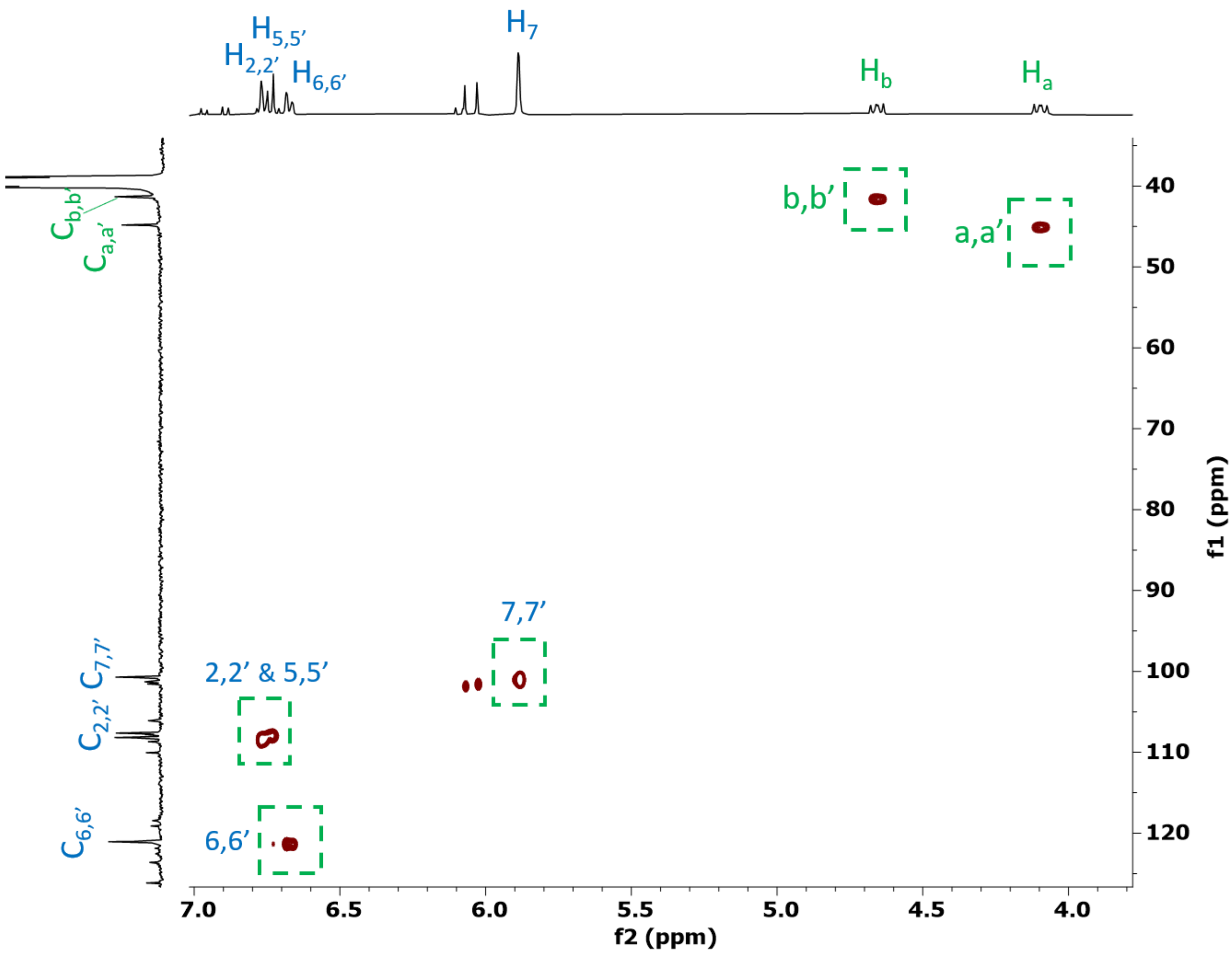

Figure S9. ^{1}H-^{13}C HSQC spectrum of compound **4b** with outlined cross-peaks between hydrogen and carbon atoms of cyclobutane and aromatic rings.

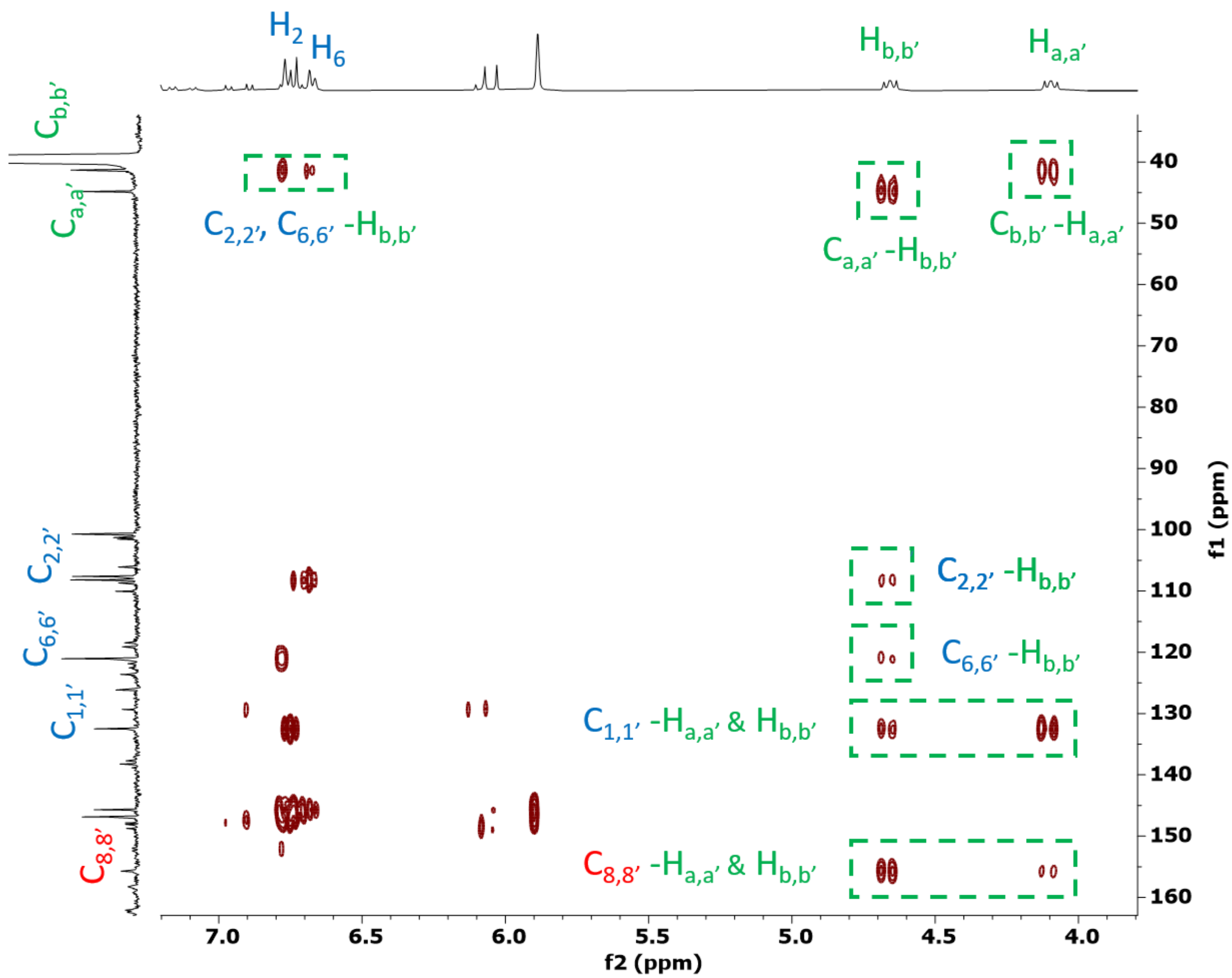

Figure S10. ^{1}H-^{13}C HMBC spectrum of compound **4b** with outlined cross-peaks between atoms of cyclobutane and aromatic rings.

^{1}H NMR spectrum of compound 1a, irr. 3h at 405 nm in DMSO-d$_6$ /CCl$_4$ 1/3, 70% *Z*-isomer

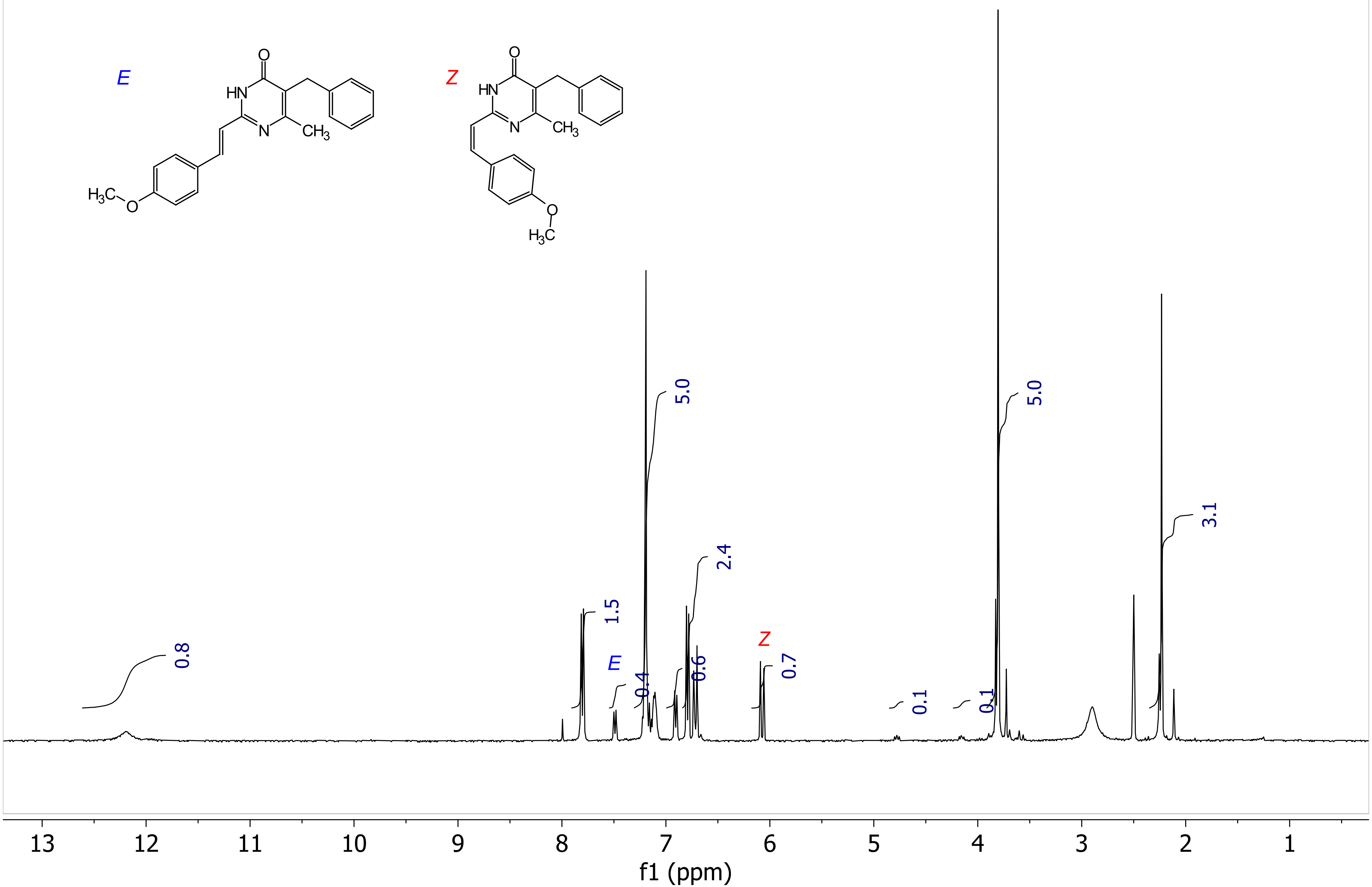

^{1}H NMR spectrum of compound 1a, irr. 10h at 405 nm in DMSO-d$_6$ /CCl$_4$ 1/1, 79% *Z*-isomer

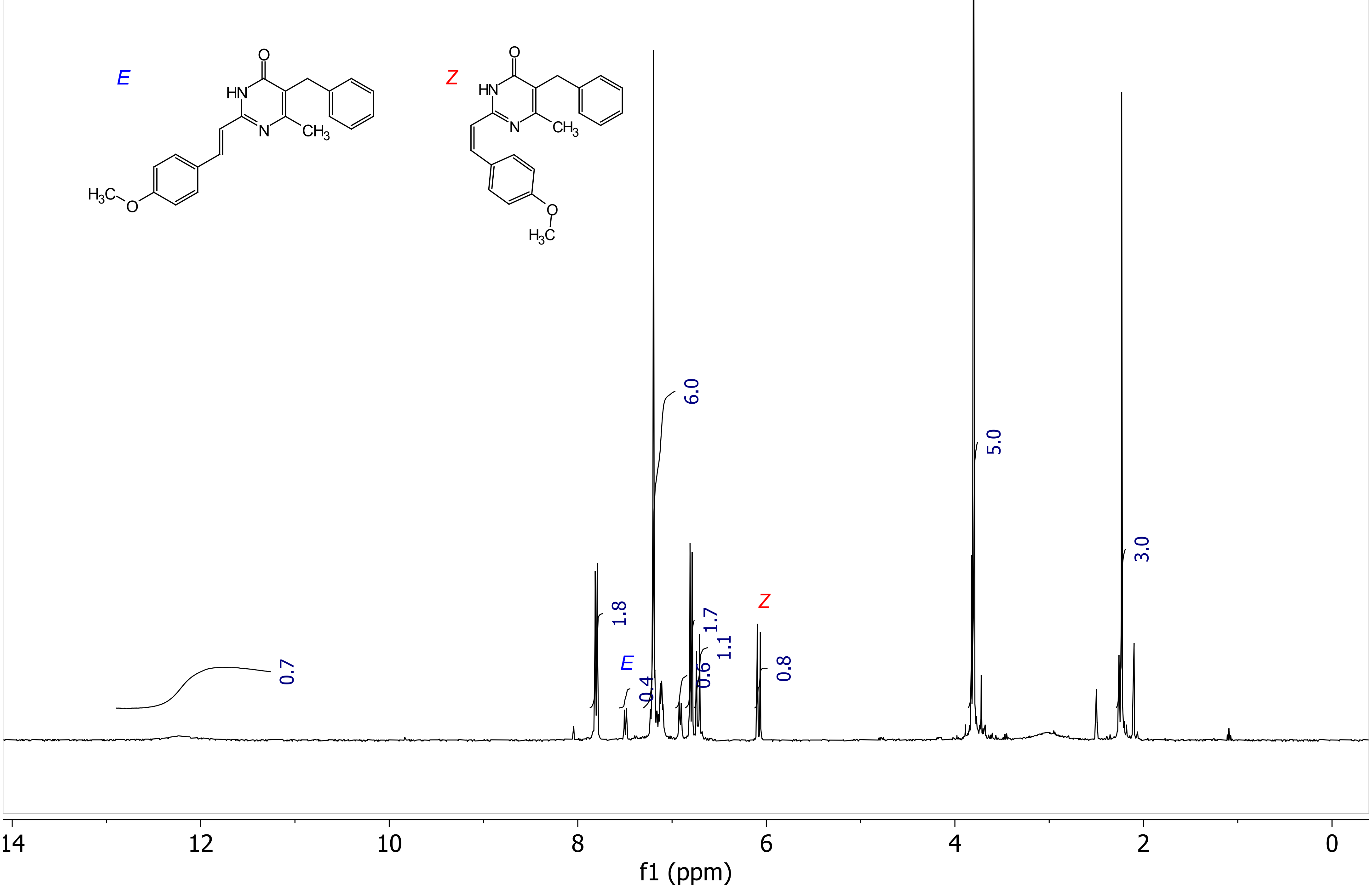

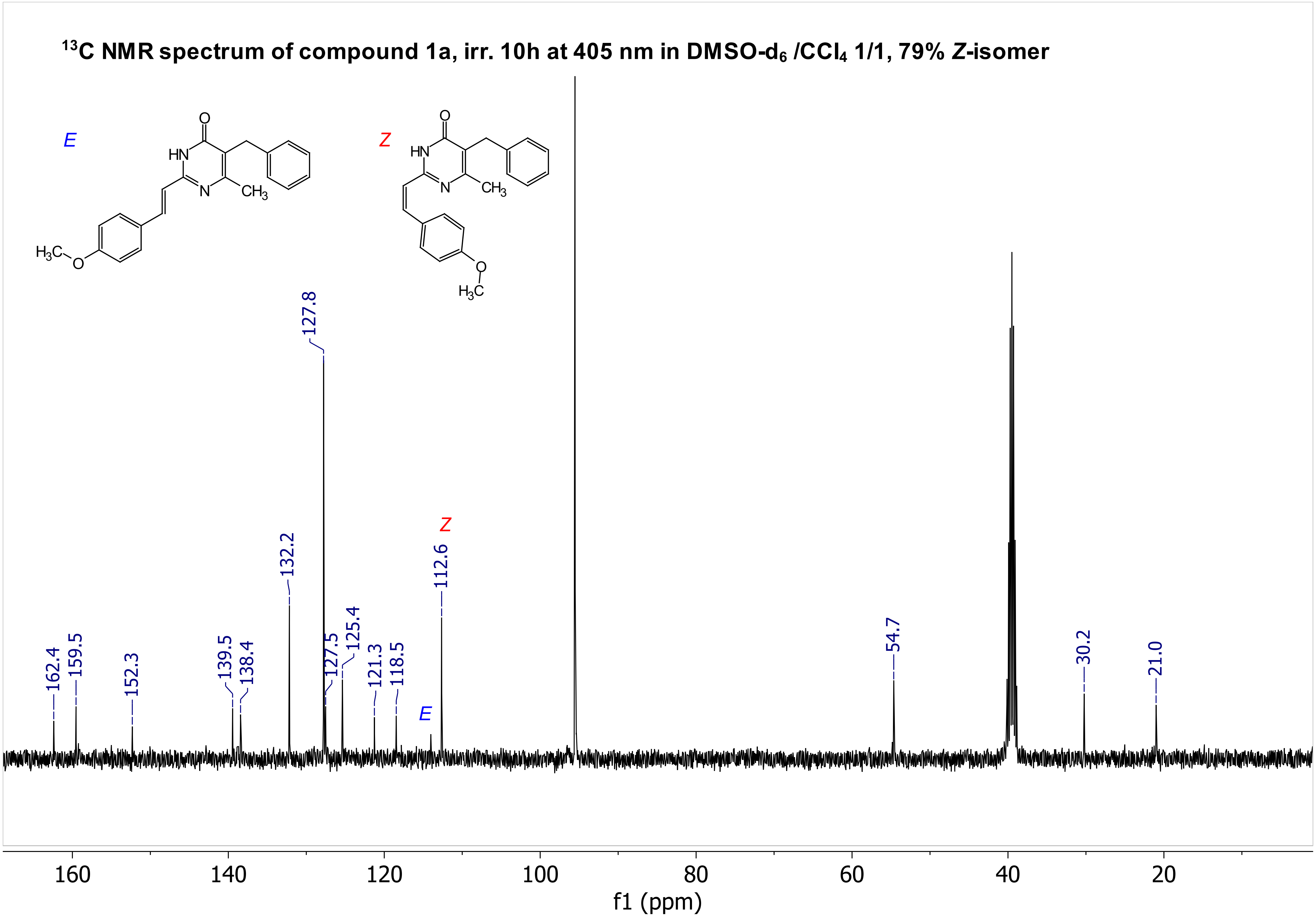

13C NMR spectrum of compound 1a, irr. 10h at 405 nm in DMSO-d6 /CCl4 1/1, 79% Z-isomer
E
Z
HN
N
O
CH3
H3C
O
162.4
159.5
152.3
139.5
138.4
132.2
127.8
127.5
125.4
121.3
118.5
112.6
54.7
30.2
21.0
160
140
120
100
80
60
40
20
f1 (ppm)

^1H-^{13}C HSQC spectrum of compound 1a, irr. 10h at 405 nm in DMSO-d_6 /CCl_4 1/1, 79% *Z*-isomer

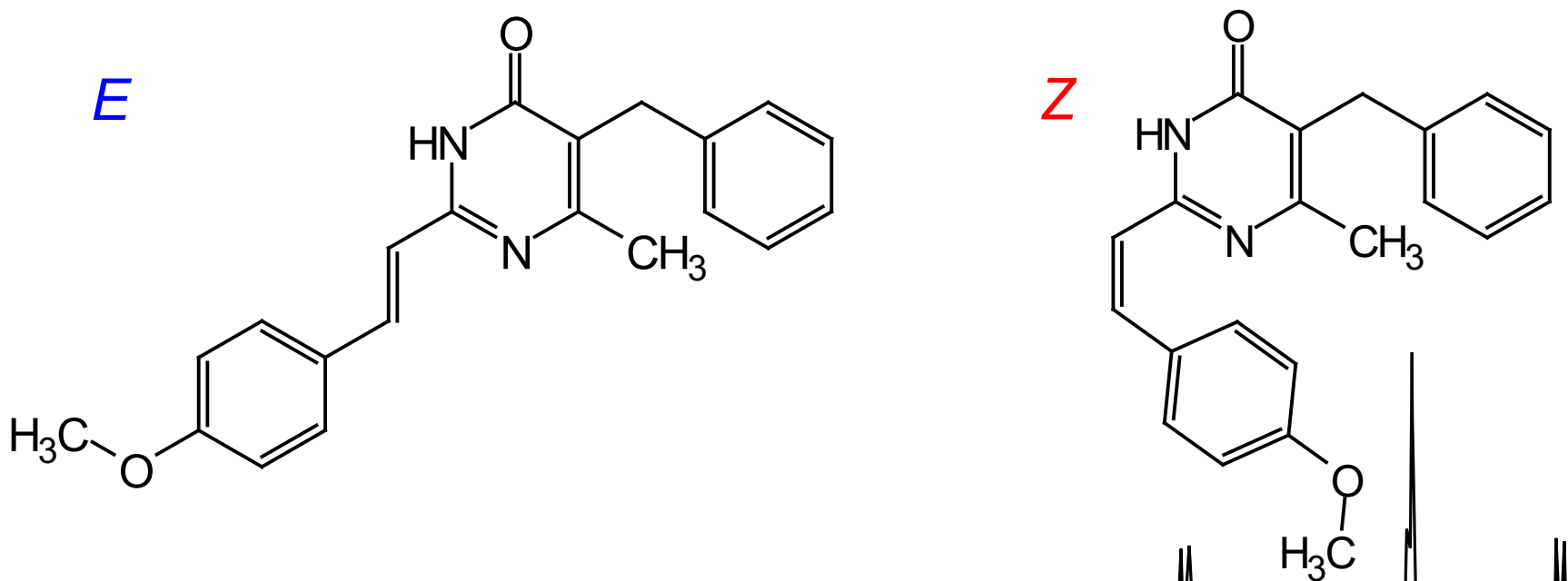

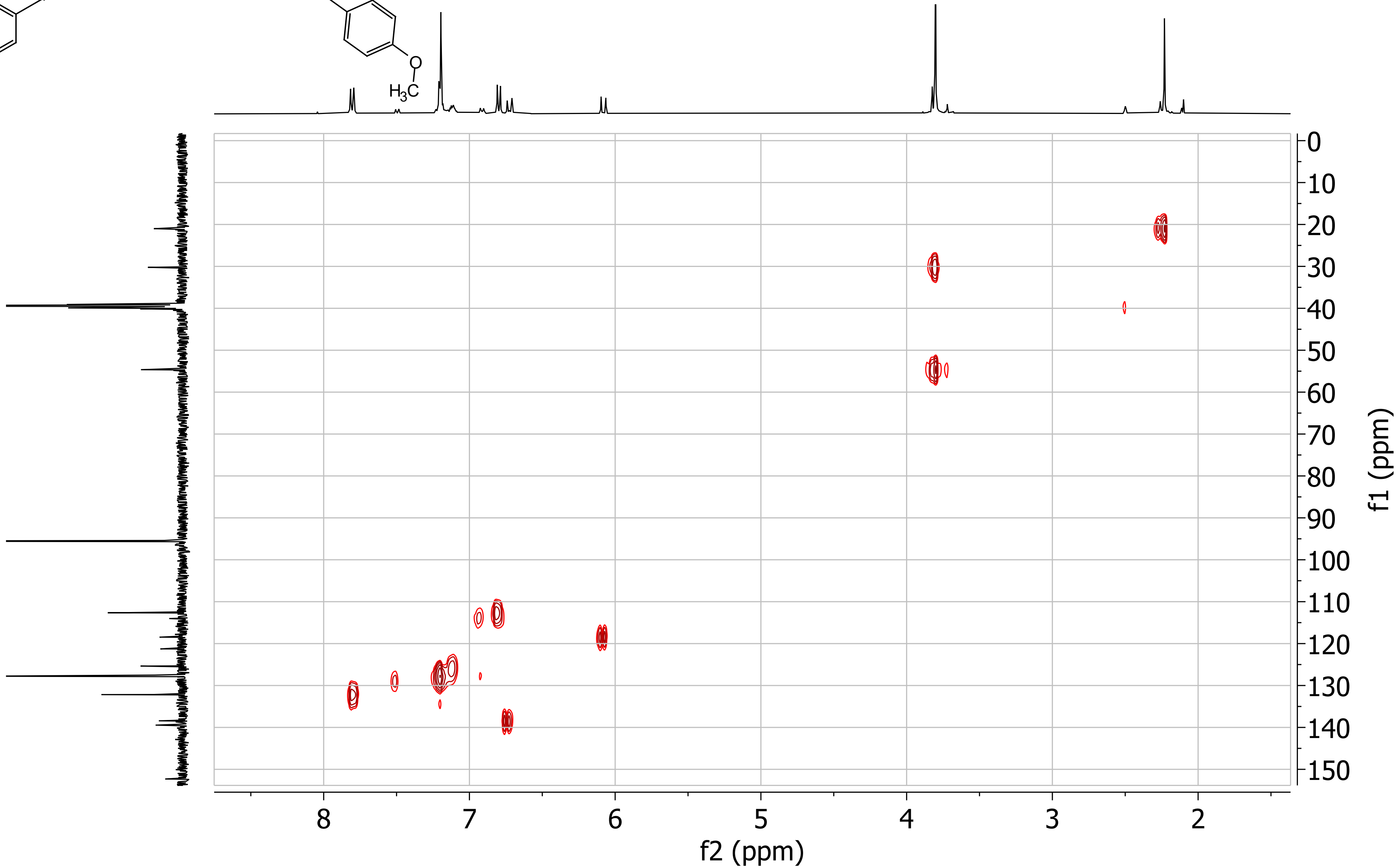

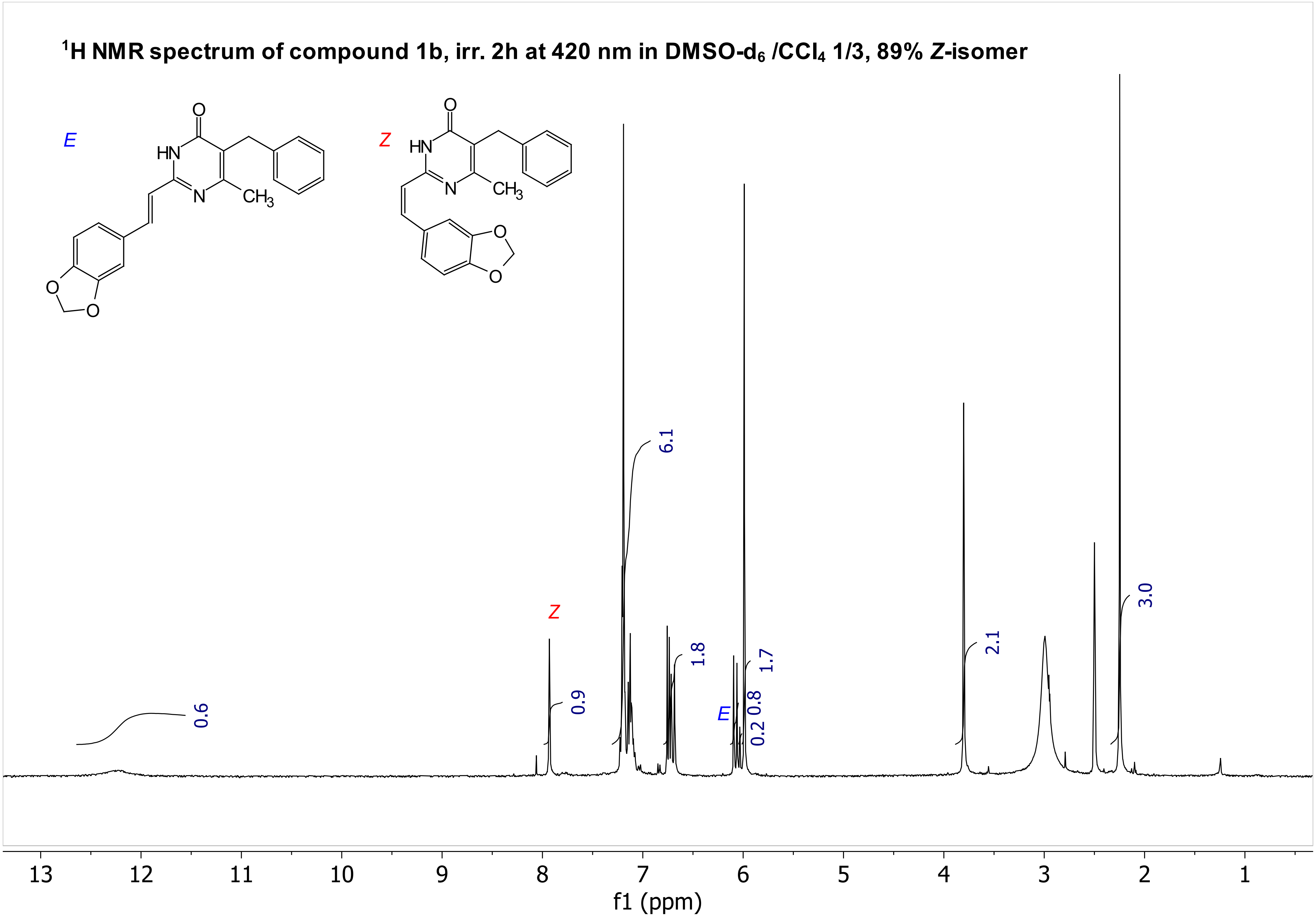

1H NMR spectrum of compound 1b, irr. 2h at 420 nm in DMSO-d6 /CCl4 1/3, 89% Z-isomer
E
Z
HN
O
N
CH3
O
O
Z
E
0.6
0.9
6.1
1.8
0.2
0.8
1.7
2.1
3.0
13
12
11
10
9
8
7
6
5
4
3
2
1
f1 (ppm)

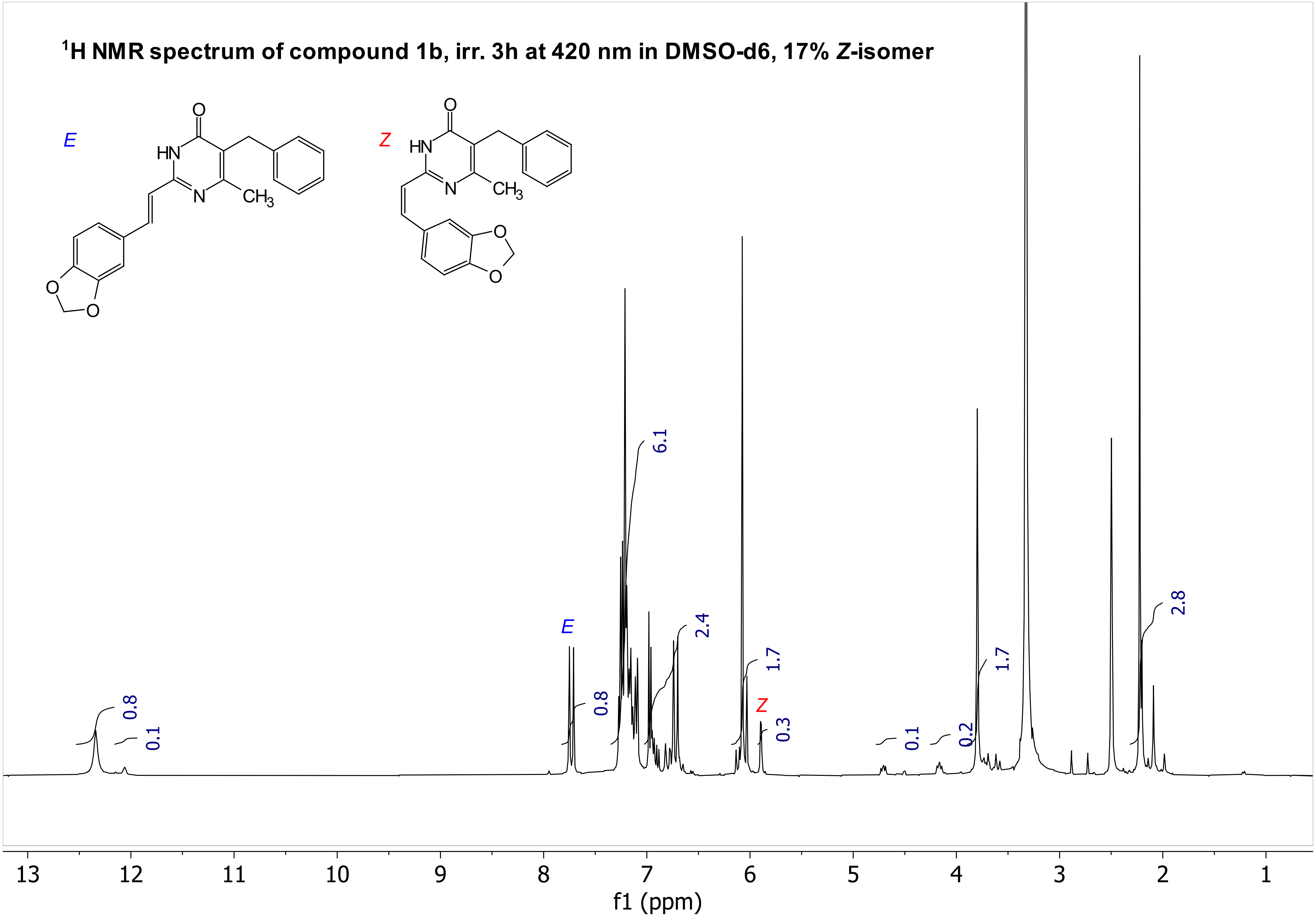

1H NMR spectrum of compound 1b, irr. 3h at 420 nm in DMSO-d6, 17% Z-isomer
E
Z
E
Z
0.8
0.1
0.8
6.1
2.4
1.7
0.3
0.1
0.2
1.7
2.8
13
12
11
10
9
8
7
6
5
4
3
2
1
f1 (ppm)

^{1}H NMR spectrum of compound 1c, irr. 3h at 420 nm in DMSO-d_6 /CCl_4 1/1, 35% *Z*-isomer

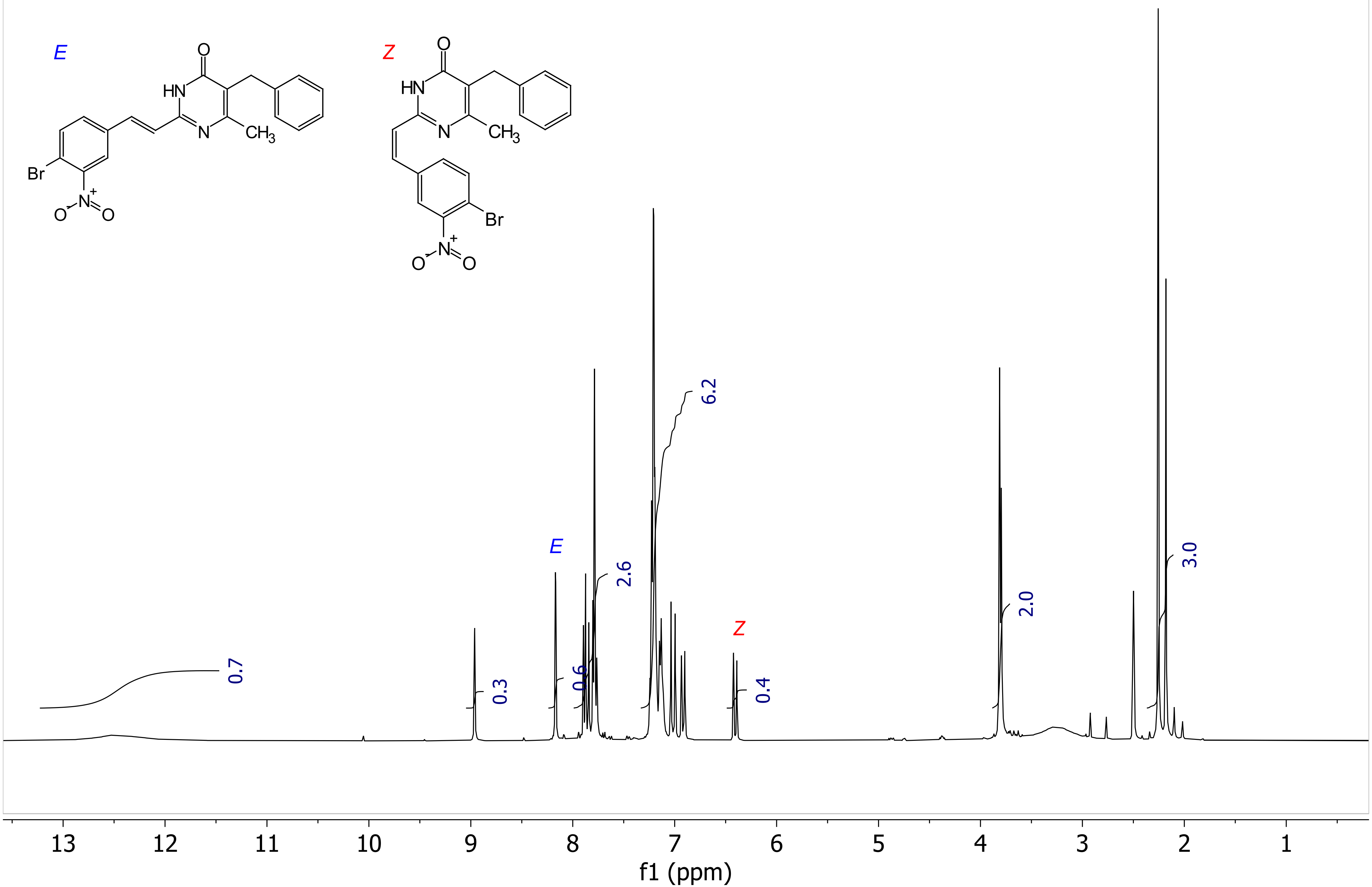

^{1}H NMR spectrum of compound 1d, irr. 2h at 450 nm in DMSO-d6, 40% *Z*-isomer

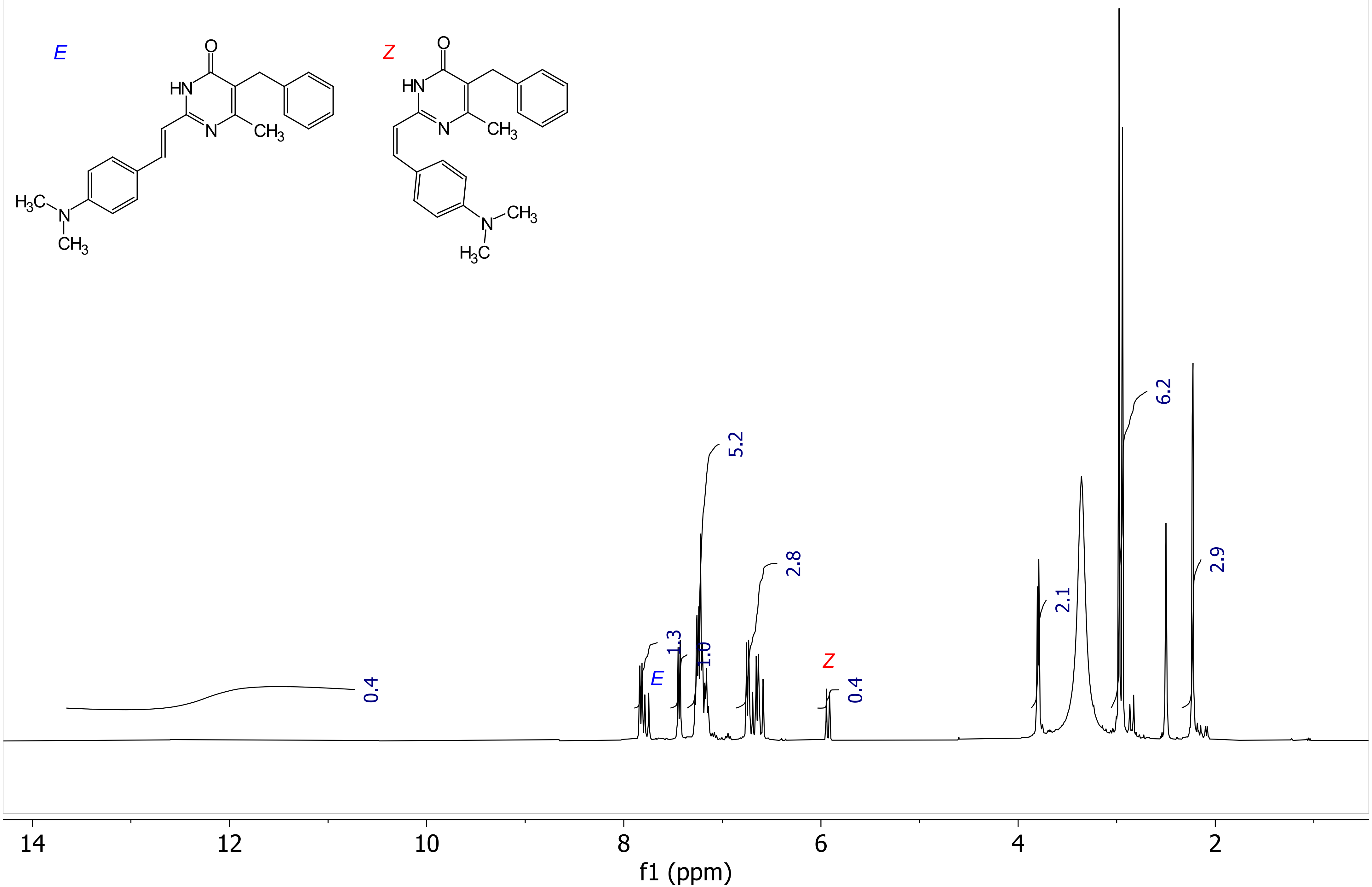

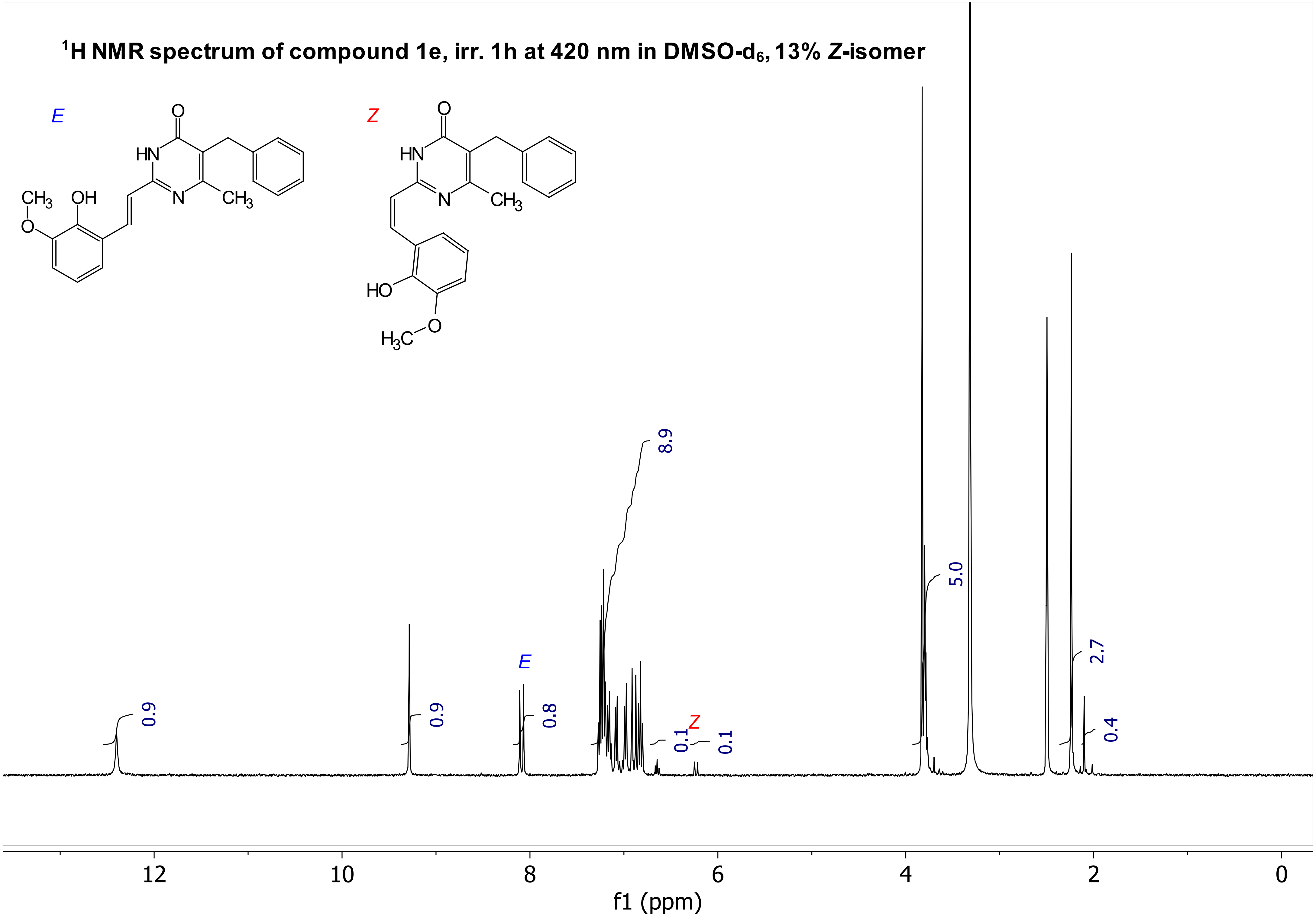

^{1}H NMR spectrum of compound 1e, irr. 1h at 420 nm in DMSO-d$_6$, 13% *Z*-isomer
E
Z
HN
O
N
CH3
CH3
OH
O
HO
H3C
O
0.9
0.9
0.8
8.9
0.1
0.1
5.0
2.7
0.4
12
10
8
6
4
2
0
f1 (ppm)

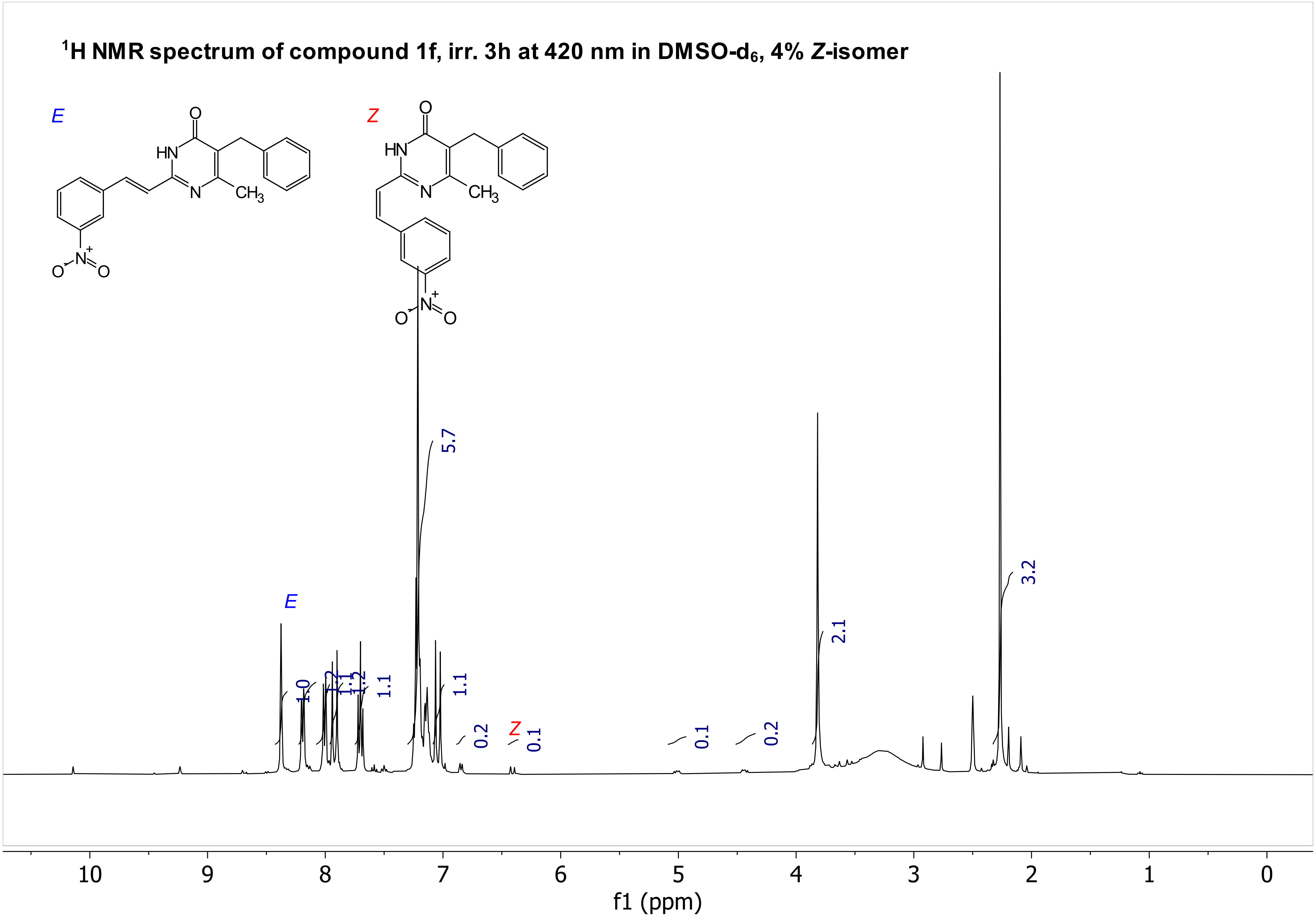

^{1}H NMR spectrum of compound 1f, irr. 3h at 420 nm in DMSO-d6, 4% Z-isomer
E
Z
HN
N
CH3
O
5.7
3.2
2.1
1.0
1.2
1.1
1.2
1.1
1.1
0.2
0.1
0.1
0.2
10
9
8
7
6
5
4
3
2
1
0
f1 (ppm)

^{1}H NMR spectrum of compound 1g, irr. 2h at 420 nm in DMSO-d_6/CCl4 1/3, 10% *Z*-isomer

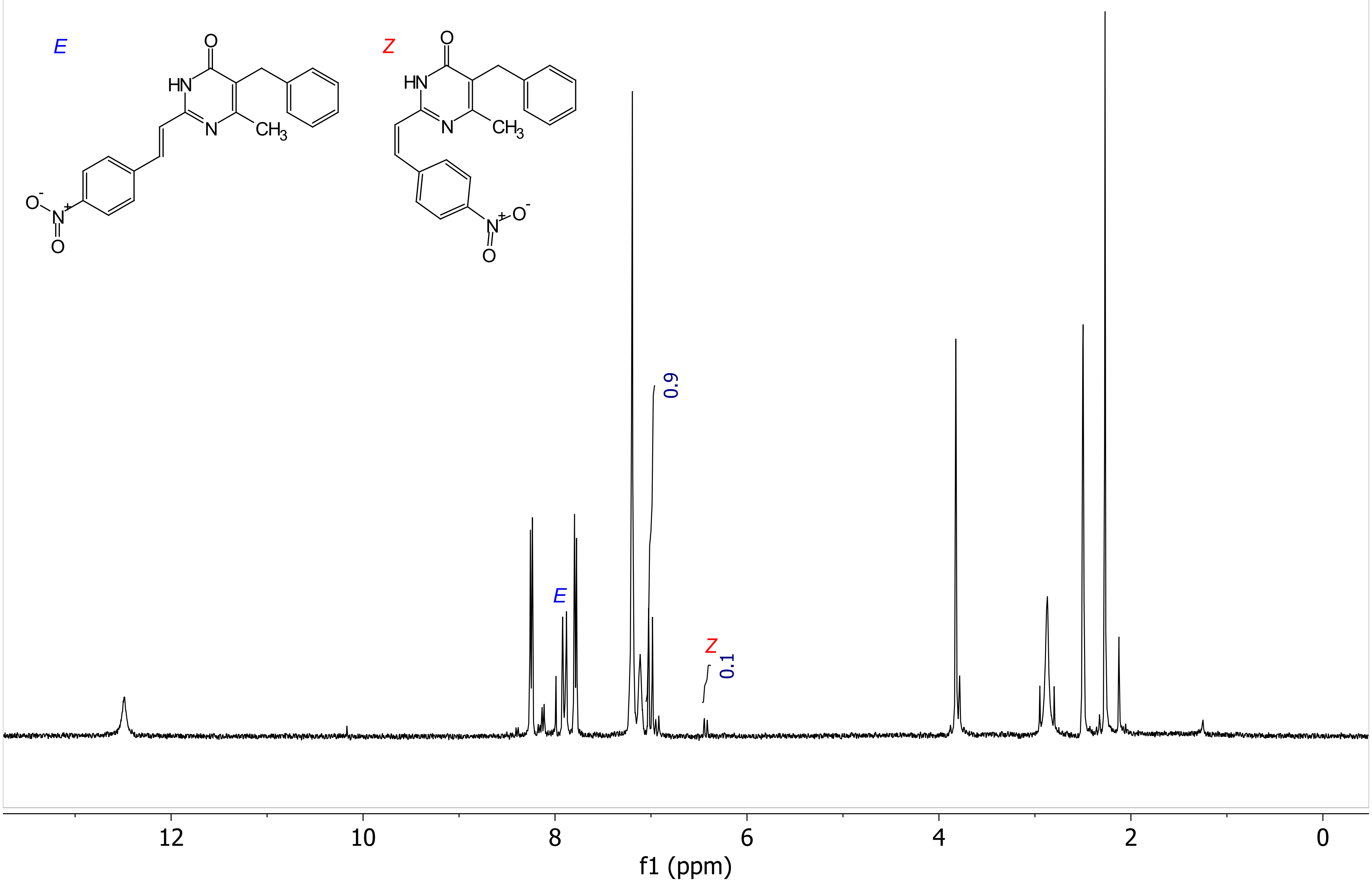

^{1}H NMR spectrum of compound 1h, irr. 2h at 365 nm in DMSO-d_6/CCl_4 1/3, 6% *Z*-isomer

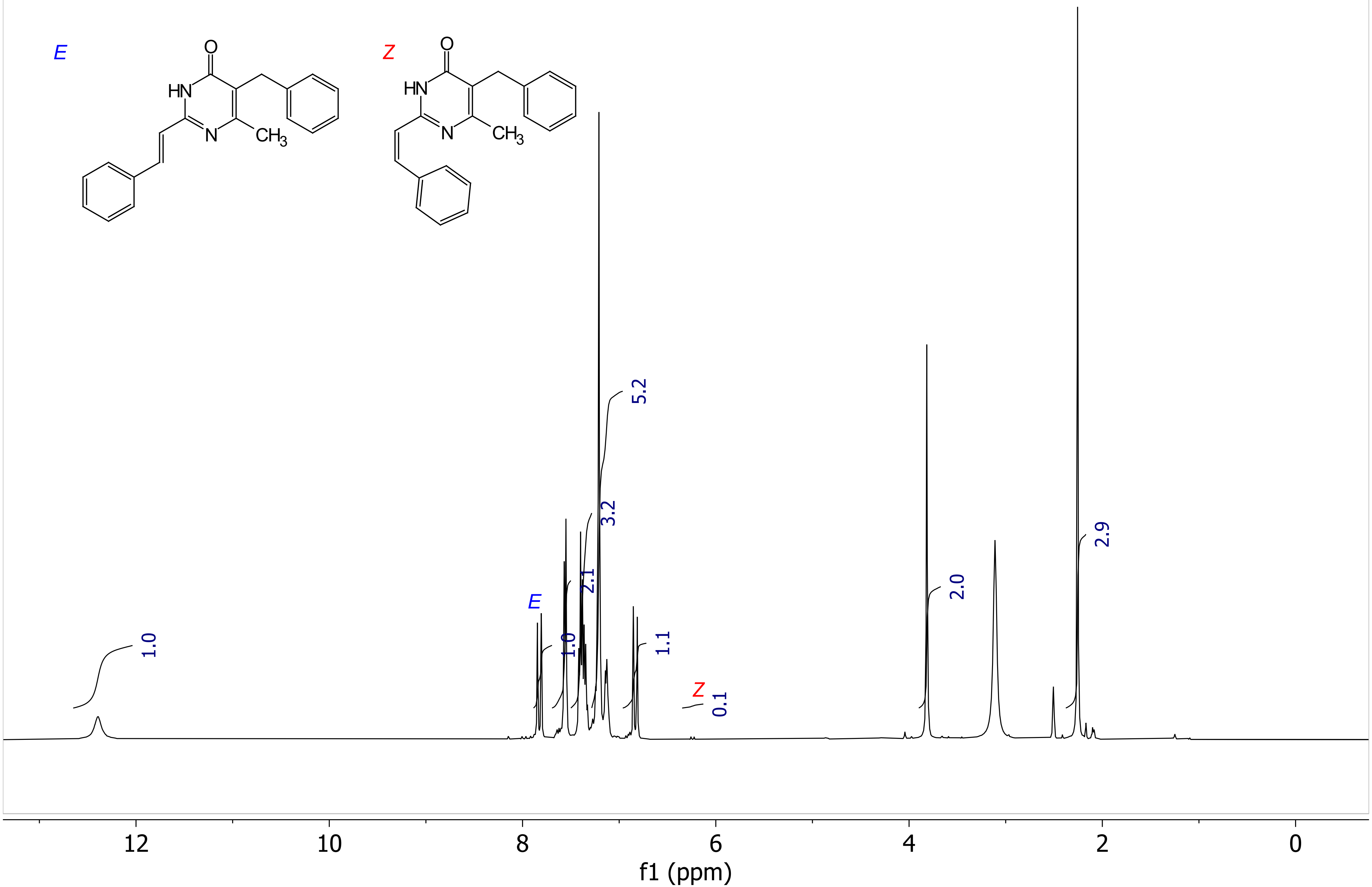

^{1}H NMR spectrum of compound 2a, irr. 10h at 420 nm in DMSO-d_6/CCl_4 1/3, 75% *Z*-isomer

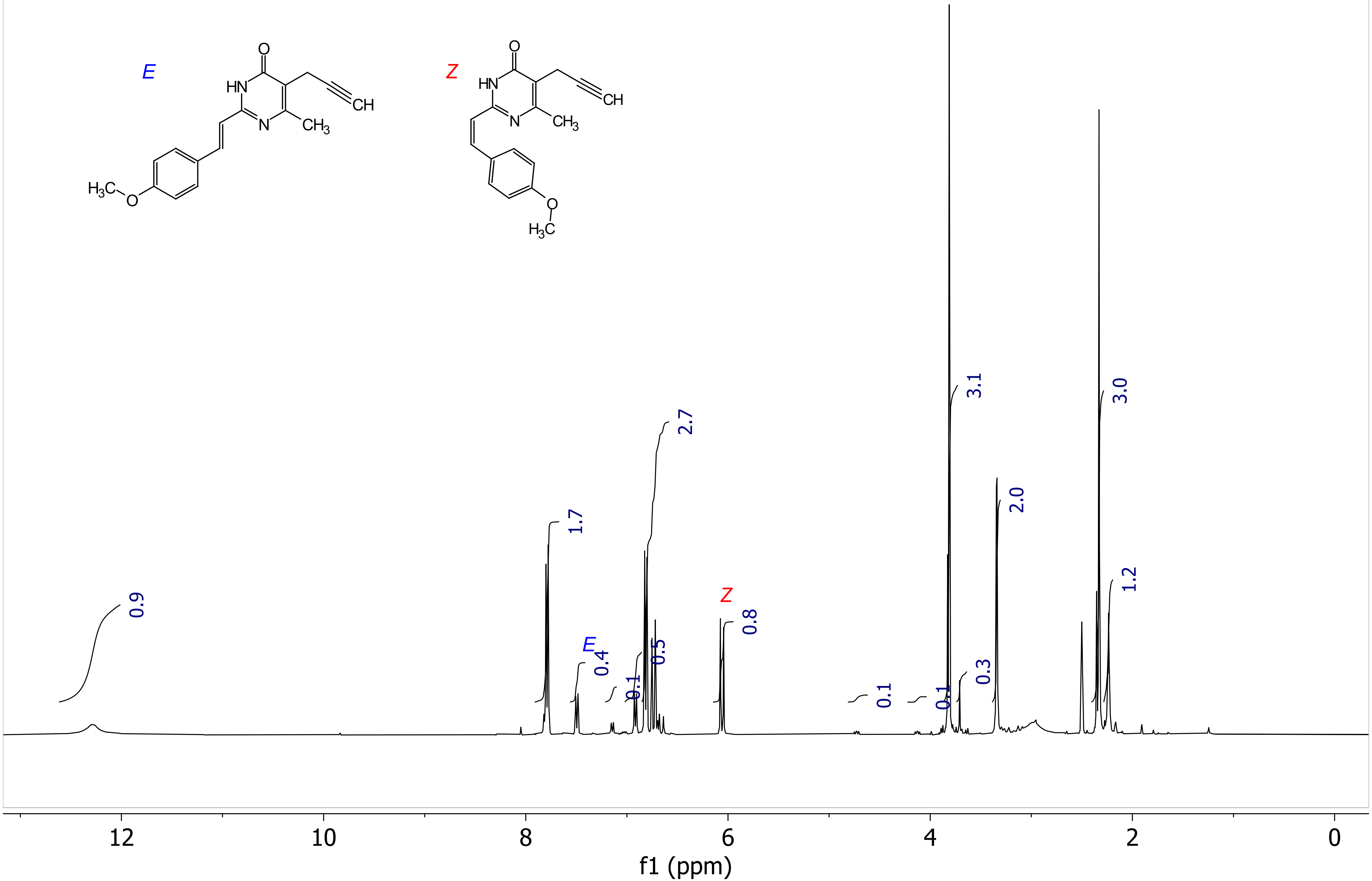

^{13}C NMR spectrum of compound 2a, irr. 10h at 420 nm in DMSO-d_6/CCl_4 1/3, 75% *Z*-isomer

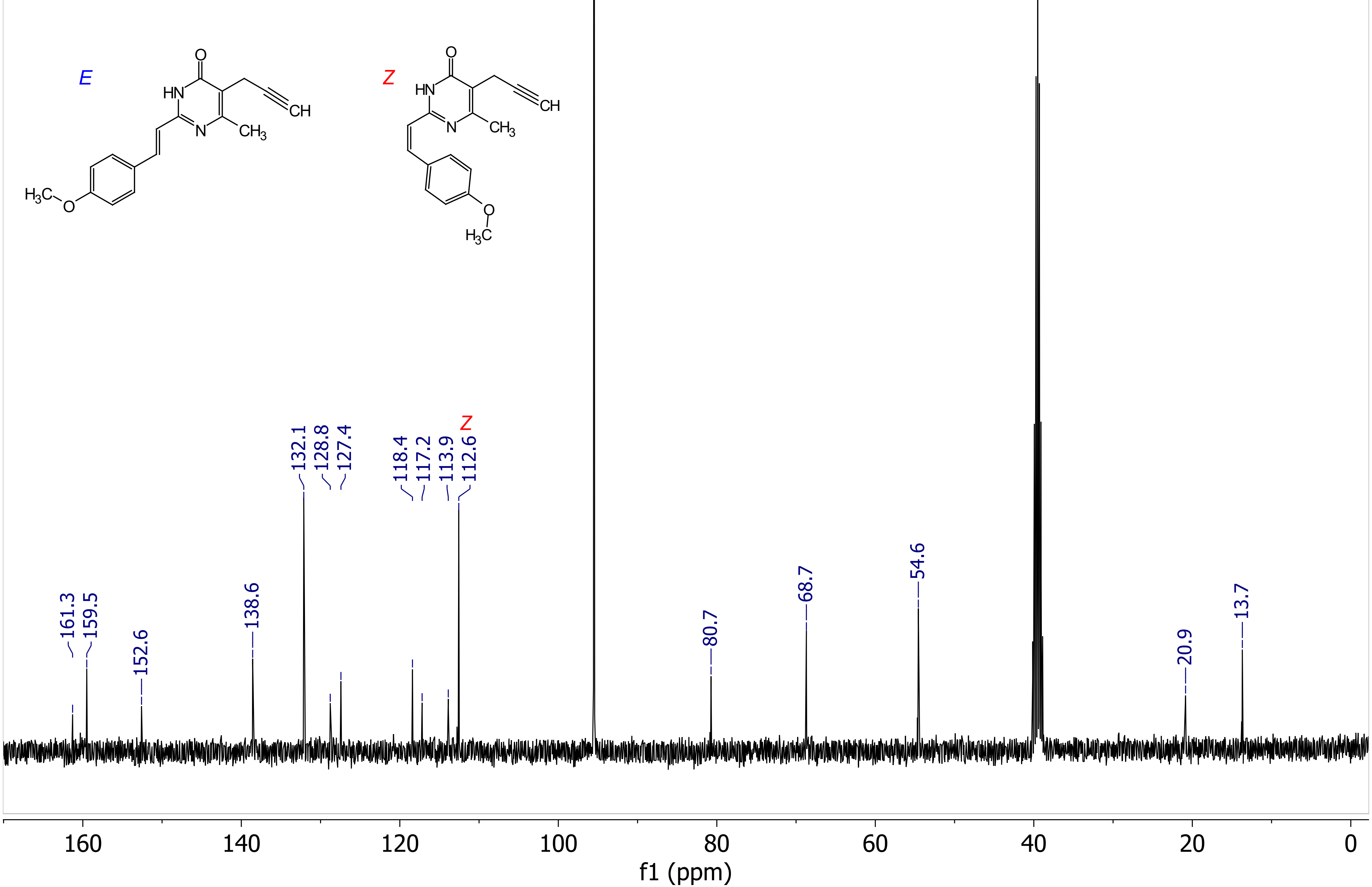

^{1}H-^{13}C HMBC spectrum of compound 2a, irr. 10h at 420 nm in DMSO-d_6/CCl_4 1/3, 75% *Z*-isomer

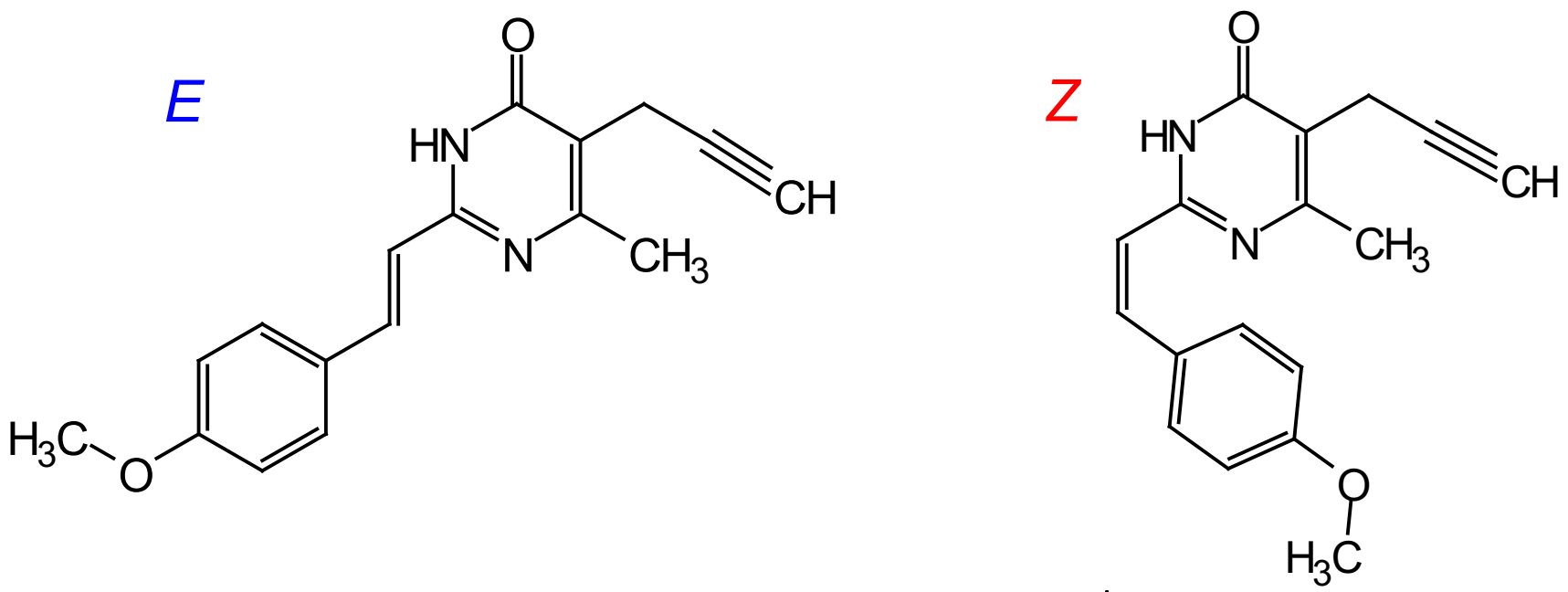

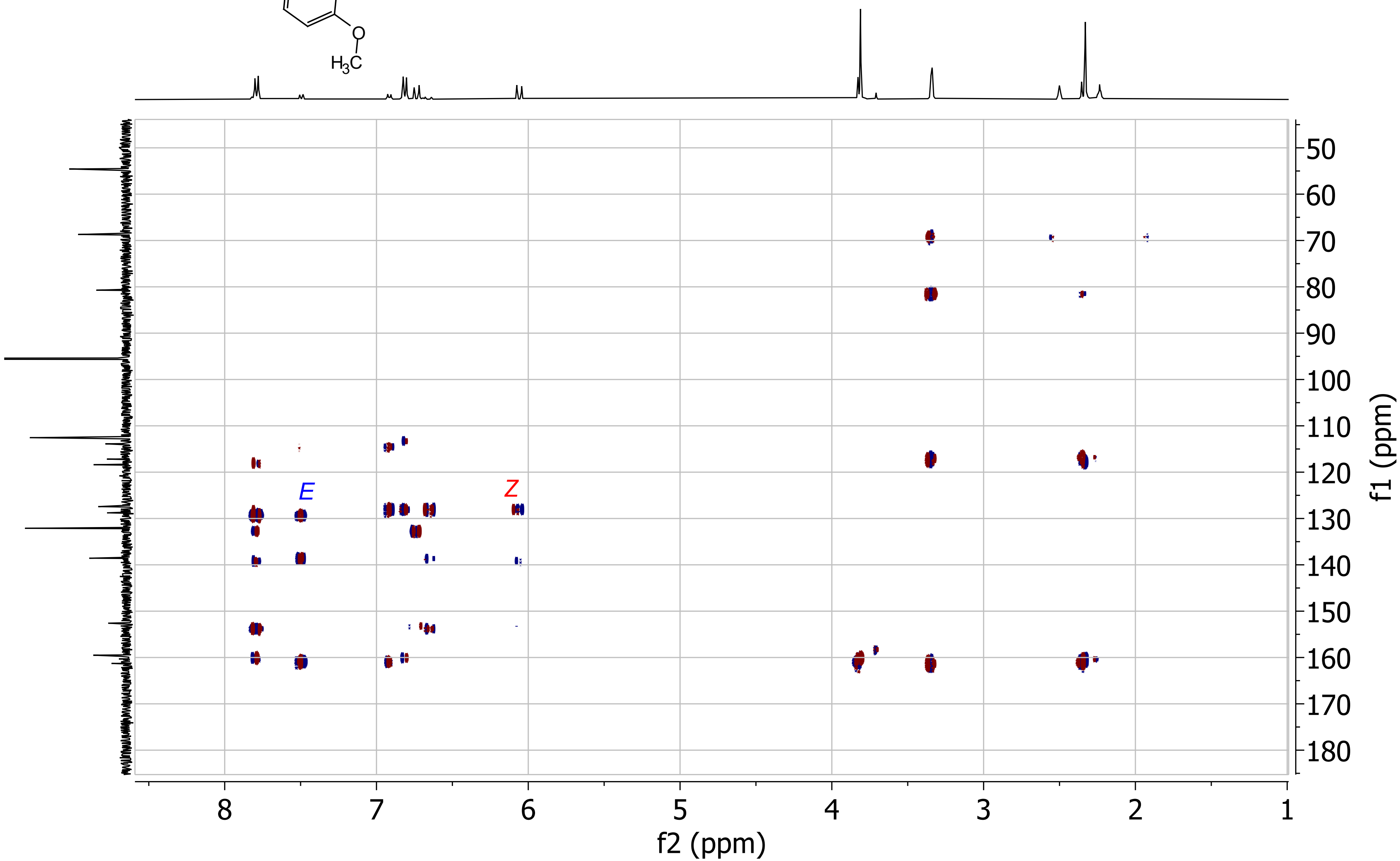

^{1}H-^{13}C HSQC spectrum of compound 2a, irr. 10h at 420 nm in DMSO-d_6/CCl_4 1/3, 75% *Z*-isomer

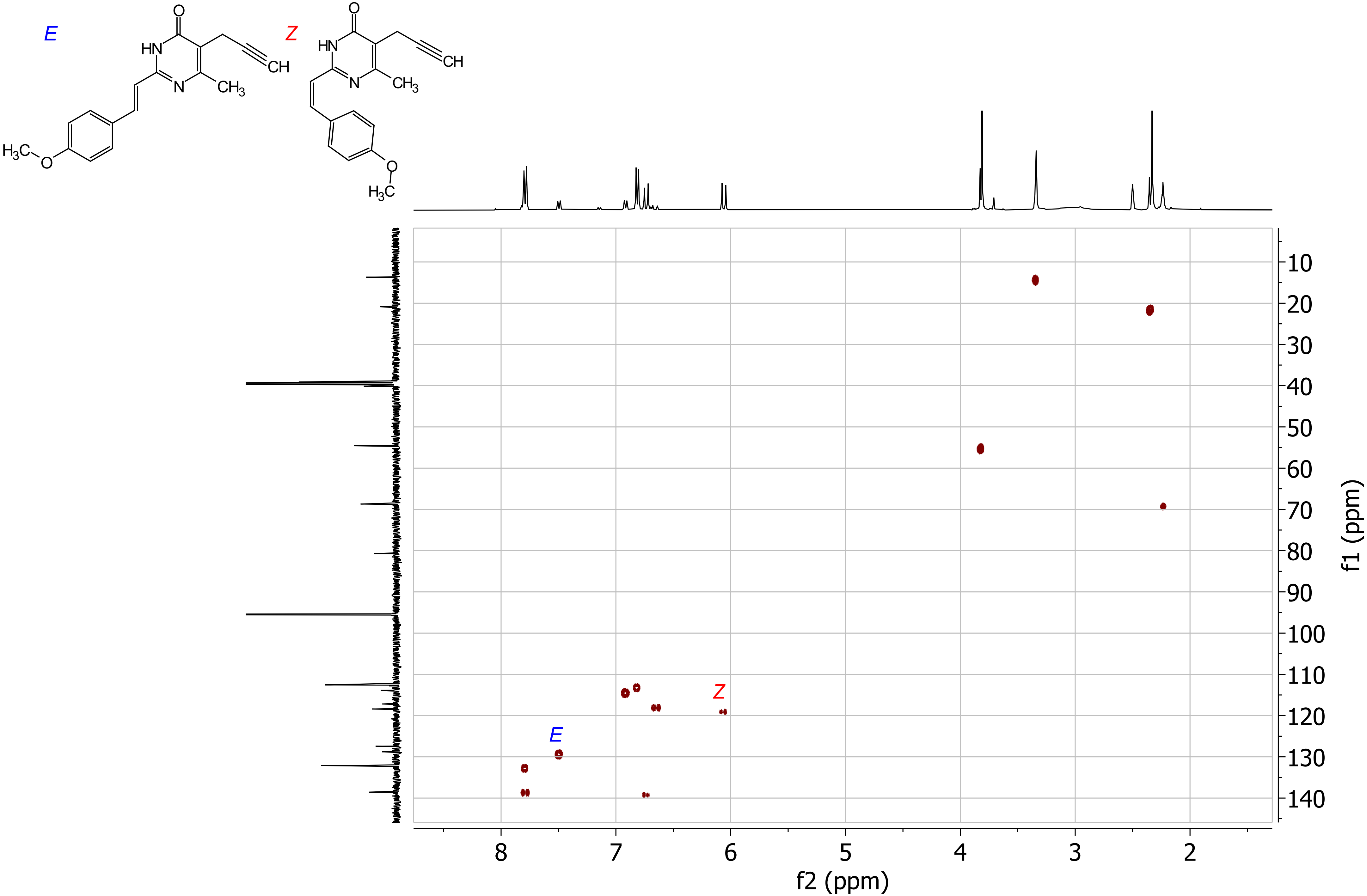

NOESY spectrum of compound 2a, irr. 10h at 420 nm in DMSO-d$_6$/CCl$_4$ 1/3, 75% *Z*-isomer

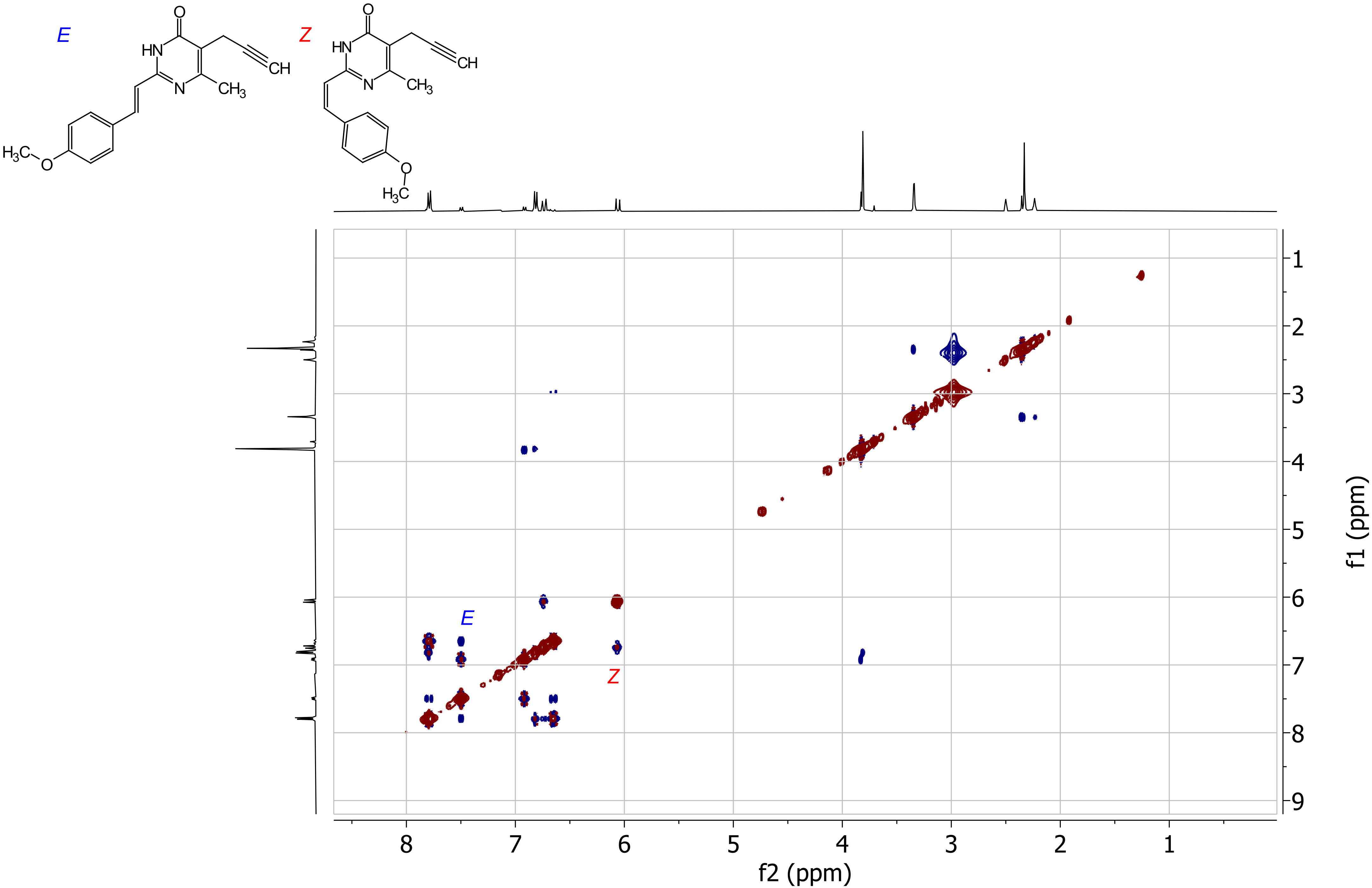

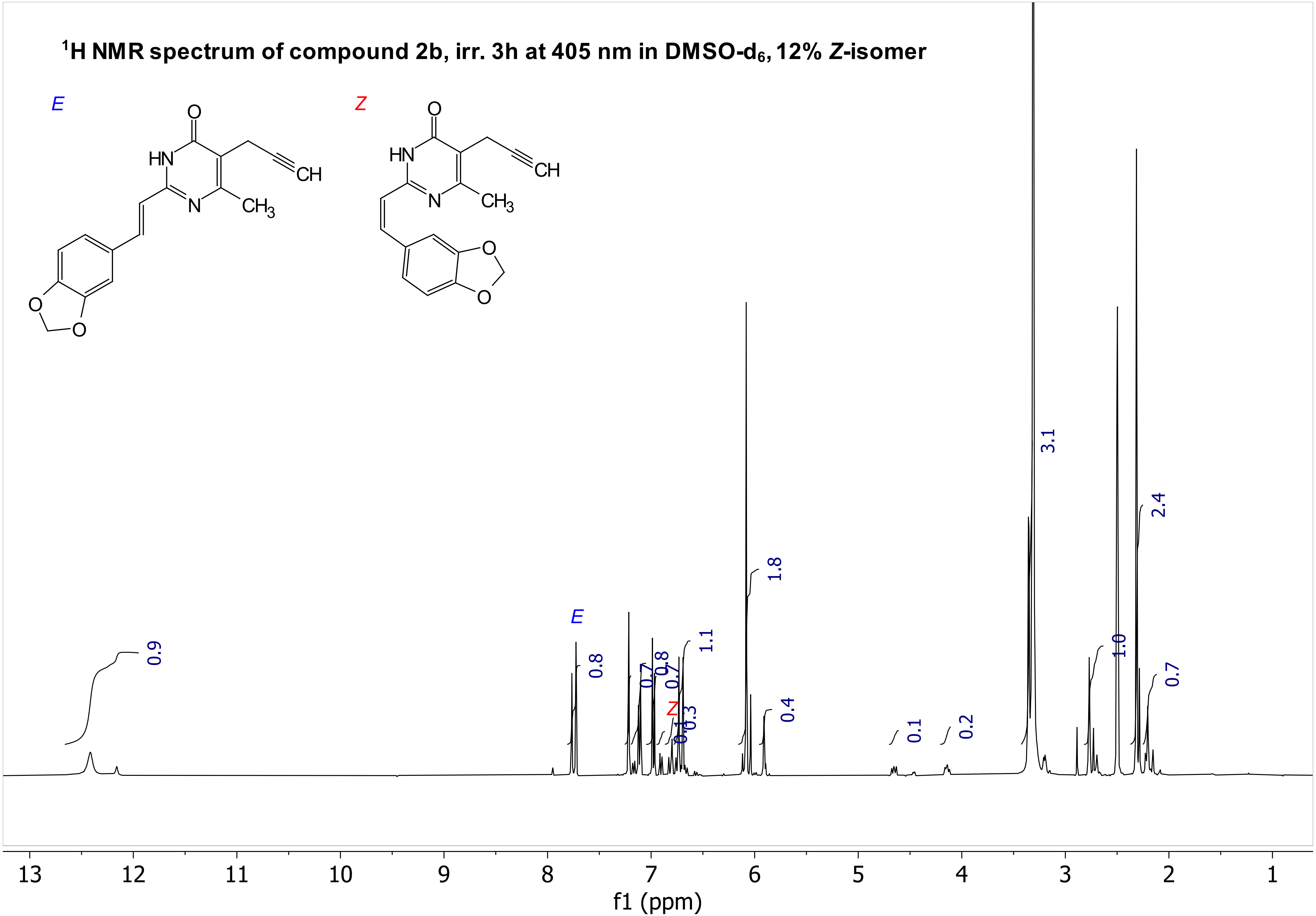

¹H NMR spectrum of compound 2b, irr. 3h at 405 nm in DMSO-d6, 12% Z-isomer
E
Z
O
HN
N
CH
CH3
O
O
E
Z
0.9
0.8
0.7
0.8
0.7
1.1
0.3
1.8
0.4
0.1
0.2
3.1
1.0
2.4
0.7
13
12
11
10
9
8
7
6
5
4
3
2
1
f1 (ppm)

^{1}H NMR spectrum of compound 2b, irr. 1h at 450 nm in DMSO-d_6/CCl_4 1/3, 89% *Z*-isomer

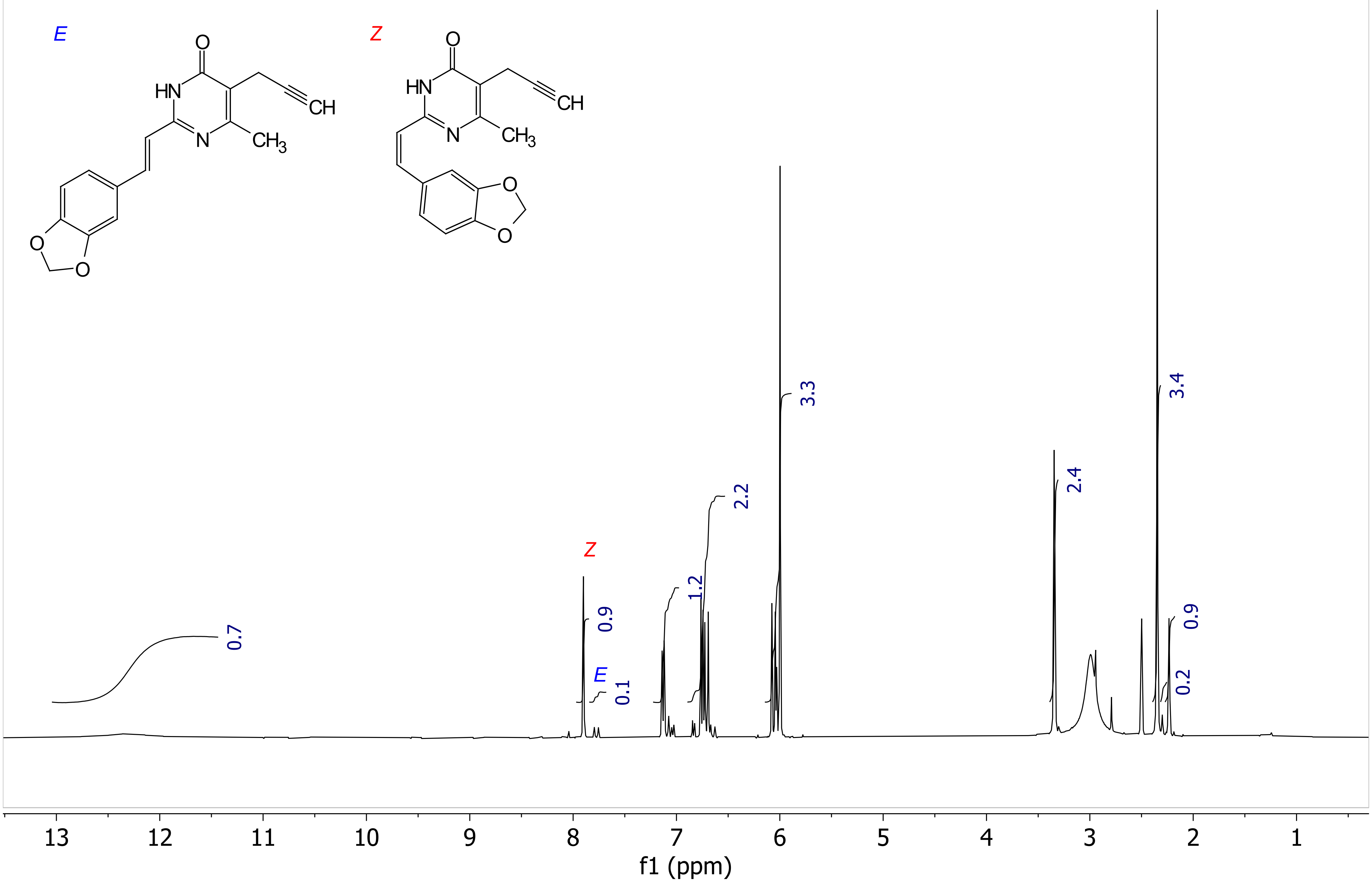

^{13}C NMR spectrum of compound 2b, irr. 1h at 450 nm in DMSO-d_6/CCl_4 1/3, 89% *Z*-isomer

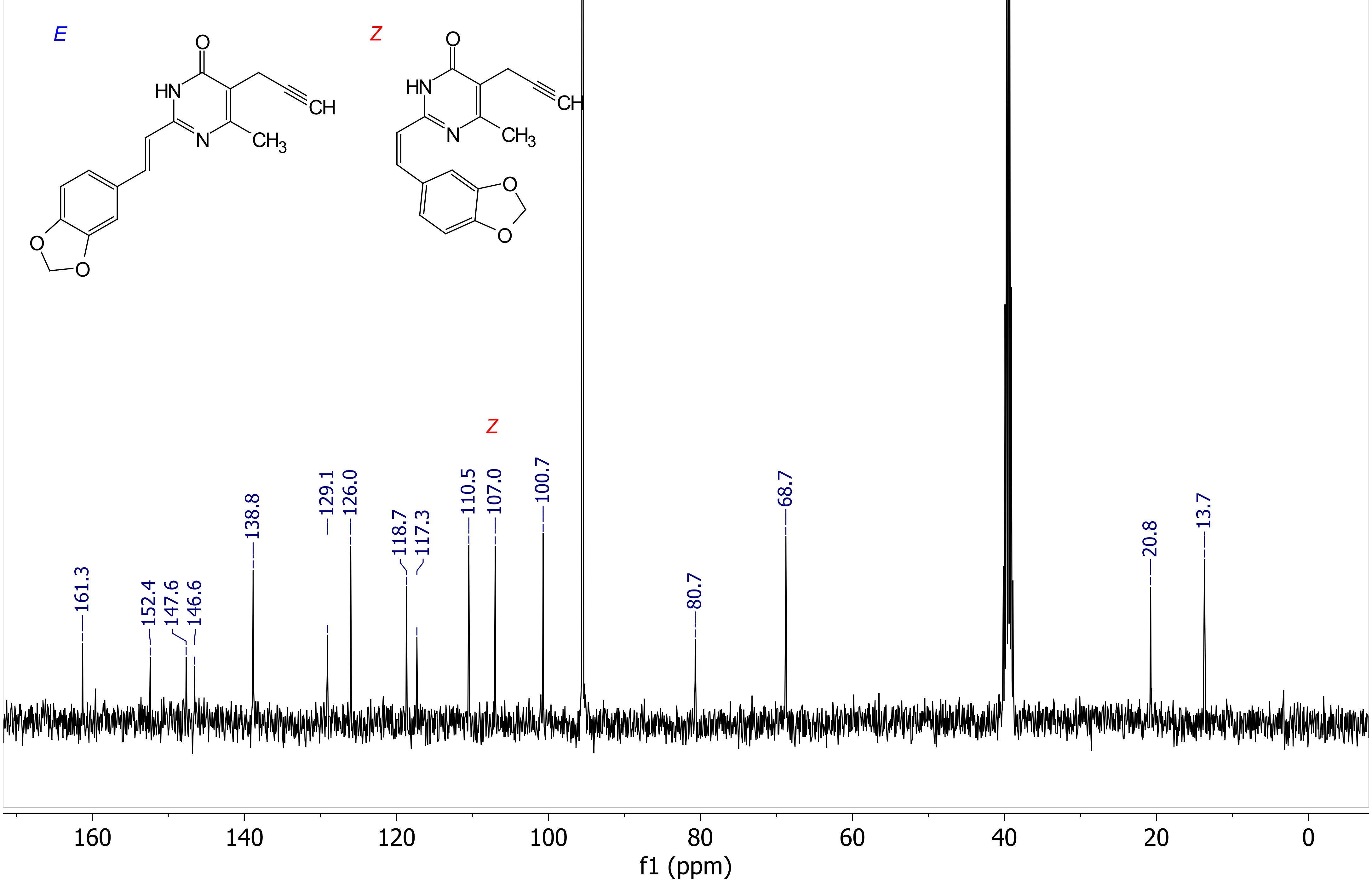

^{1}H NMR spectrum of compound 2c, irr. 2h at 420 nm in DMSO-d_6/CCl_4 1/1, 77% *Z*-isomer

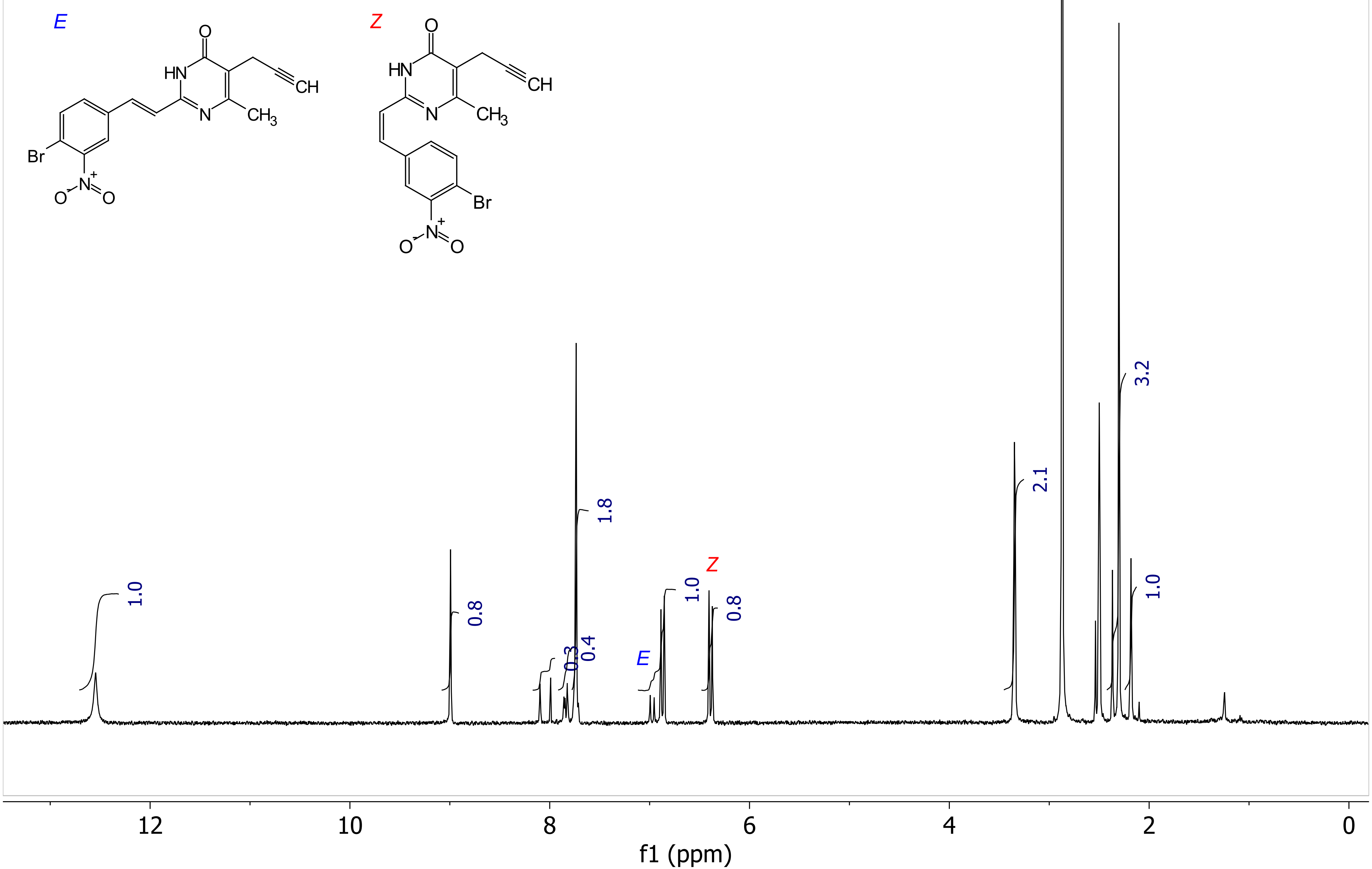

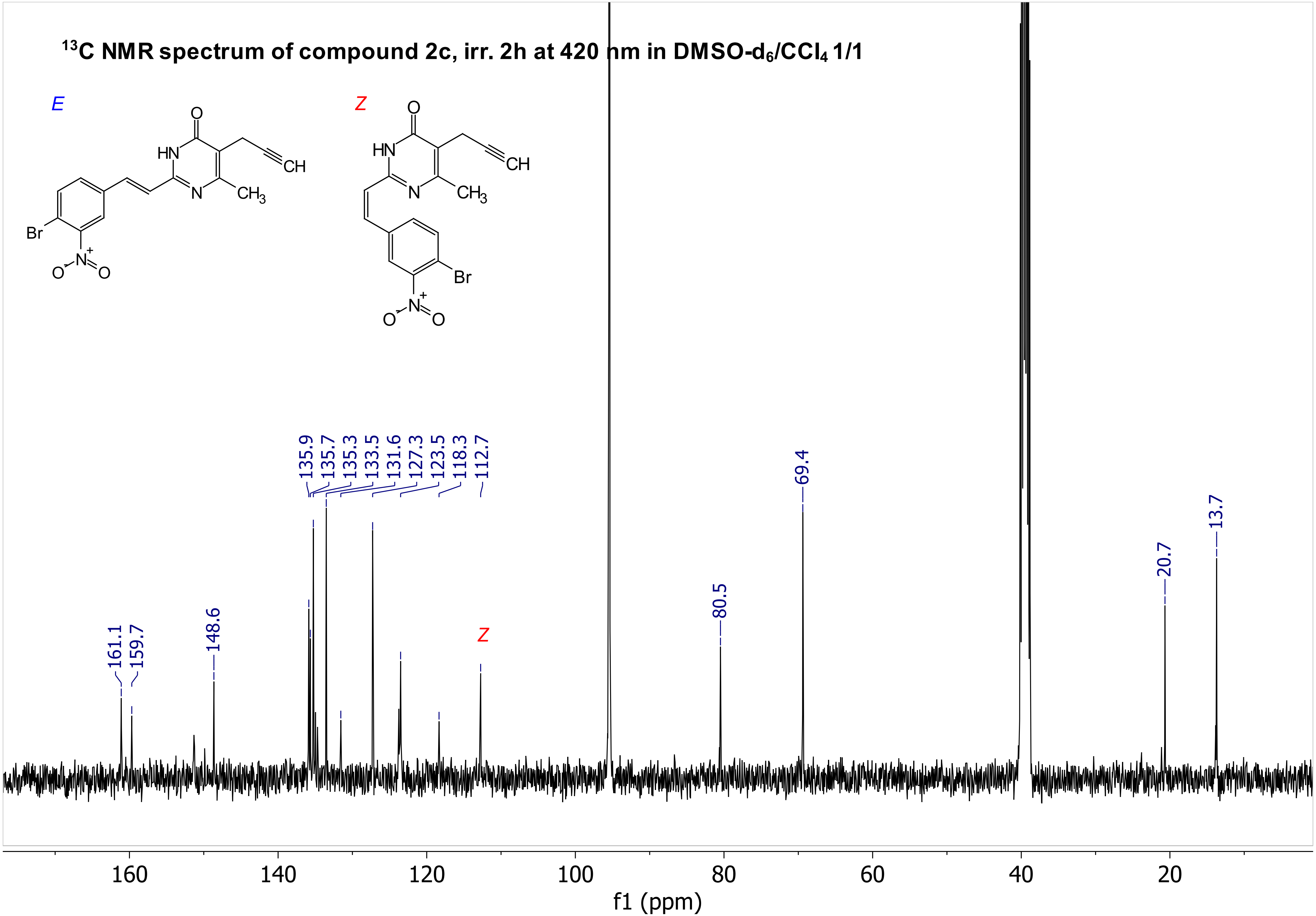

13C NMR spectrum of compound 2c, irr. 2h at 420 nm in DMSO-d6/CCl4 1/1
E
Z
O
HN
N
CH
CH3
Br
N+
O-
O
161.1
159.7
148.6
135.9
135.7
135.3
133.5
131.6
127.3
123.5
118.3
112.7
Z
80.5
69.4
20.7
13.7
160
140
120
100
80
60
40
20
f1 (ppm)

^{1}H NMR spectrum of compound 2d, irr. 2h at 420 nm in DMSO-d_6, 40% *Z*-isomer

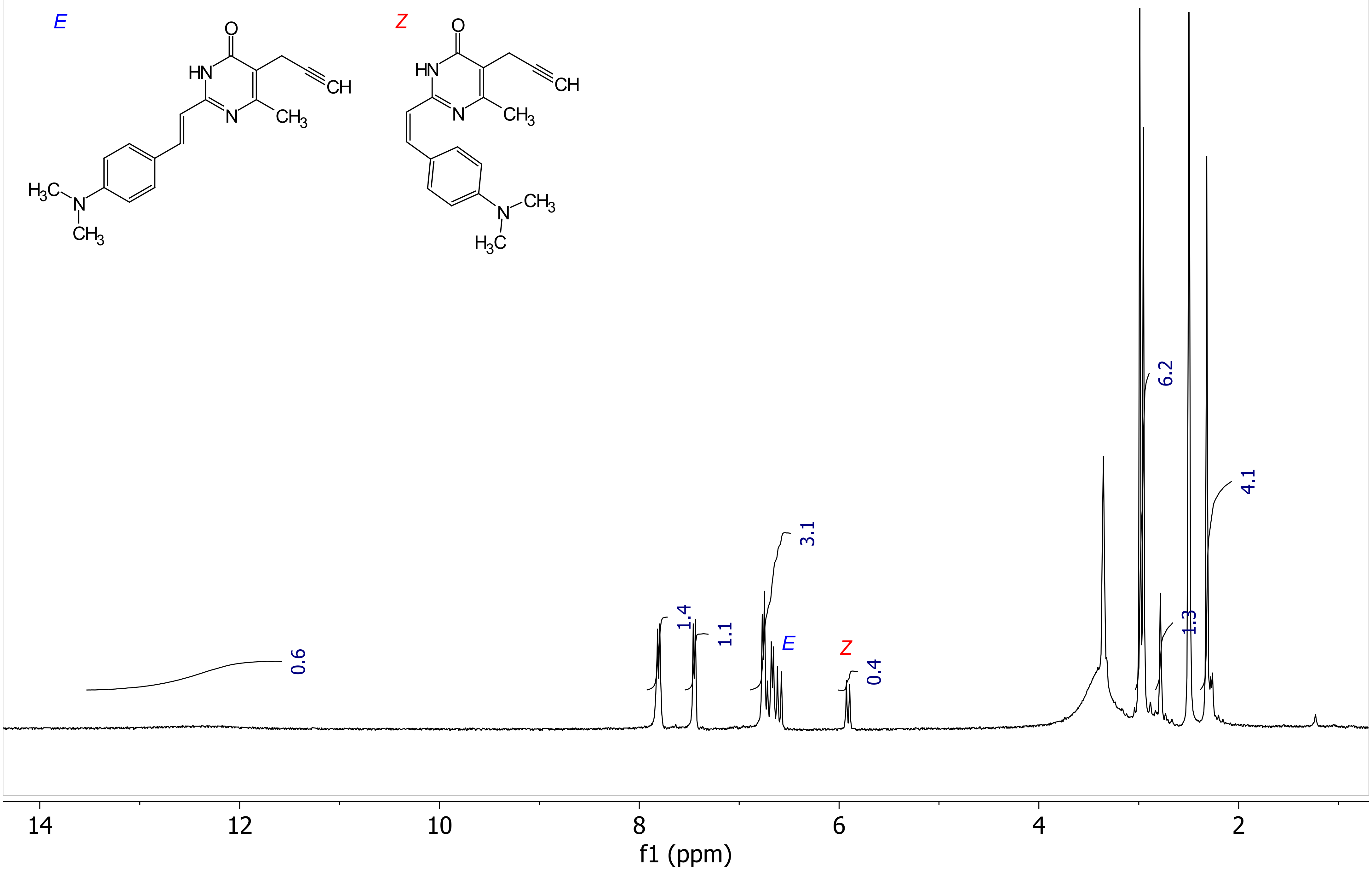

^{1}H NMR spectrum of compound 3a, irr. 3h at 405 nm in DMSO-d_6/CCl_4 1/3, 75% *Z*-isomer

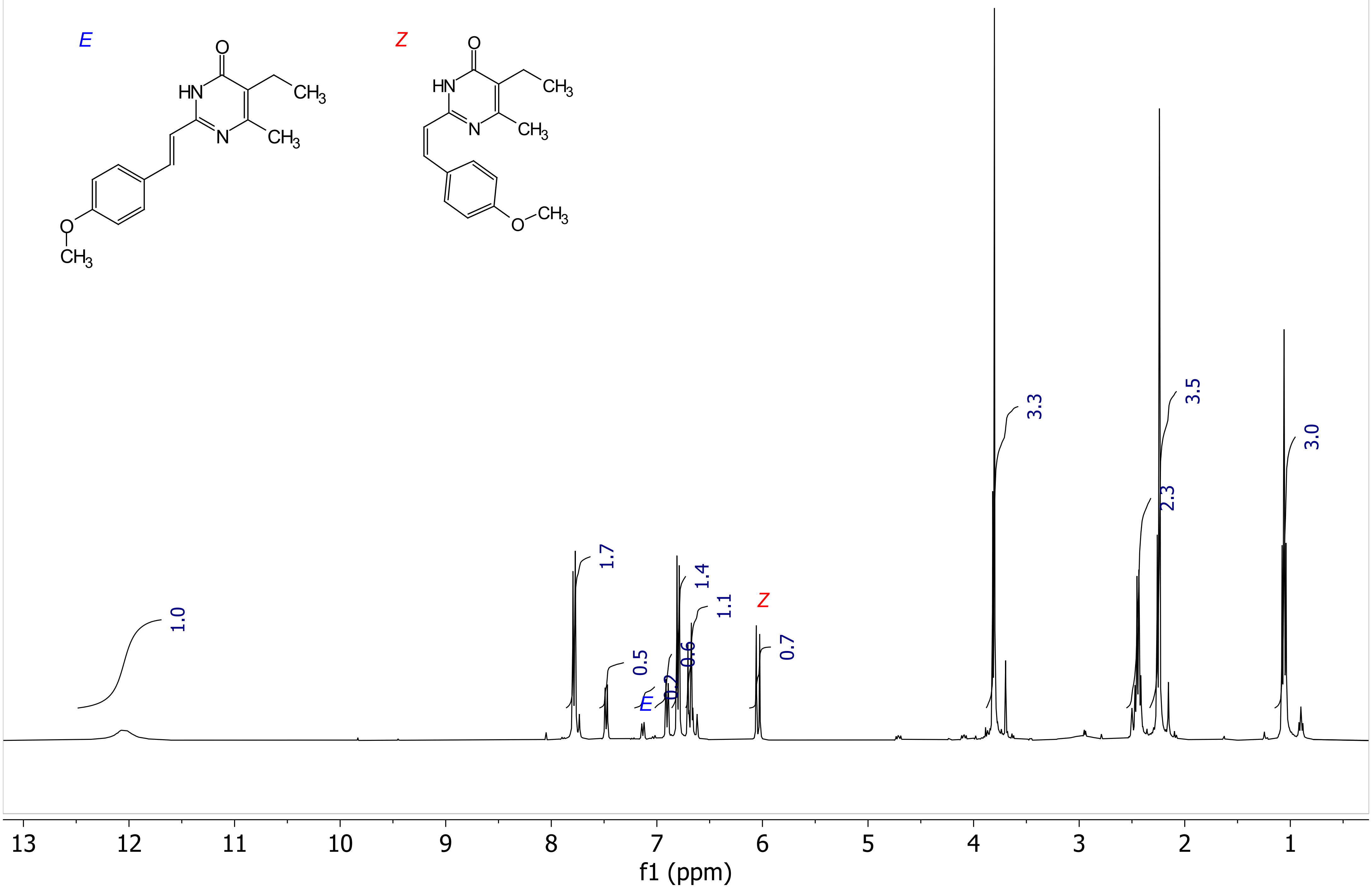

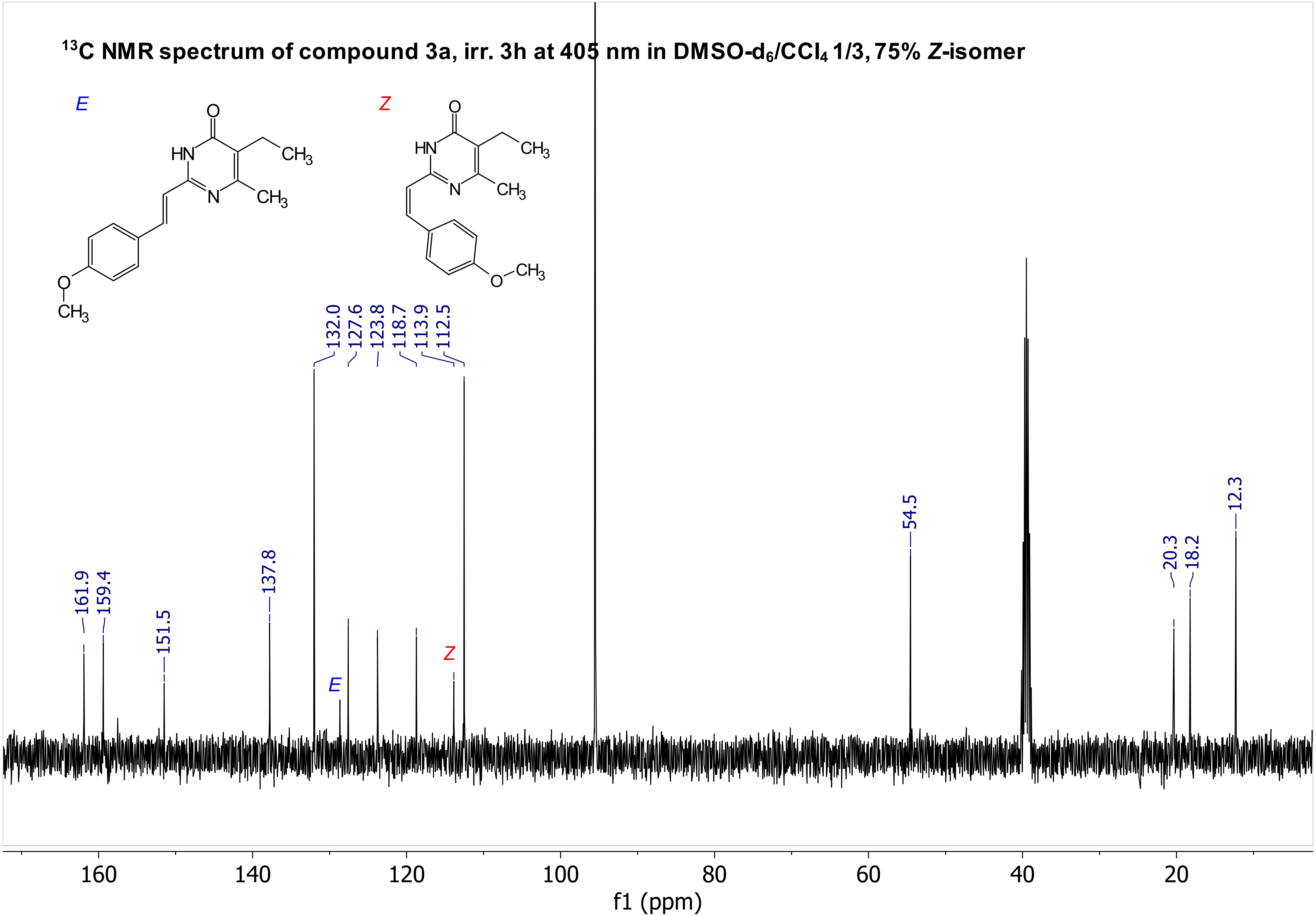
13C NMR spectrum of compound 3a, irr. 3h at 405 nm in DMSO-d6/CCl4 1/3, 75% Z-isomer
E
Z
HN
O
CH3
N
161.9
159.4
151.5
137.8
132.0
127.6
123.8
118.7
113.9
112.5
54.5
20.3
18.2
12.3
160
140
120
100
80
60
40
20
f1 (ppm)

^{1}H-^{13}C HMBC spectrum of compound 3a, irr. 3h at 405 nm in DMSO-d_6/CCl_4 1/3, 75% *Z*-isomer

E

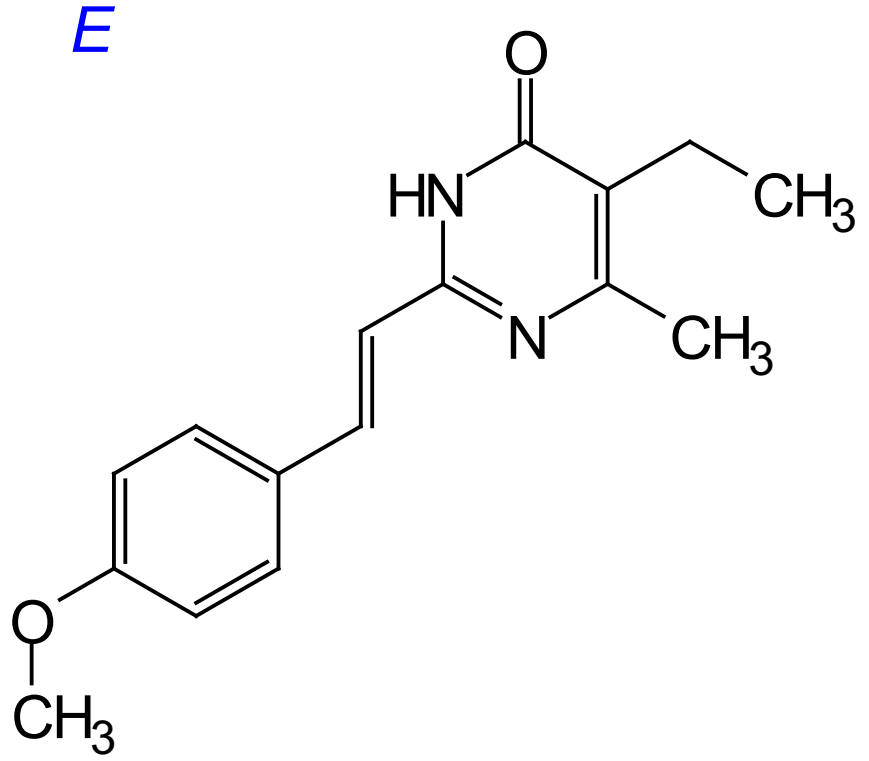

Z

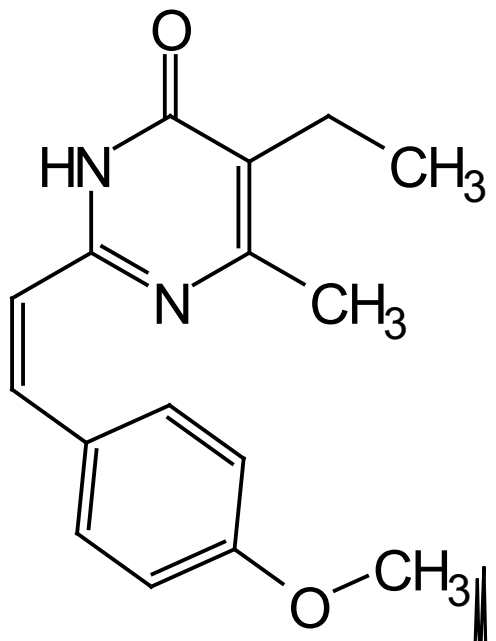

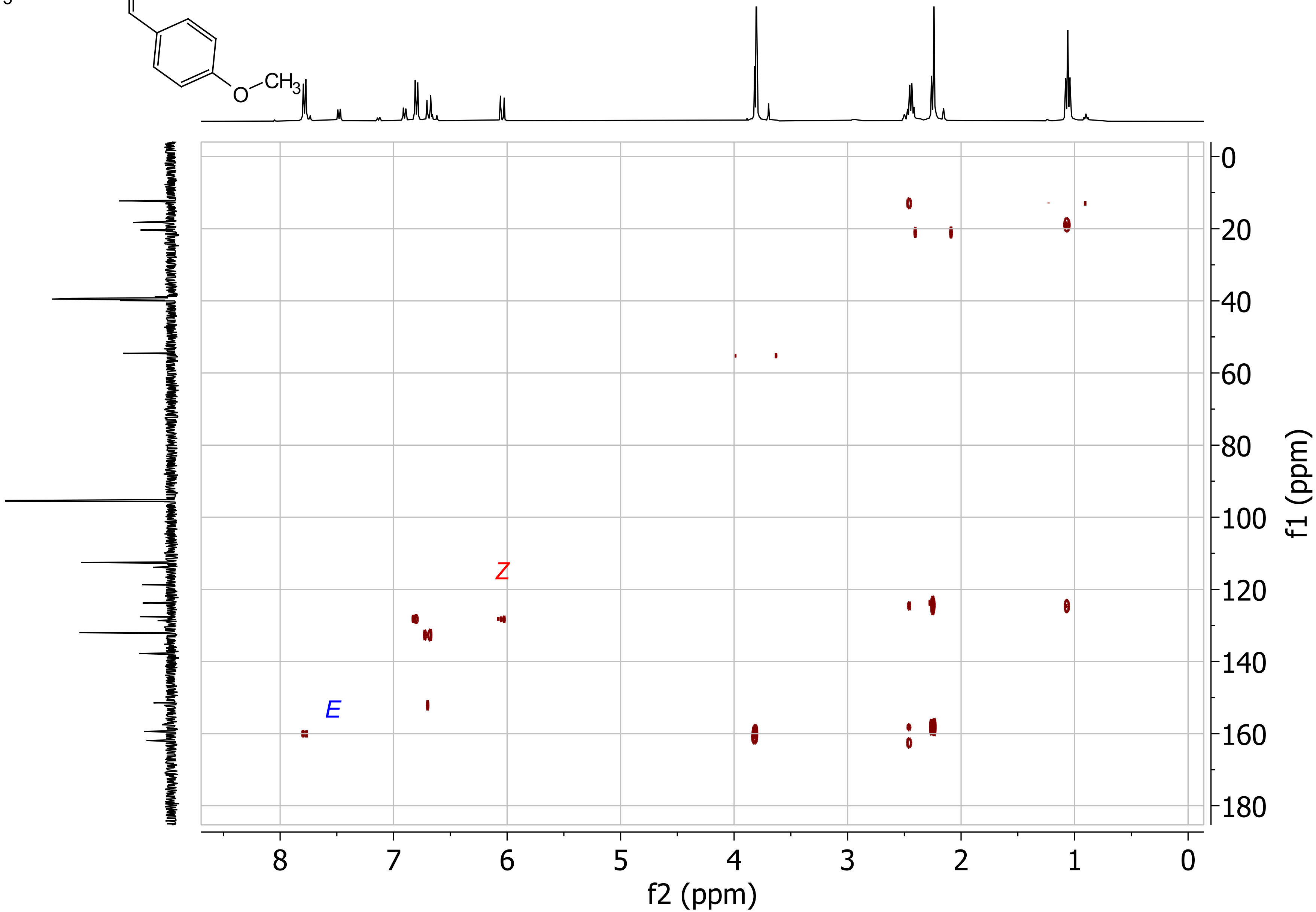

^{1}H-^{13}C HSQC spectrum of compound 3a, irr. 3h at 405 nm in DMSO-d$_6$/CCl$_4$ 1/3, 75% *Z*-isomer

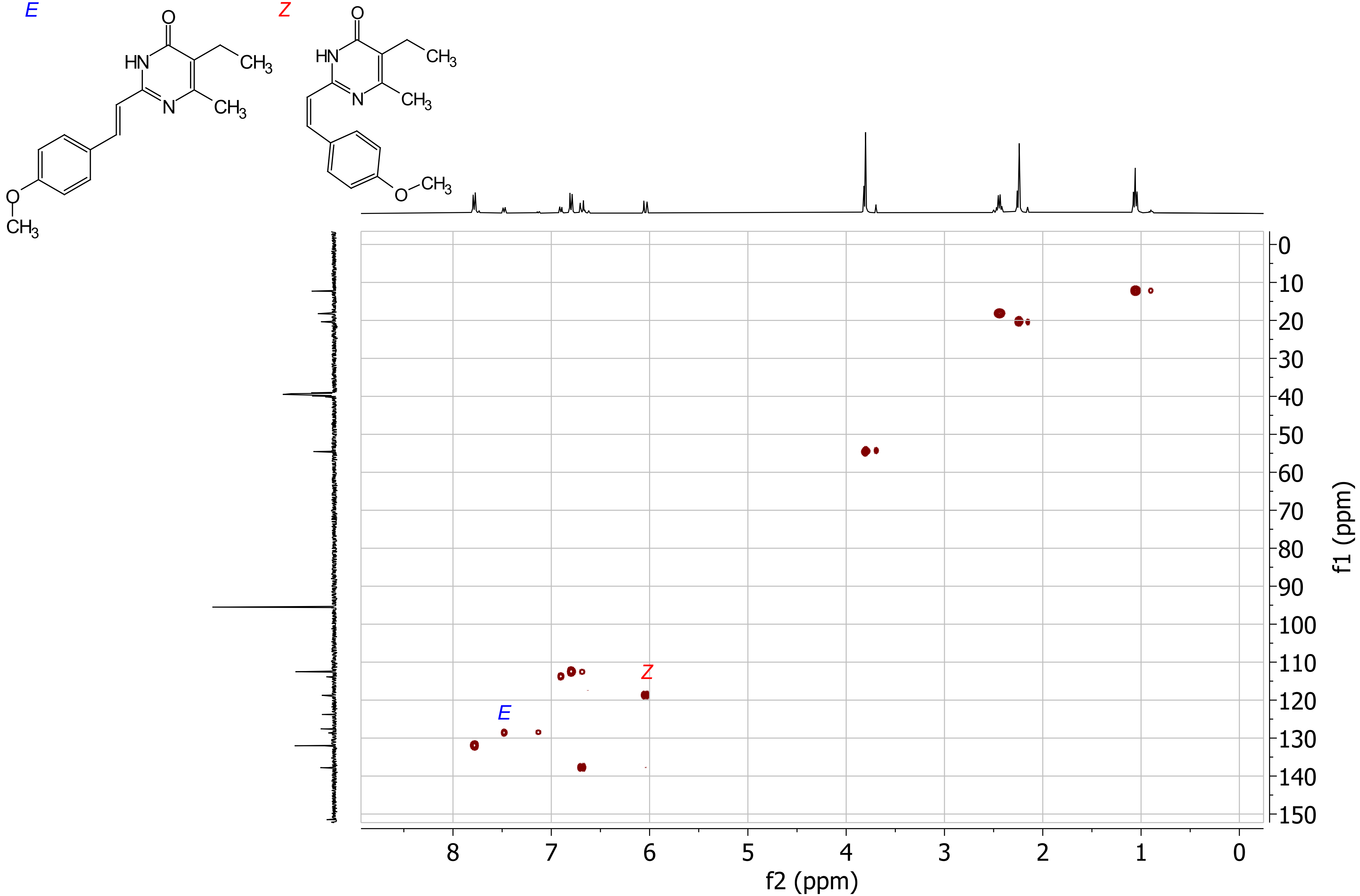

^{1}H NMR spectrum of compound 3b, irr. 1h at 420 nm in DMSO-d_6/CCl_4 1/3, 95% *Z*-isomer

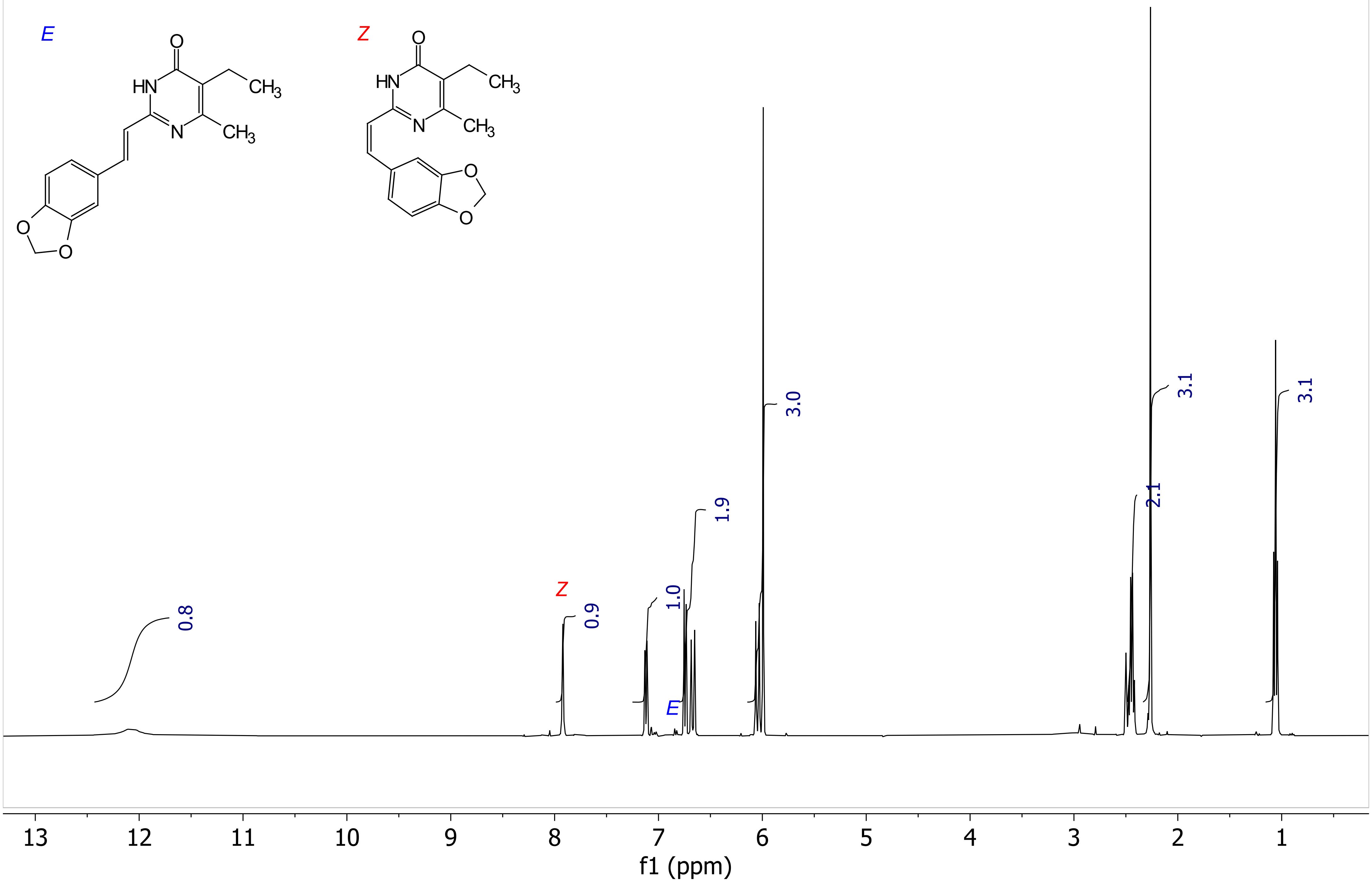

^{13}C NMR spectrum of compound 3b, irr. 1h at 420 nm in DMSO-d_6/CCl_4 1/3, 95% *Z*-isomer

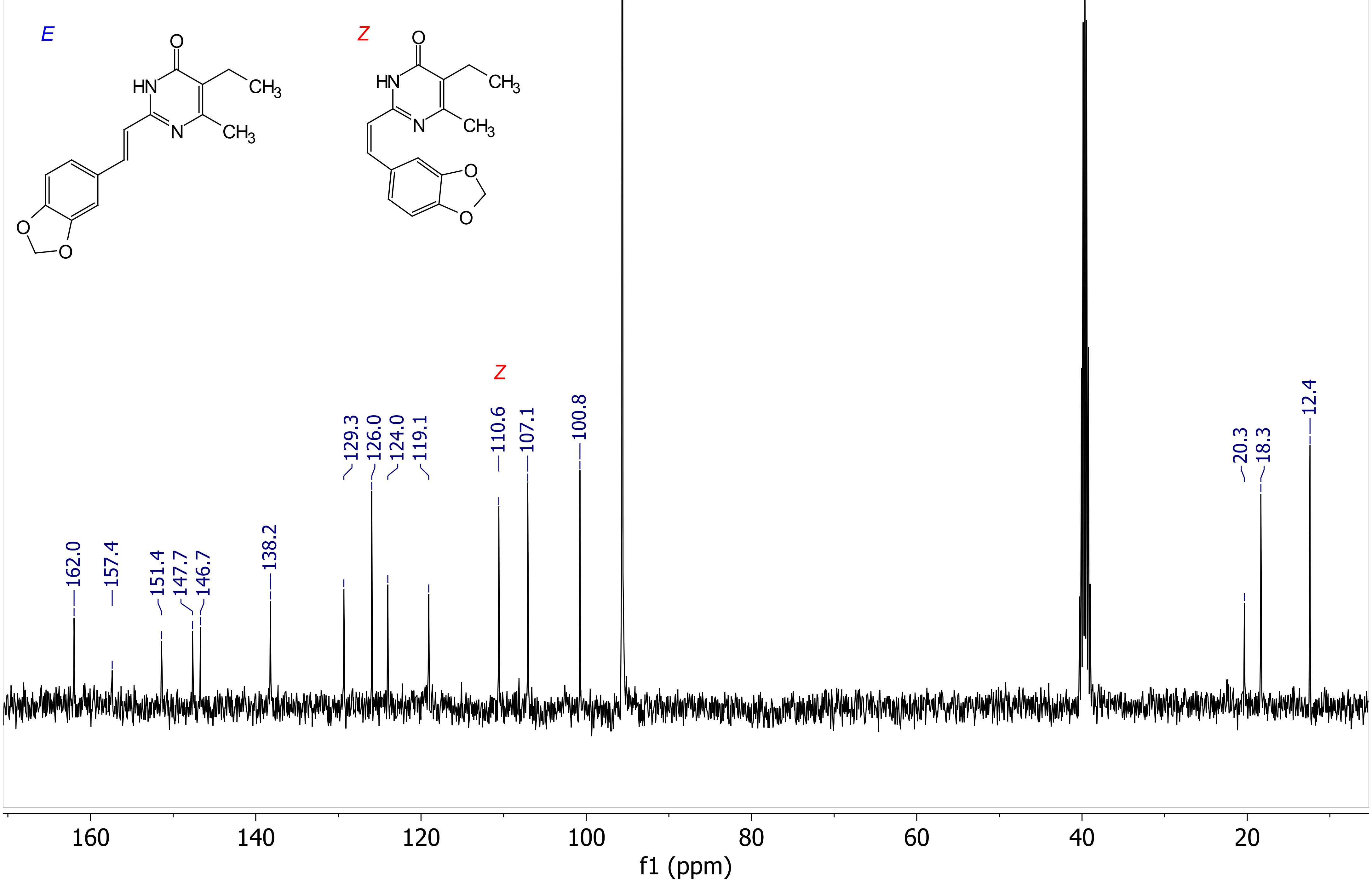

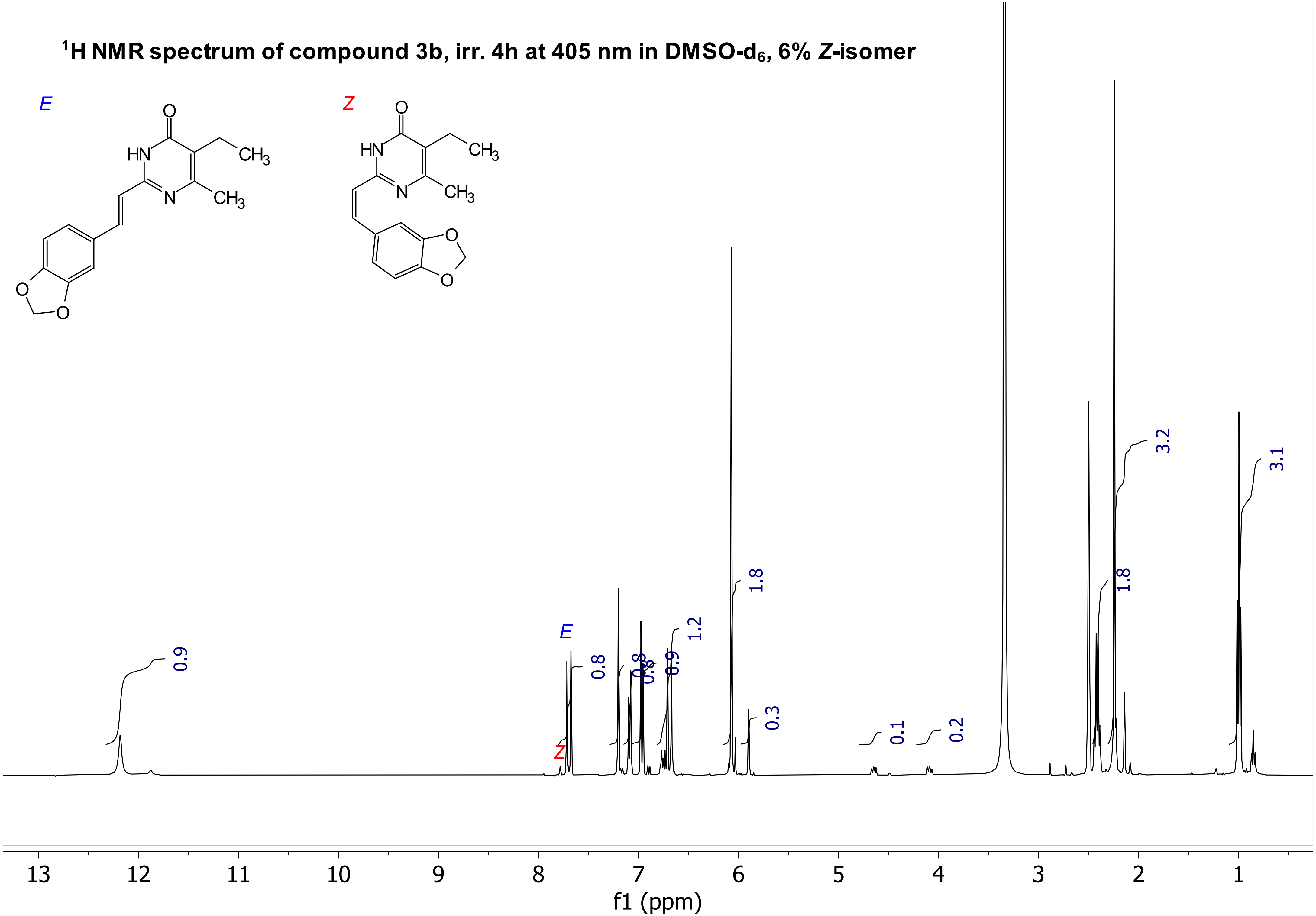

1H NMR spectrum of compound 3b, irr. 4h at 405 nm in DMSO-d6, 6% Z-isomer
E
Z
O
HN
N
CH3
CH3
O
O
0.9
0.8
0.8
0.9
1.2
1.8
0.3
0.1
0.2
1.8
3.2
3.1
E
Z
13
12
11
10
9
8
7
6
5
4
3
2
1
f1 (ppm)

^{1}H NMR spectrum of compound 3b, irr. 3h at 450 nm in $CDCl_3$, 75% *Z*-isomer

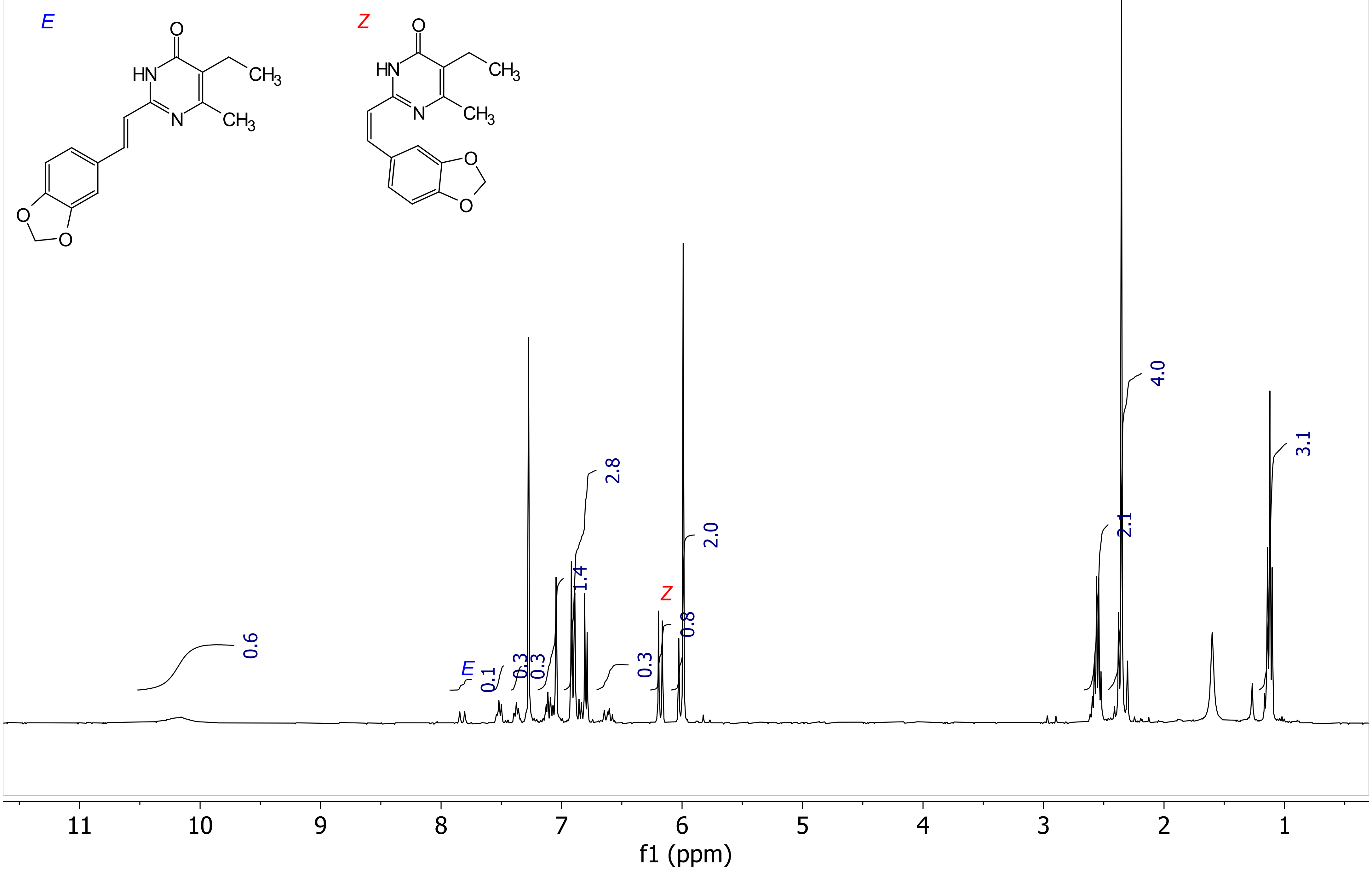

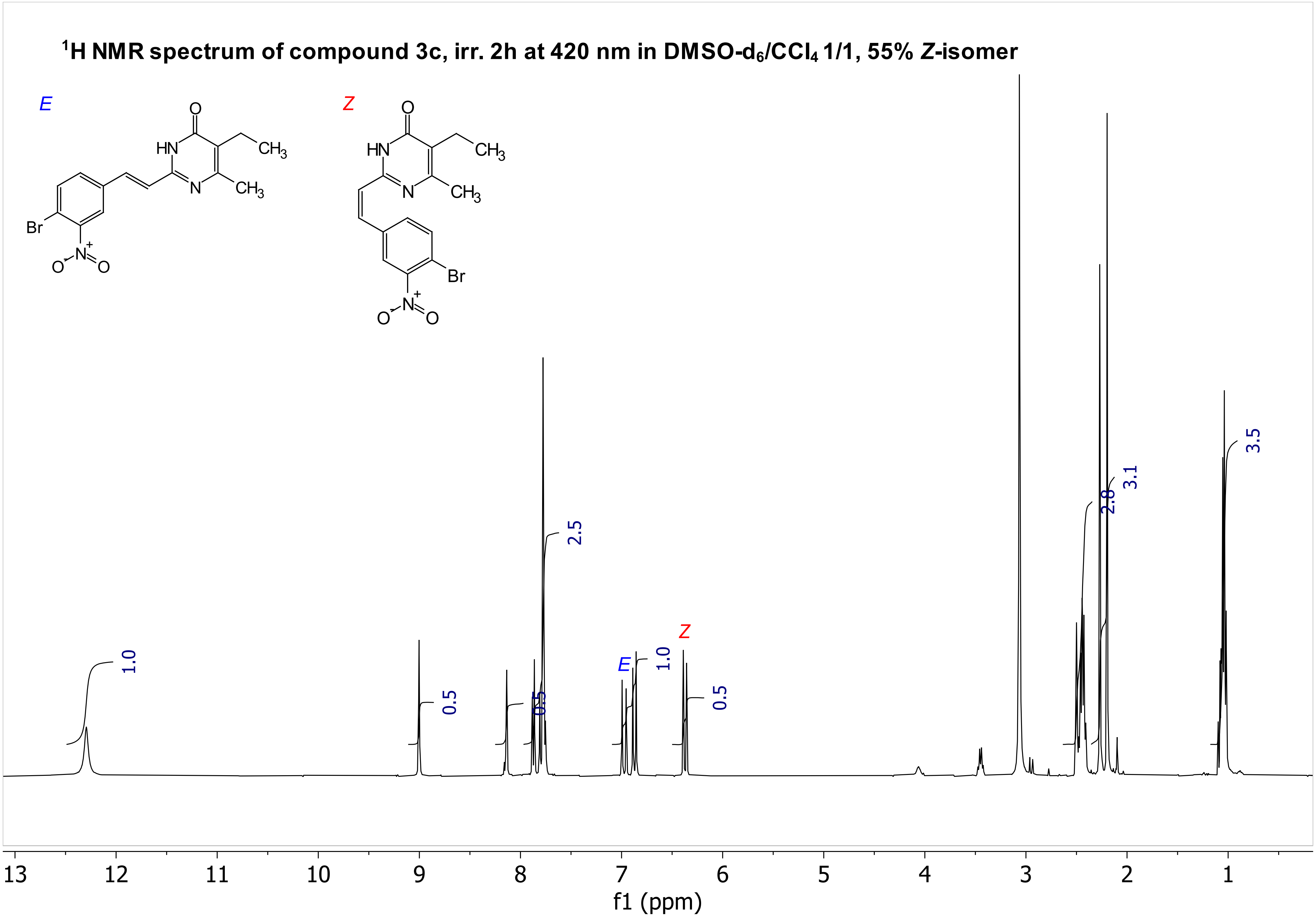

¹H NMR spectrum of compound 3c, irr. 2h at 420 nm in DMSO-d6/CCl4 1/1, 55% Z-isomer
E
Z
1.0
0.5
0.5
2.5
E
1.0
Z
0.5
2.8
3.1
3.5
f1 (ppm)

^{1}H NMR spectrum of compound 3d, irr. 2h at 420 nm in DMSO-d_6/CCl_4 1/1, 45% *Z*-isomer

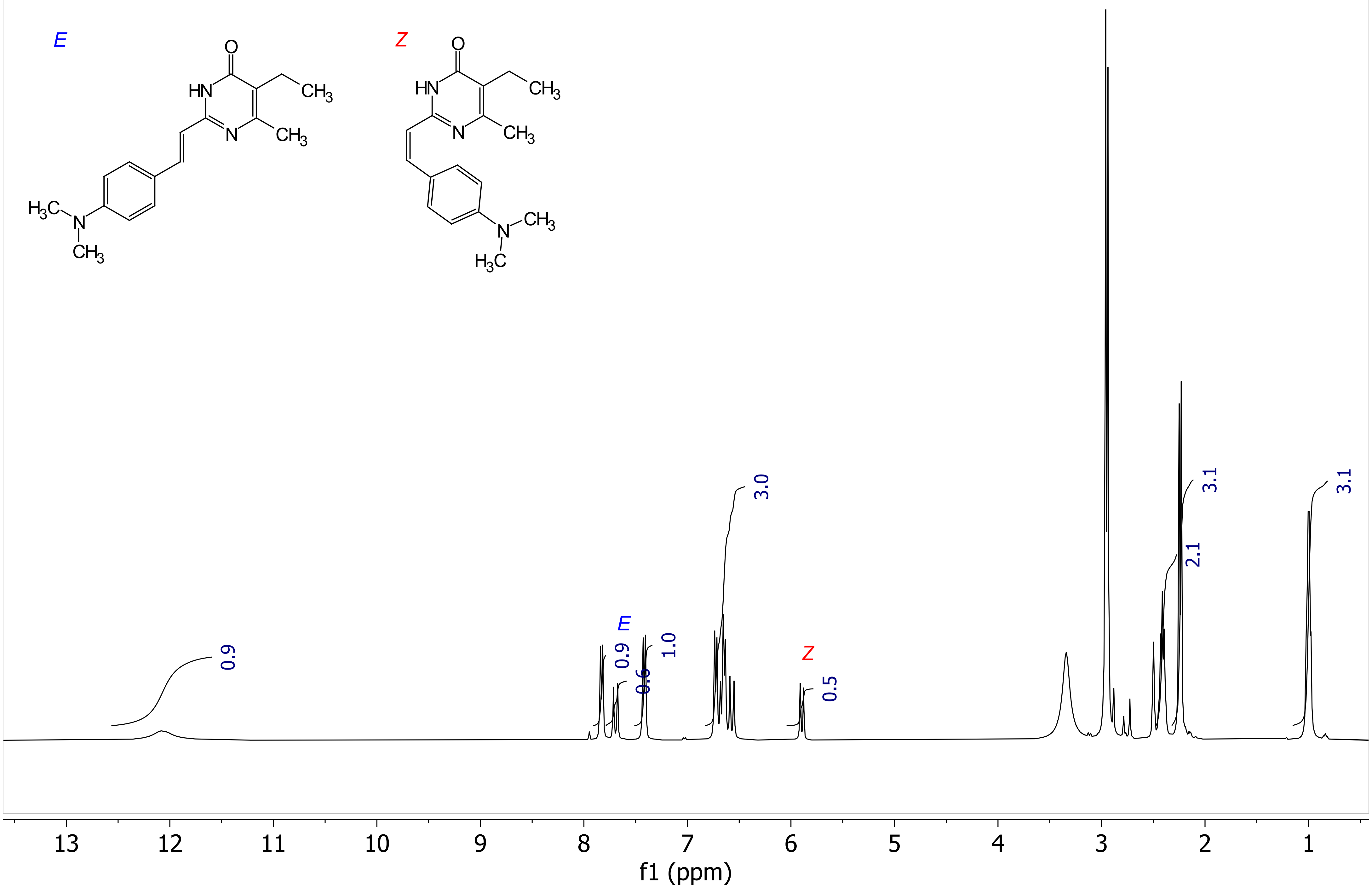

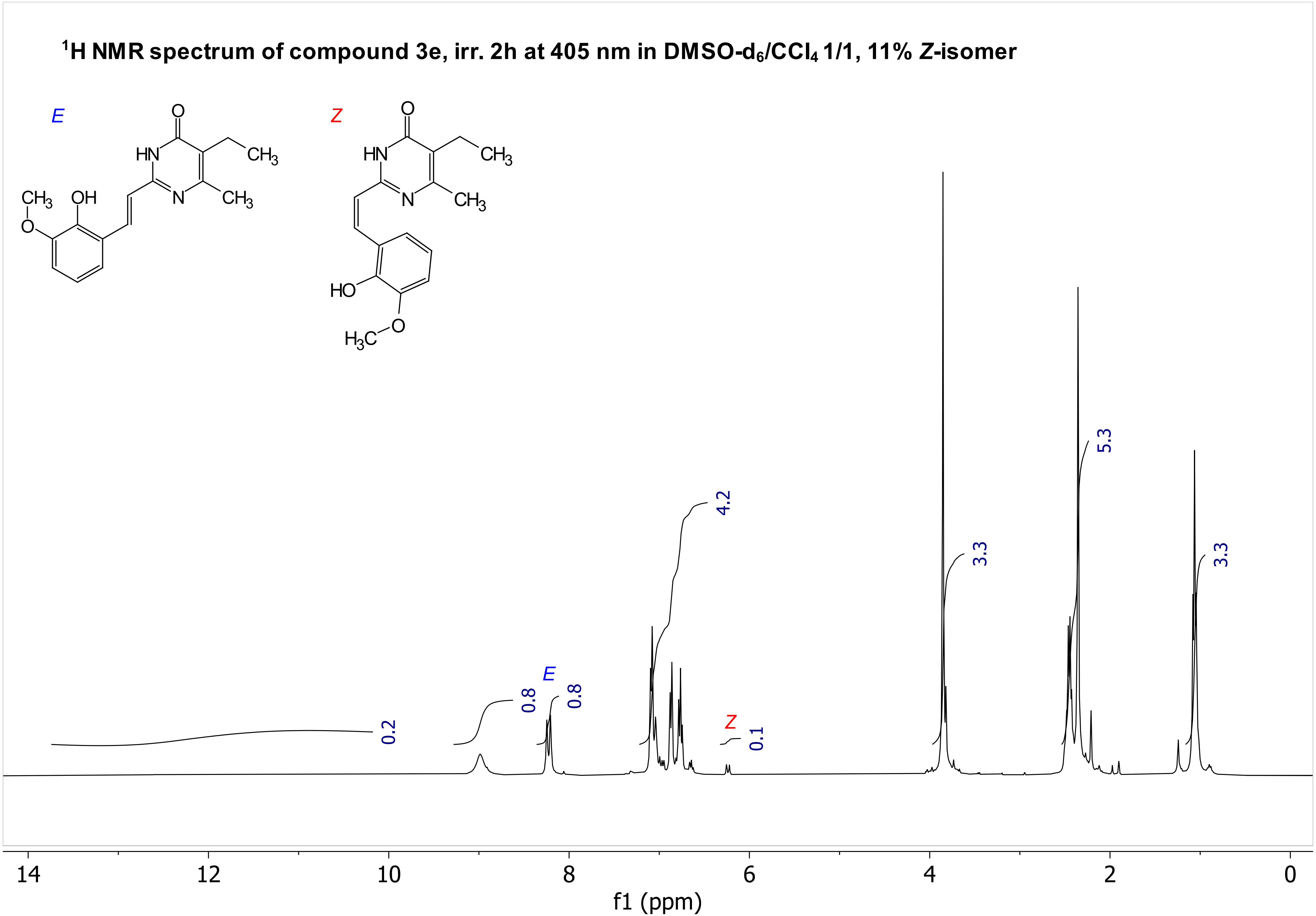

^{1}H NMR spectrum of compound 3e, irr. 2h at 405 nm in DMSO-d_6/CCl_4 1/1, 11% *Z*-isomer
E
Z
HN
O
CH3
CH3
OH
N
O
HO
H3C
0.2
0.8
0.8
4.2
0.1
3.3
5.3
3.3
14
12
10
8
6
4
2
0
f1 (ppm)

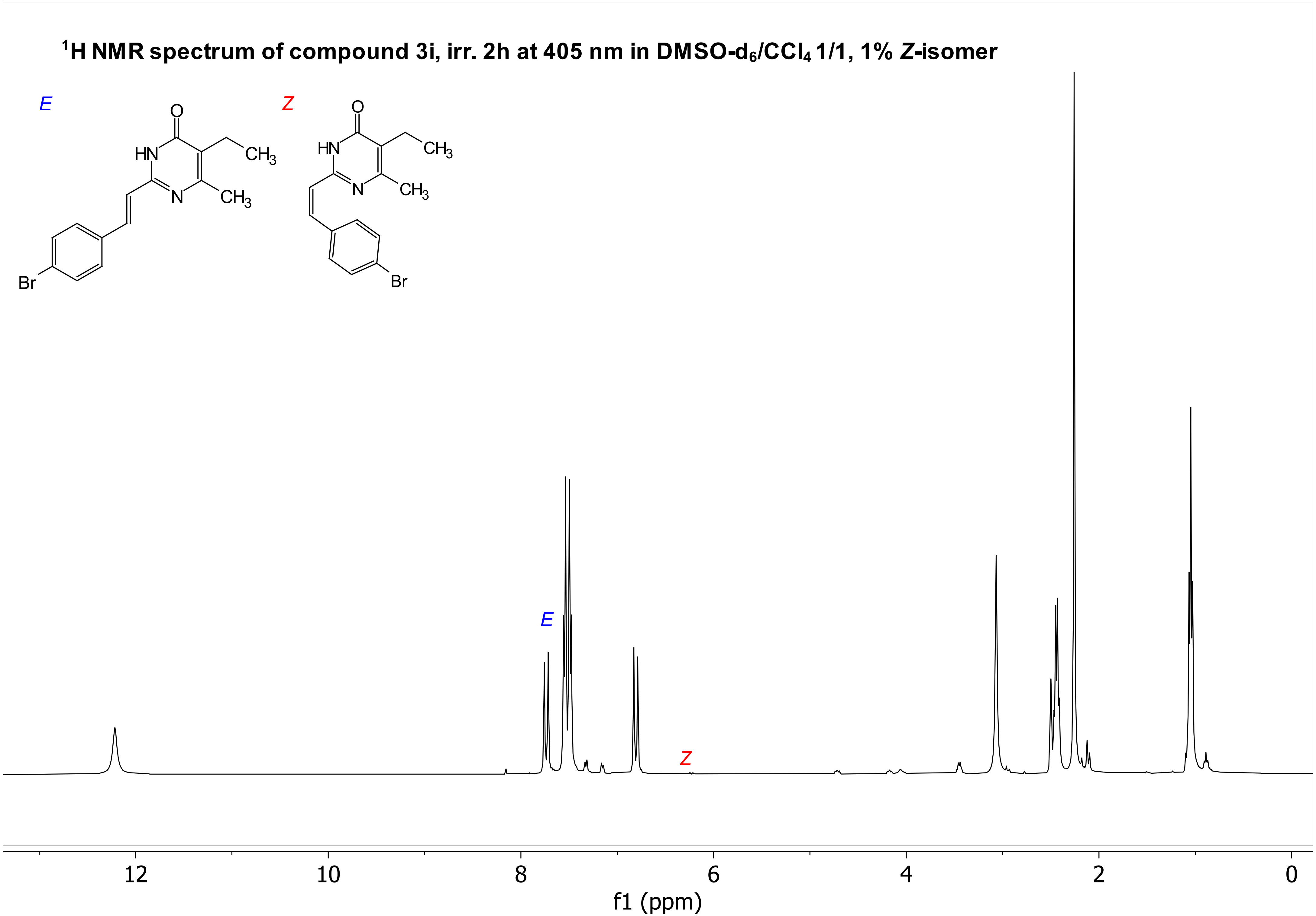

^{1}H NMR spectrum of compound 3i, irr. 2h at 405 nm in DMSO-d$_6$/CCl$_4$ 1/1, 1% *Z*-isomer
E
Z
O
HN
N
CH3
CH3
Br
E
Z
12
10
8
6
4
2
0
f1 (ppm)

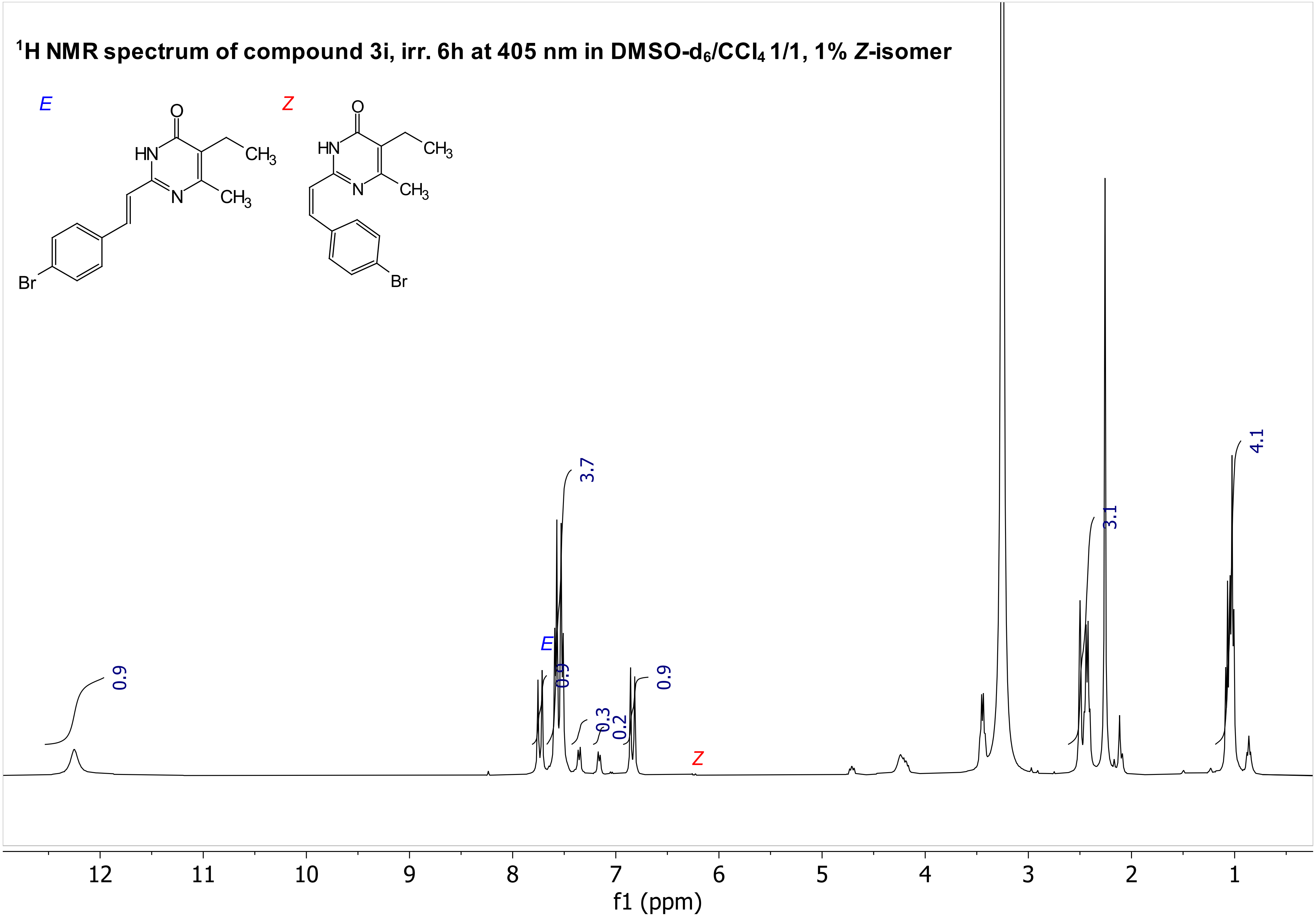

1H NMR spectrum of compound 3i, irr. 6h at 405 nm in DMSO-d6/CCl4 1/1, 1% Z-isomer
E
Z
O
HN
N
CH3
CH3
Br
0.9
3.7
0.9
0.3
0.2
0.9
3.1
4.1
12
11
10
9
8
7
6
5
4
3
2
1
f1 (ppm)

^{1}H NMR spectrum of compound 4a, irr. 10h at 420 nm in DMSO-d_6/CCl_4 1/1, 76% *Z*-isomer

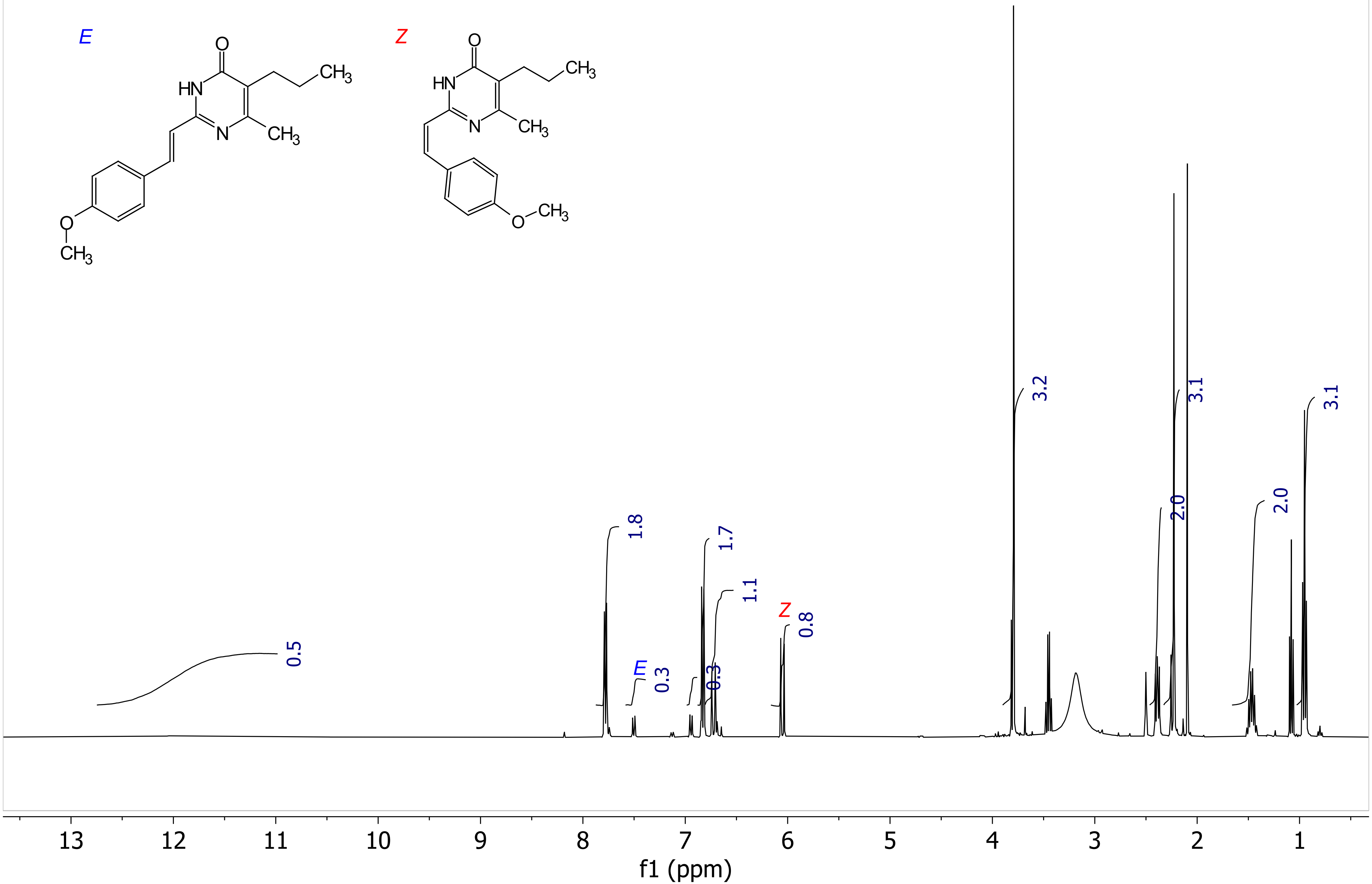

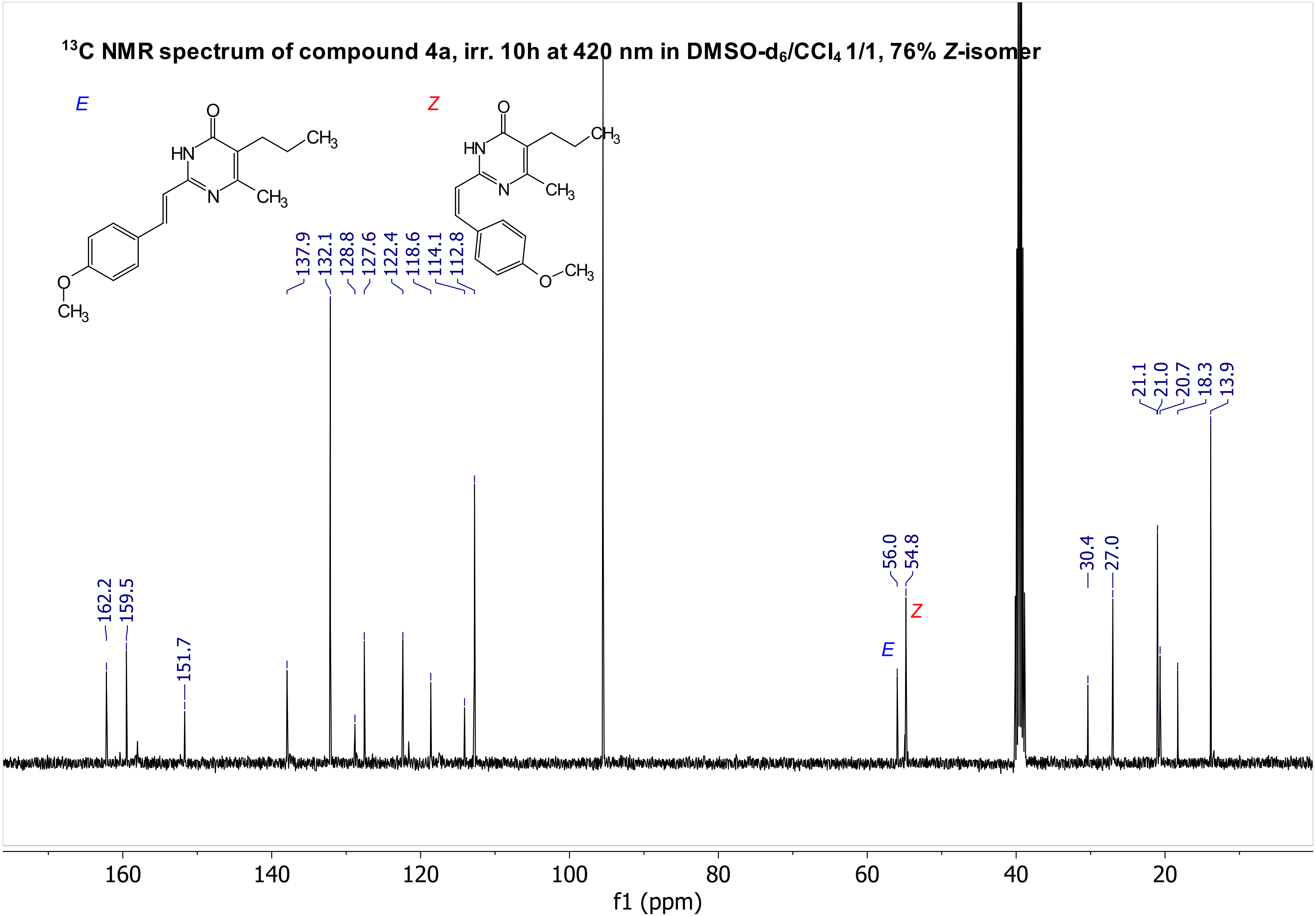

^{13}C NMR spectrum of compound 4a, irr. 10h at 420 nm in DMSO-d_6/CCl_4 1/1, 76% *Z*-isomer

NOESY spectrum of compound 4a, irr. 10h at 420 nm in DMSO-d_6/CCl_4 1/1, 76% *Z*-isomer

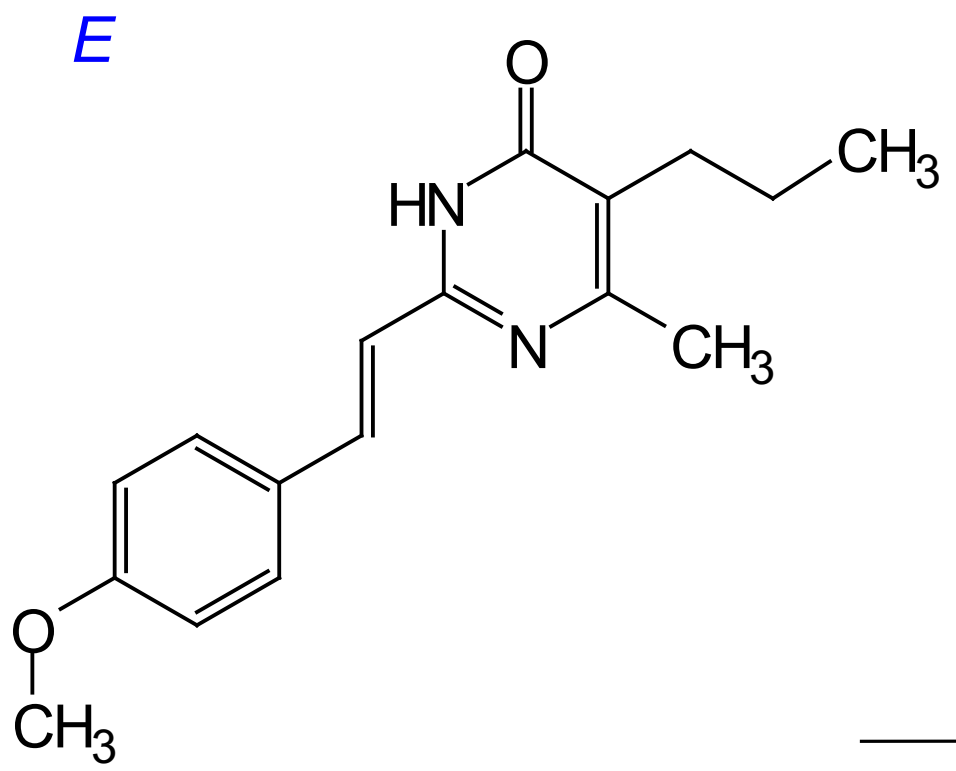

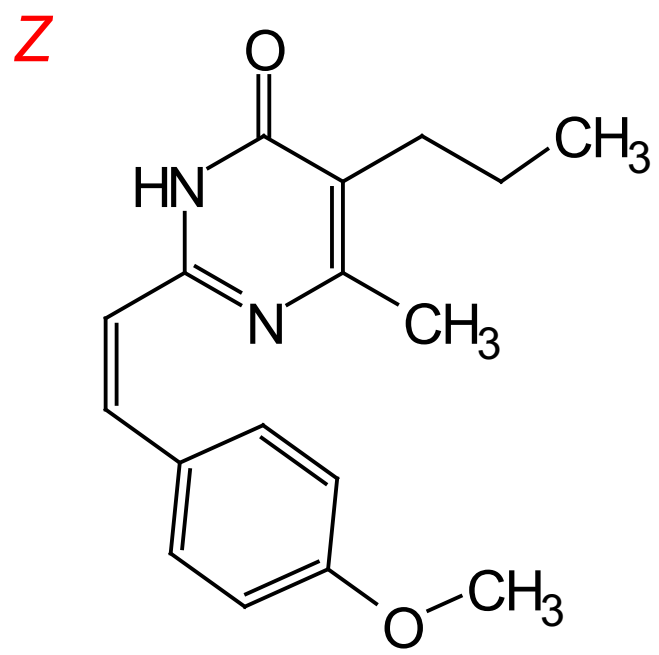

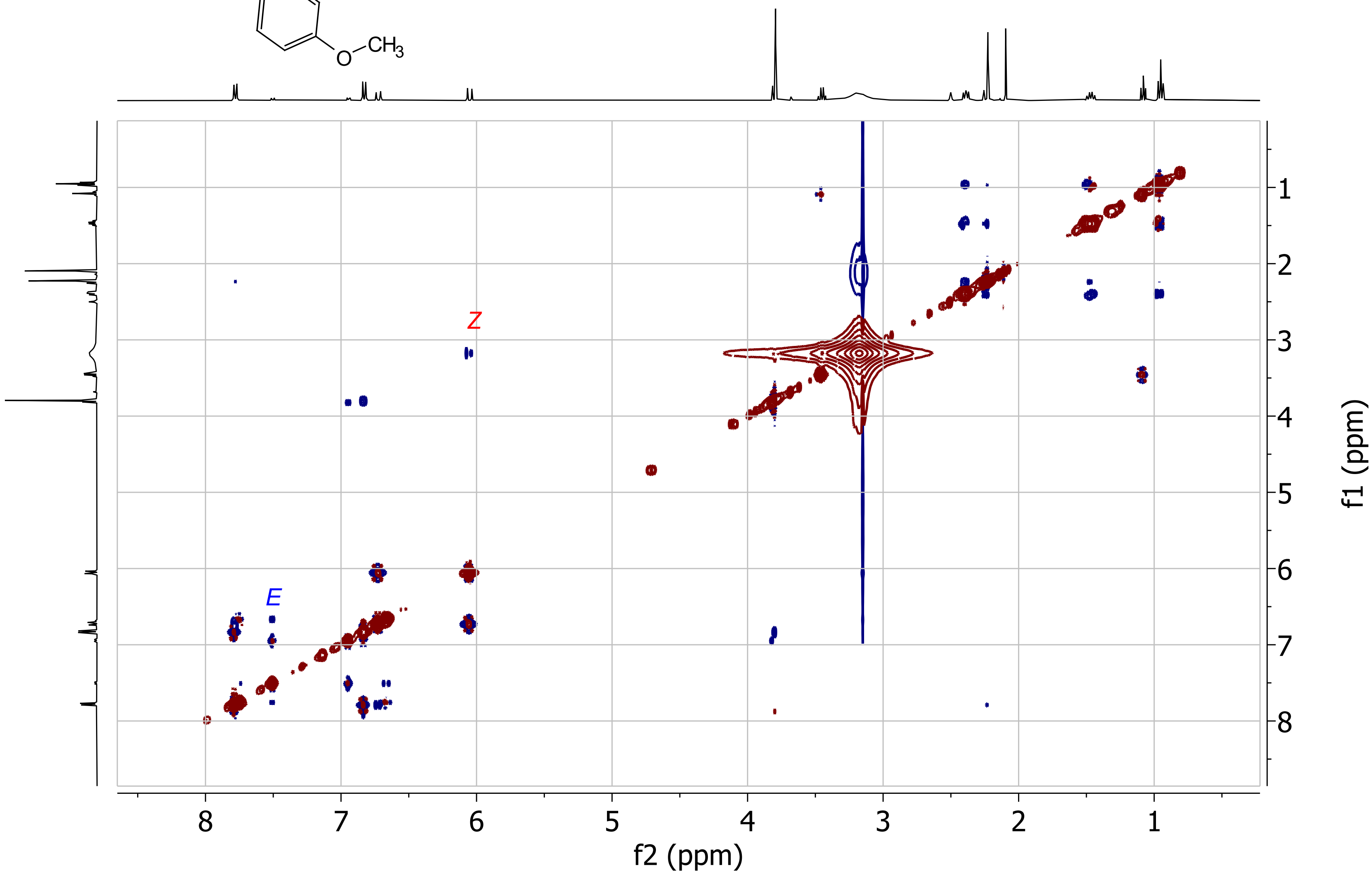

^{1}H NMR spectrum of compound 4b, irr. 7h at 420 nm in DMSO-d_6, 72% D monomer-equivalent cyclodimerized material

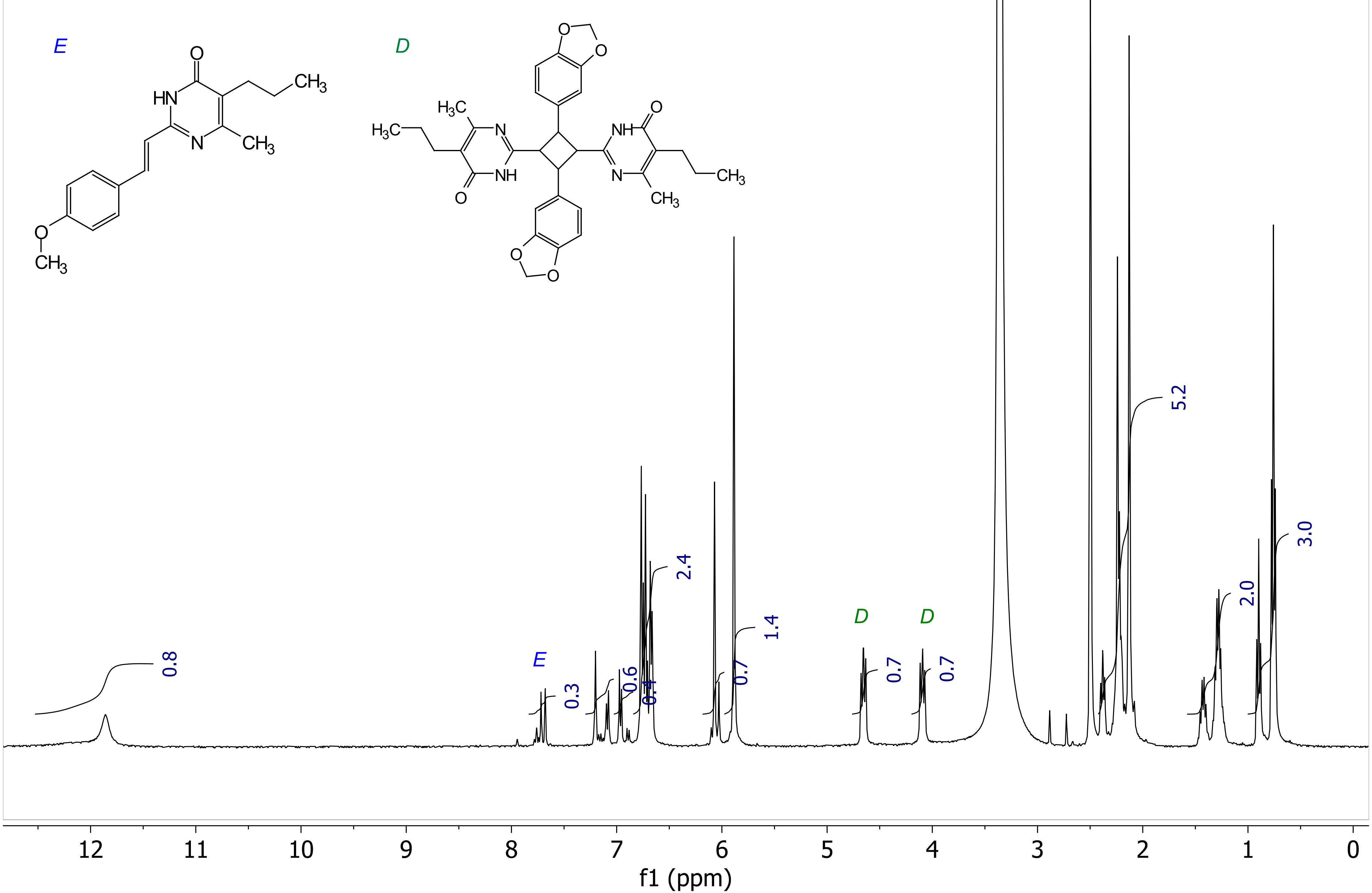

NOESY spectrum of compound 4b, irr. 7h at 420 nm in DMSO-d_6, 72% D monomer-equivalent cyclodimerized material

D

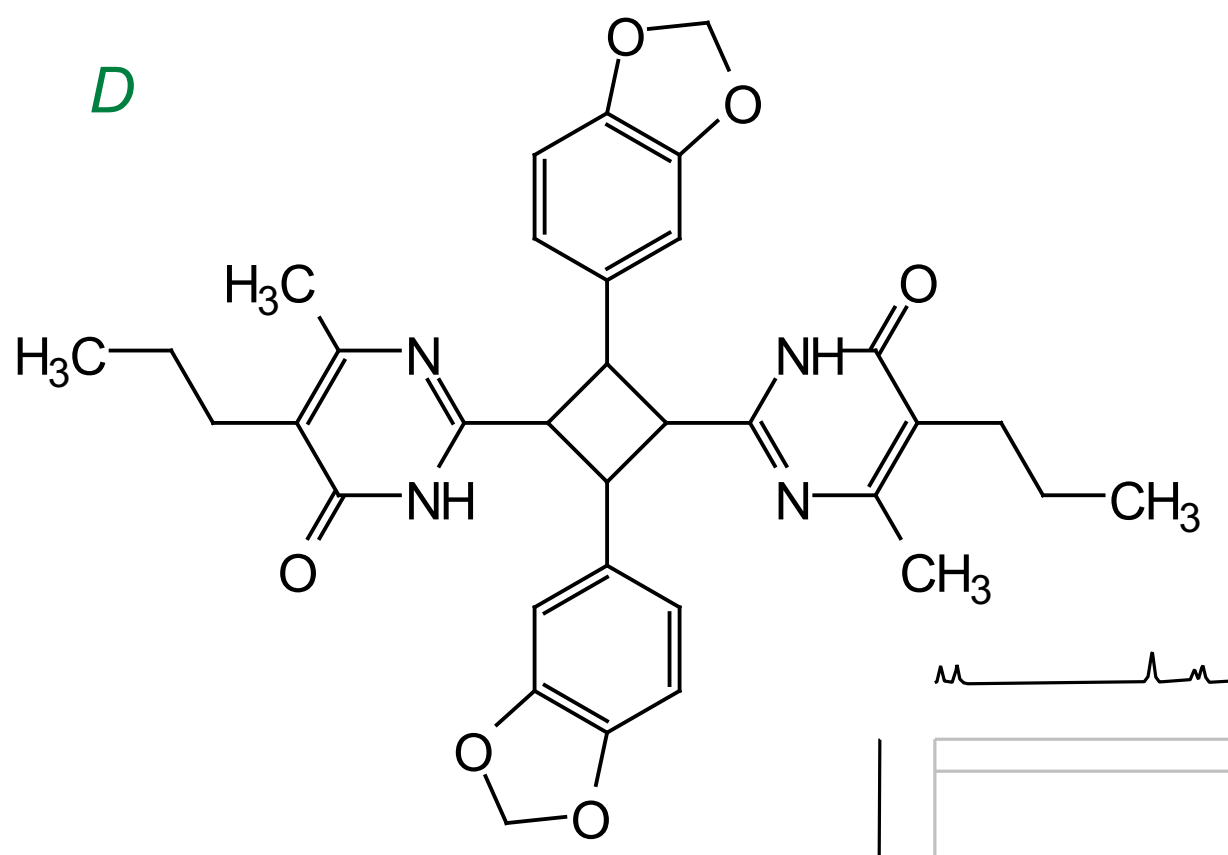

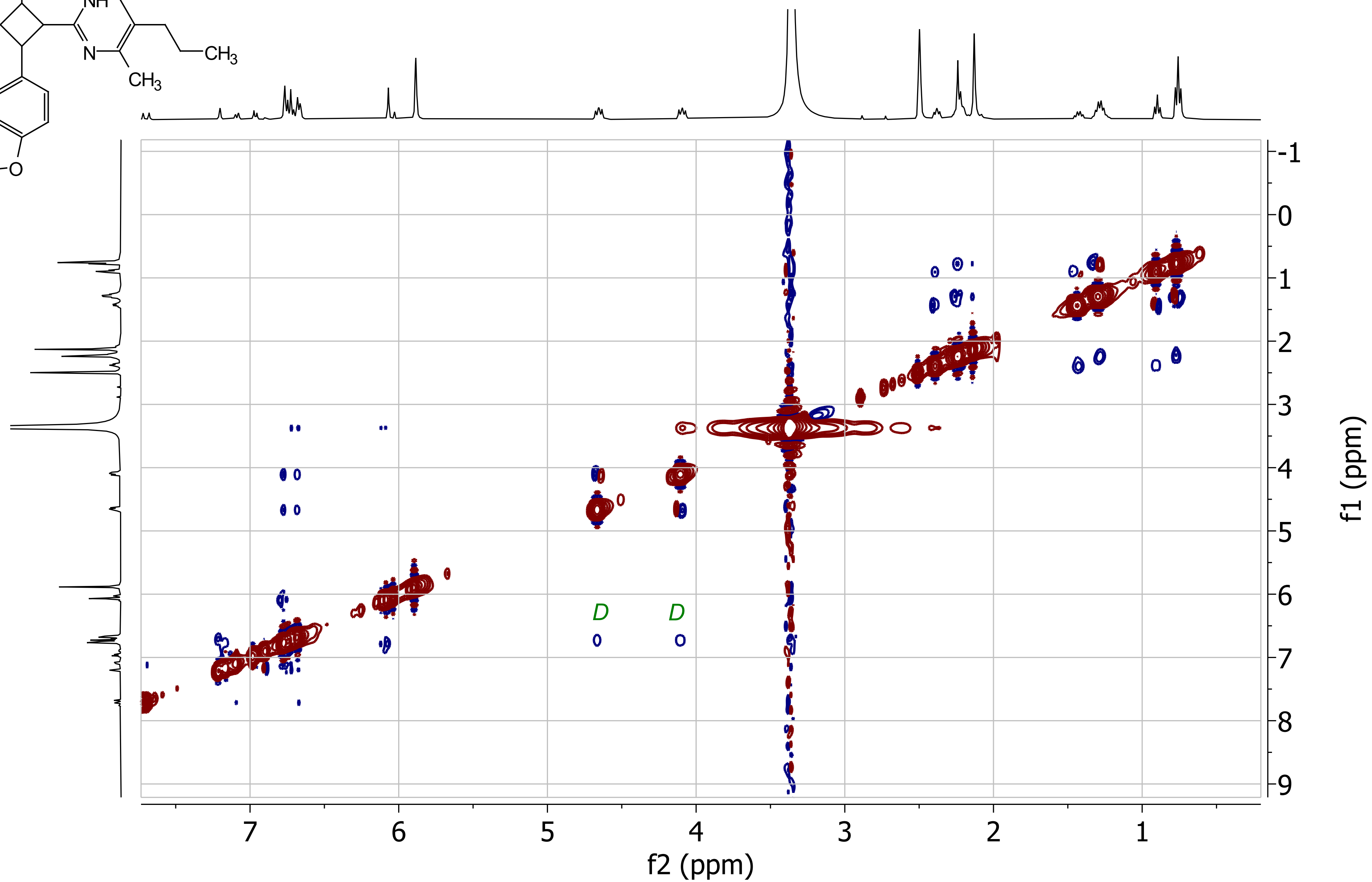

^{1}H NMR spectrum of compound 4b, irr. 3h at 420 nm in $CDCl_3$, 58% *Z*-isomer

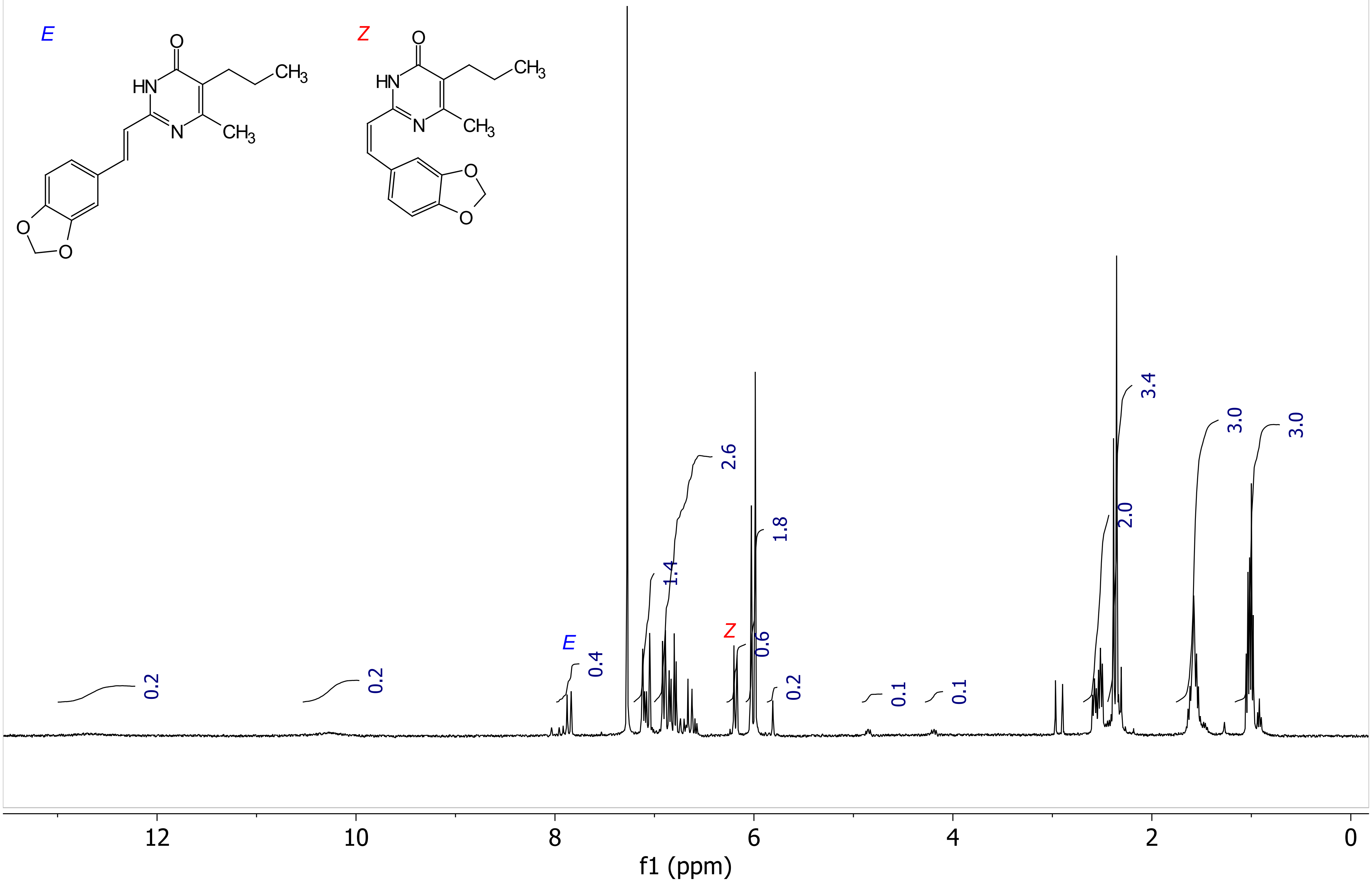

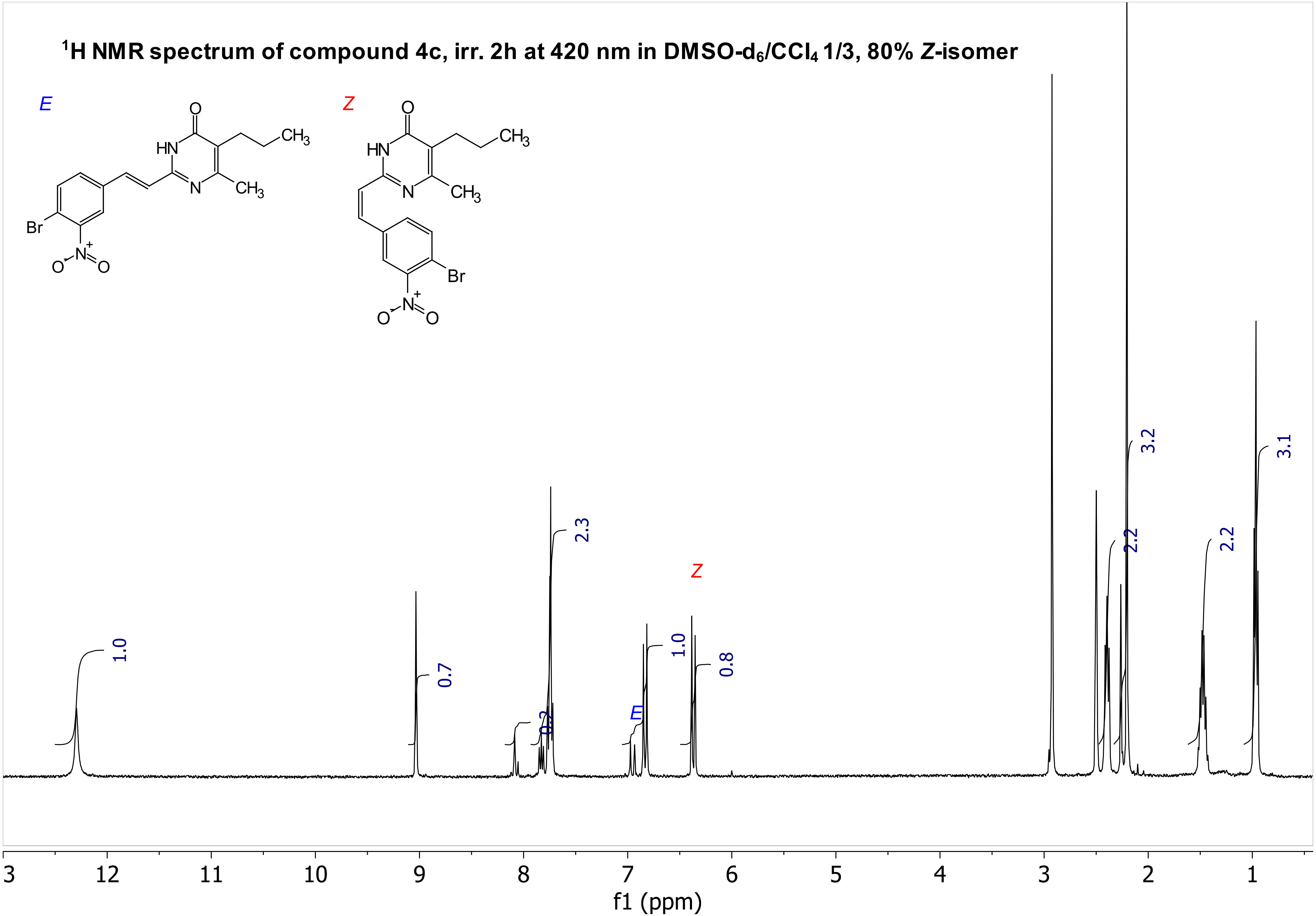

1H NMR spectrum of compound 4c, irr. 2h at 420 nm in DMSO-d6/CCl4 1/3, 80% Z-isomer
E
Z
O
HN
N
CH3
CH3
Br
N+
O-
O
1.0
0.7
0.2
2.3
E
1.0
Z
0.8
2.2
3.2
2.2
3.1
3
12
11
10
9
8
7
6
5
4
3
2
1
f1 (ppm)

^{1}H NMR spectrum of compound 4c, irr. 2h at 405 nm in DMSO-d_6, 56% *Z*-isomer

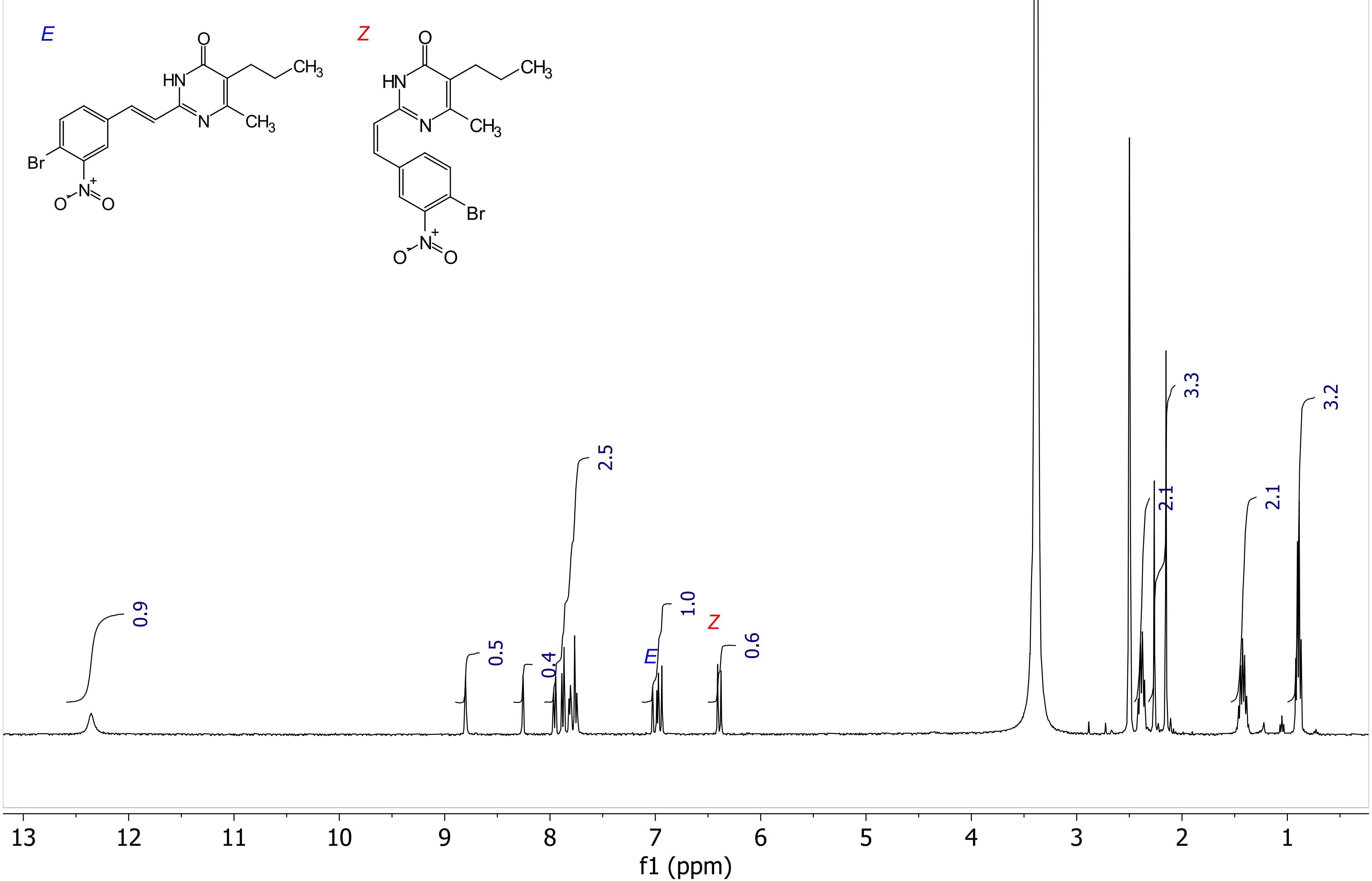

^{1}H-^{13}C HSQC spectrum of compound 4c, irr. 2h at 420 nm in DMSO-d_6/CCl_4 1/3, 80% *Z*-isomer

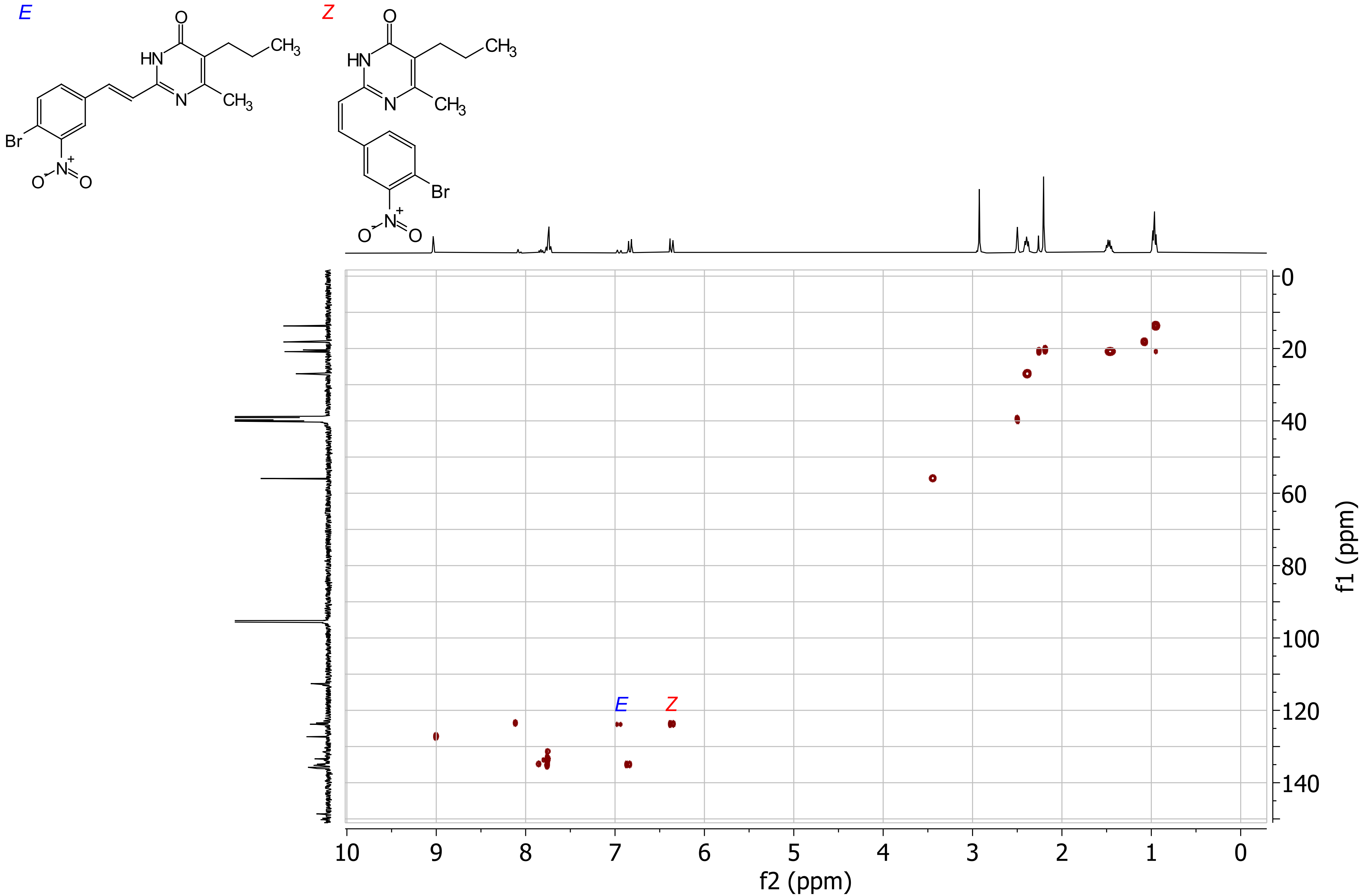

^{1}H NMR spectrum of compound 5a, irr. 10h at 420 nm in DMSO-d_6/CCl_4 1/1, 79% *Z*-isomer

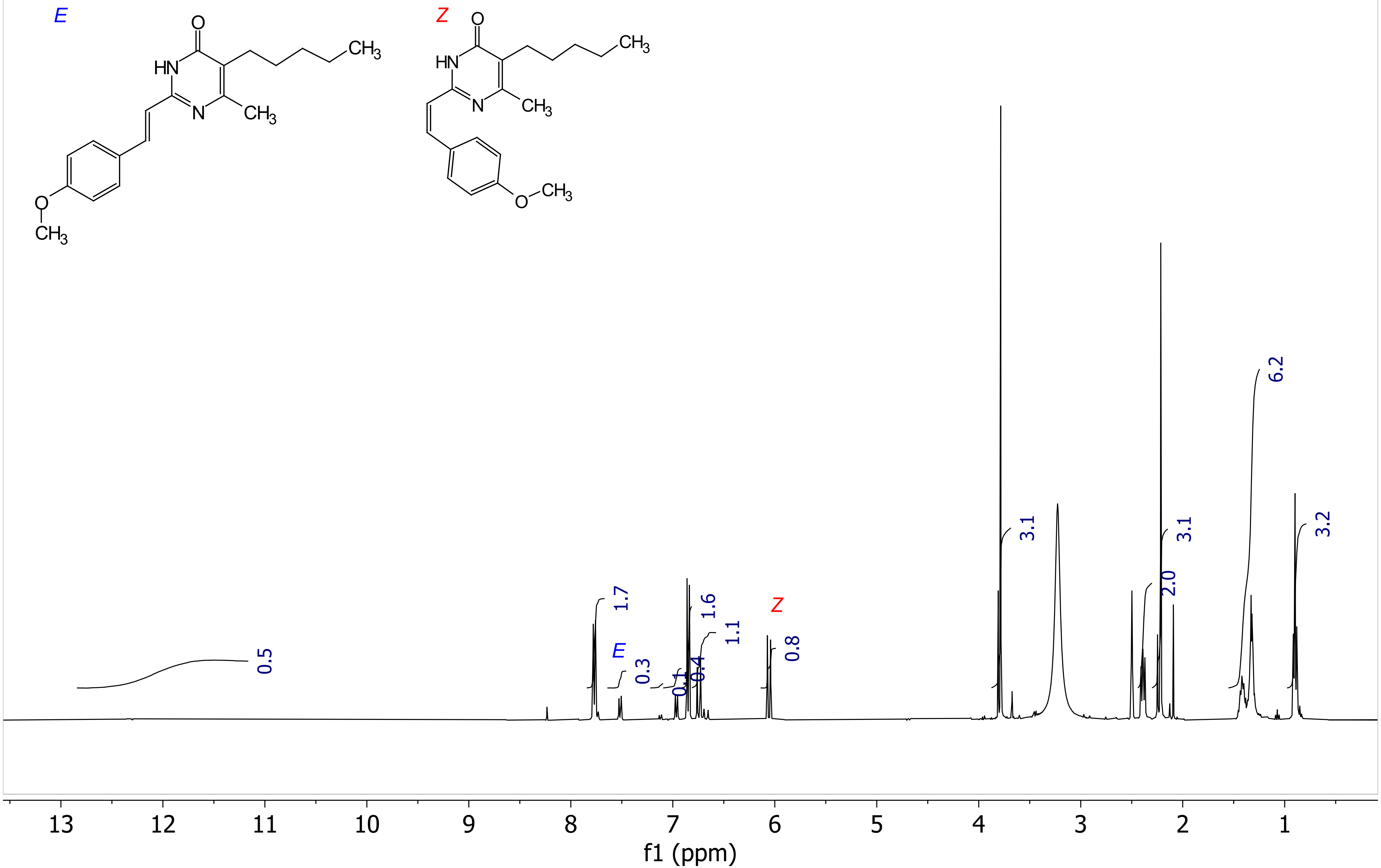

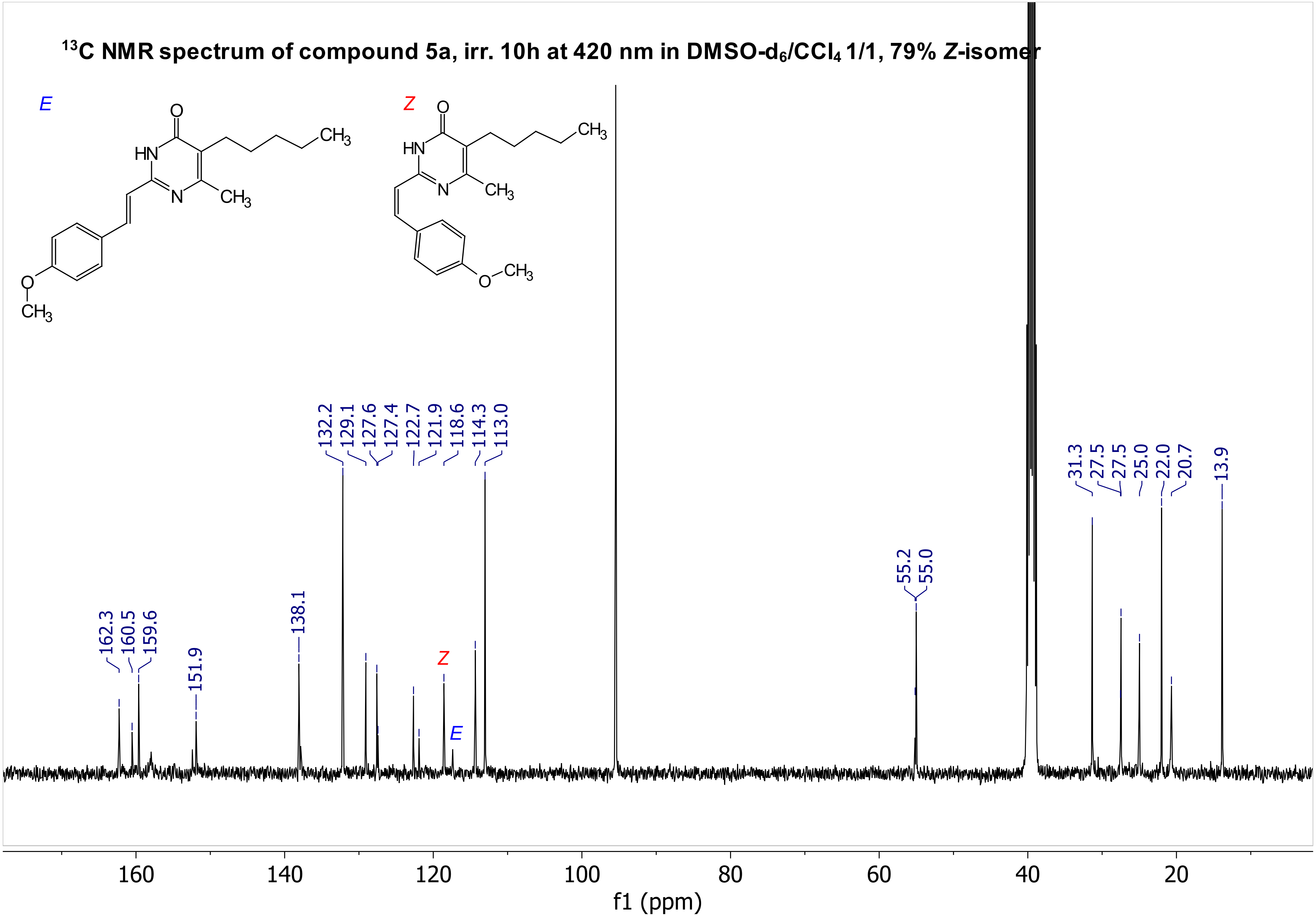

^{13}C NMR spectrum of compound 5a, irr. 10h at 420 nm in DMSO-d$_6$/CCl$_4$ 1/1, 79% Z-isomer
E
Z
162.3
160.5
159.6
151.9
138.1
132.2
129.1
127.6
127.4
122.7
121.9
118.6
114.3
113.0
55.2
55.0
31.3
27.5
27.5
25.0
22.0
20.7
13.9
f1 (ppm)

^{1}H-^{13}C HSQC spectrum of compound 5a, irr. 10h at 420 nm in DMSO-d_6/CCl_4 1/1, 79% *Z*-isomer

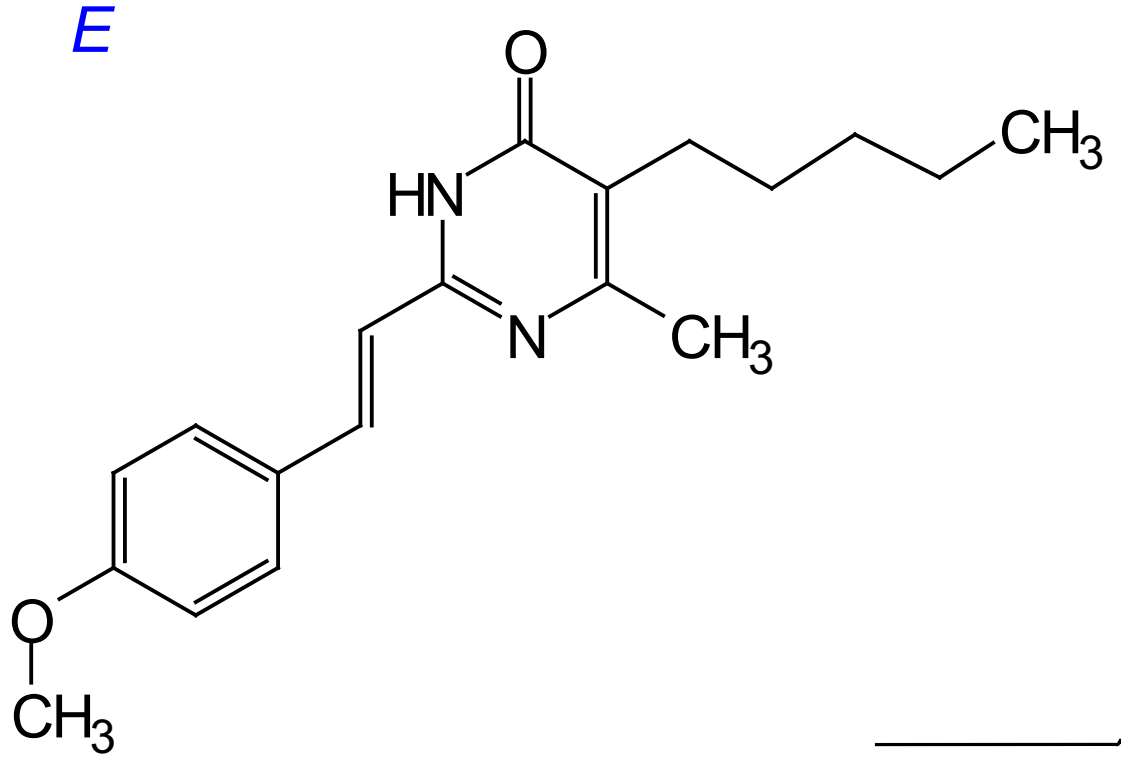

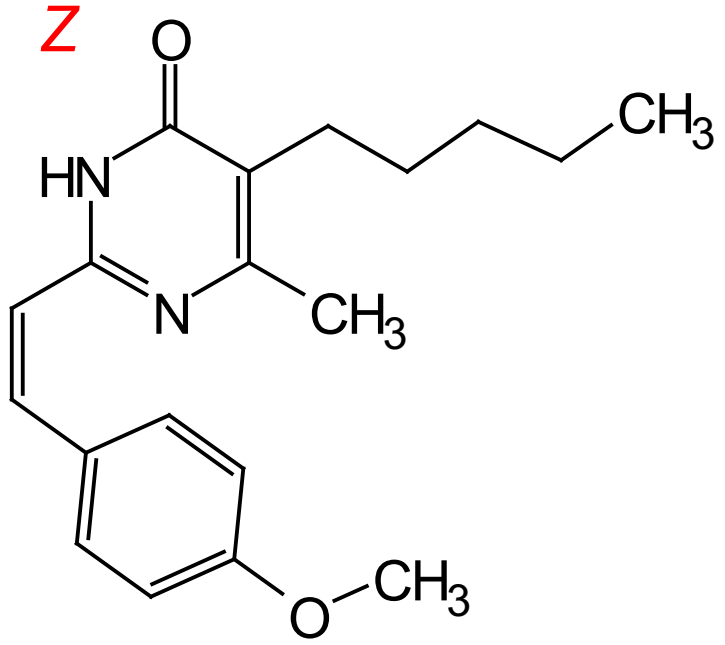

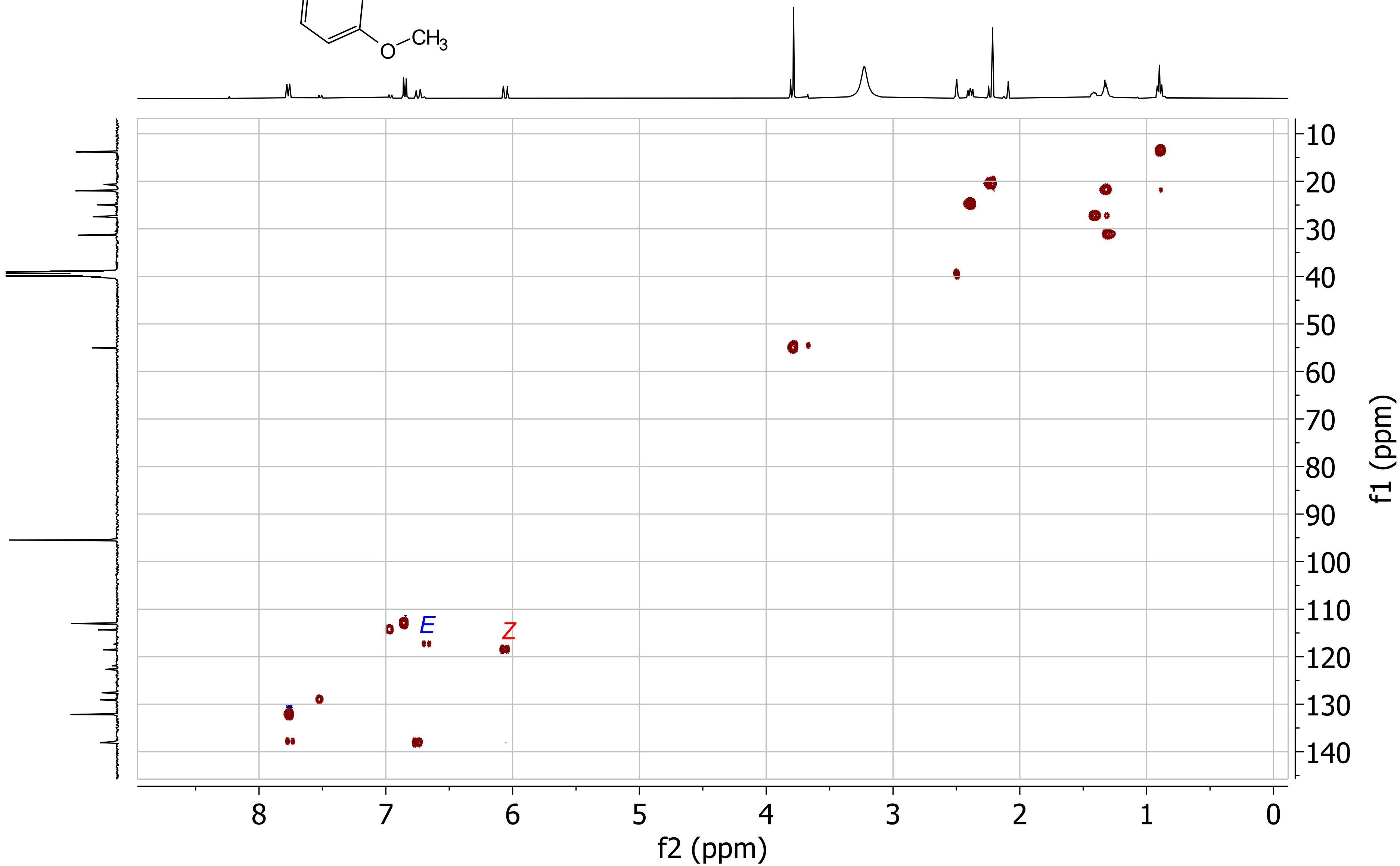

NOESY spectrum of compound 5a, irr. 10h at 420 nm in DMSO-d_6/CCl_4 1/1, 79% *Z*-isomer

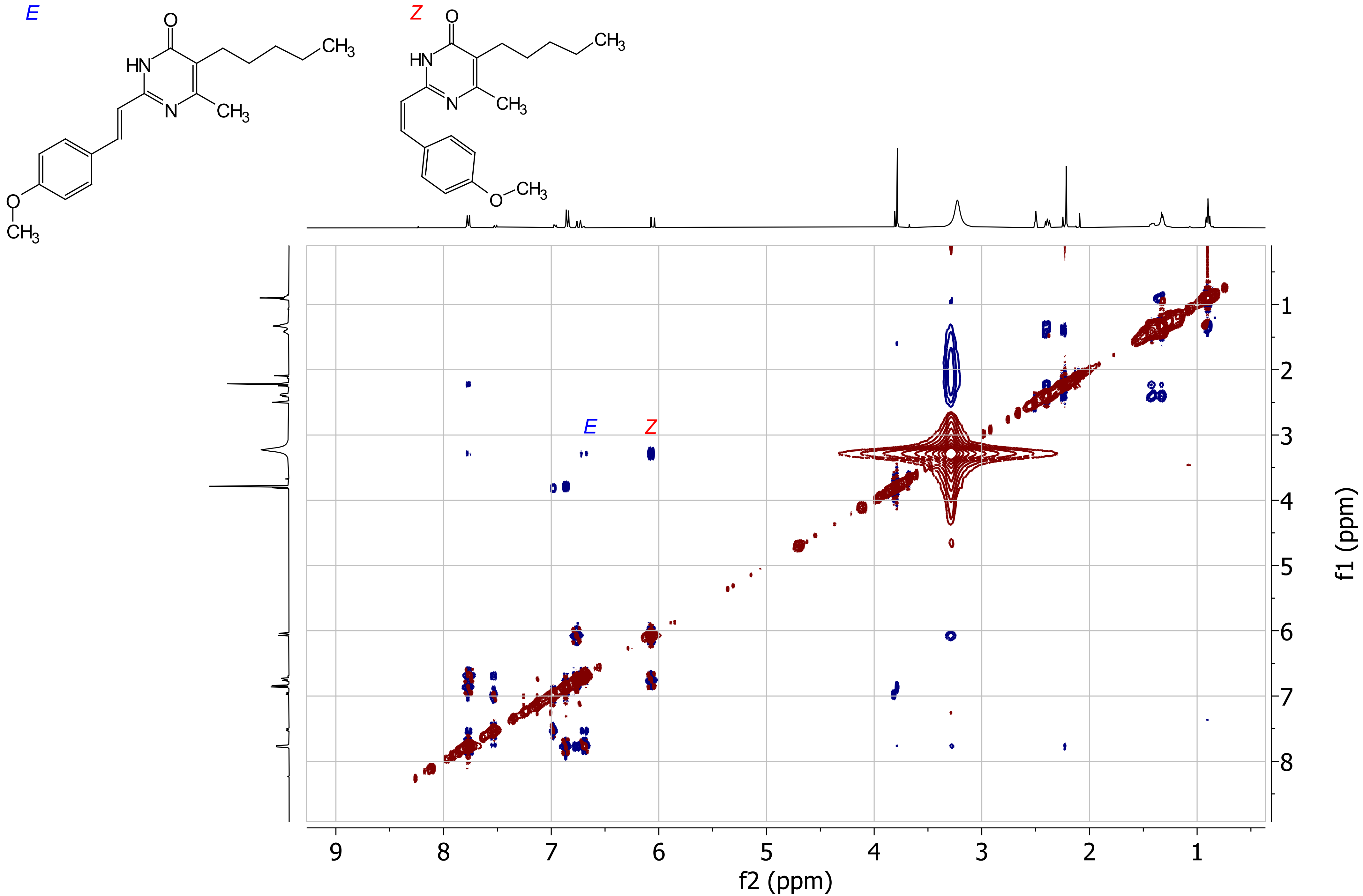

^{1}H NMR spectrum of compound 5b, irr. 1h at 420 nm in DMSO-d_6/CCl_4 1/3, 58% *Z*-isomer and 40% *D* monomer-equivalent cyclodimerized material

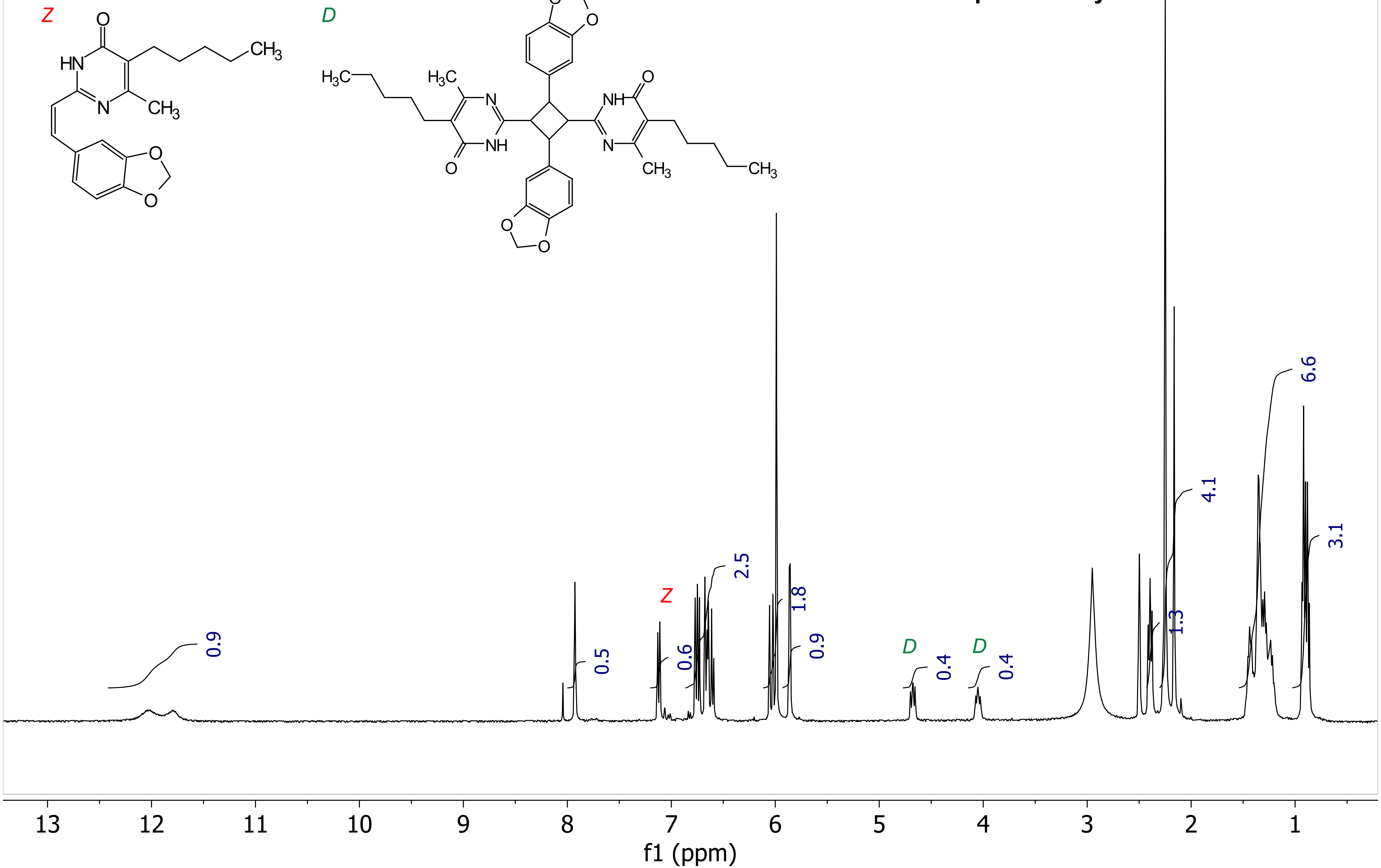

^{1}H NMR spectrum of compound 5c, irr. 2h at 405 nm in DMSO-d$_6$/CCl$_4$ 1/1, 30% *Z*-isomer

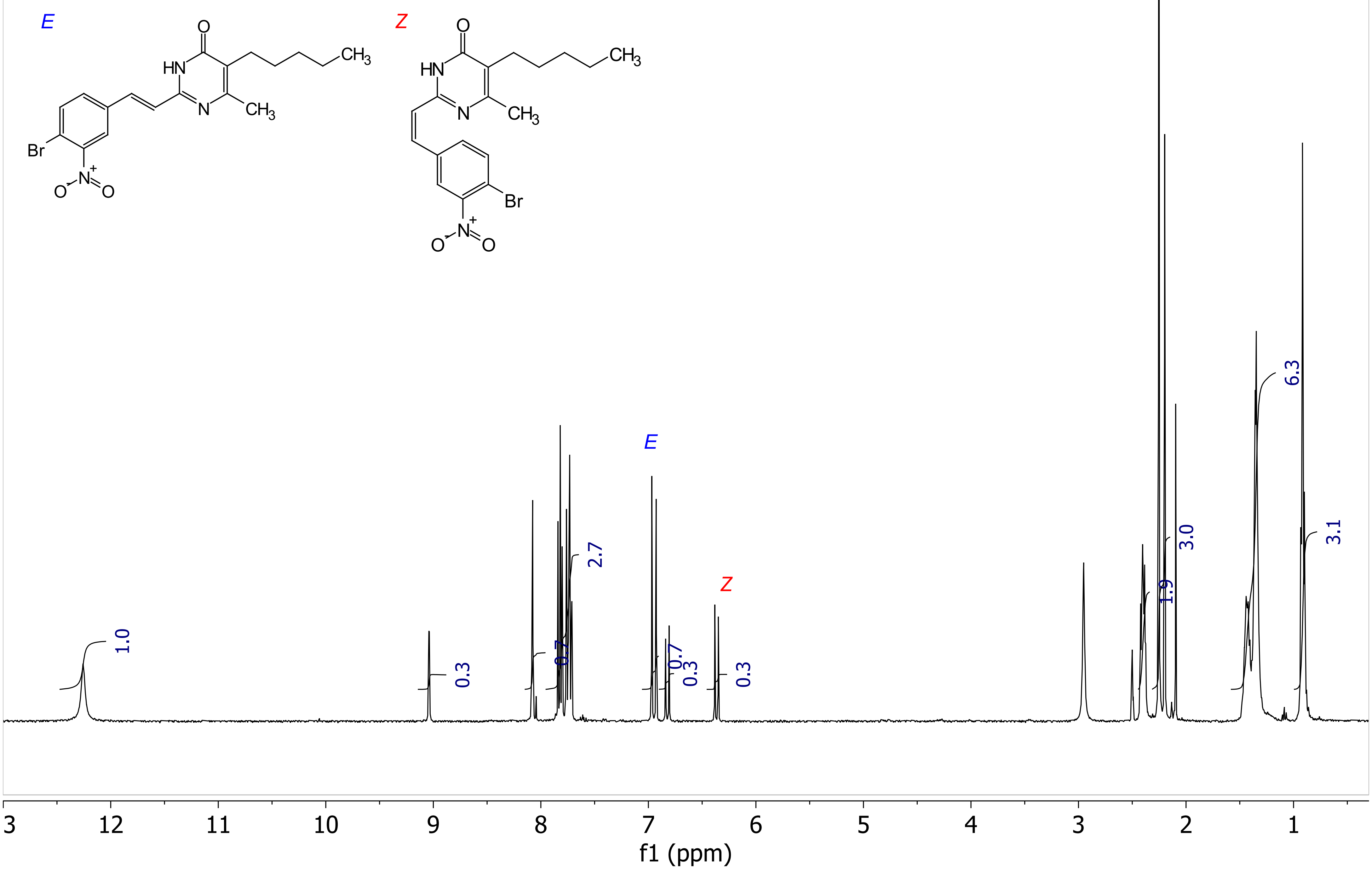

^{1}H NMR spectrum of compound 6a, irr. 5h at 405 nm in DMSO-d_6/CCl_4 1/3, 76% *Z*-isomer

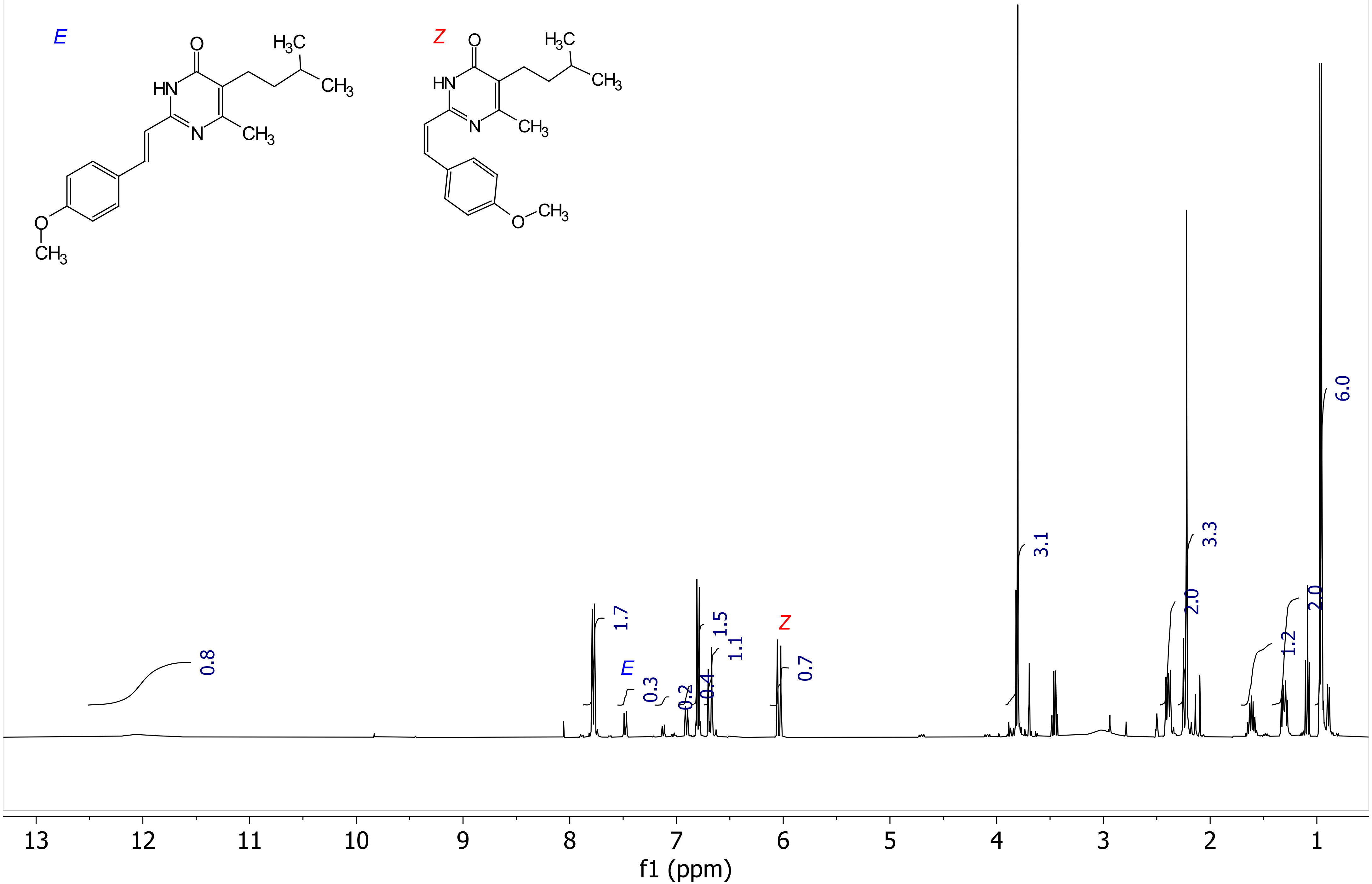

^{1}H NMR spectrum of compound 6b, irr. 4h at 420 nm in DMSO-d$_6$, 79% D monomer-equivalent cyclodimerized material

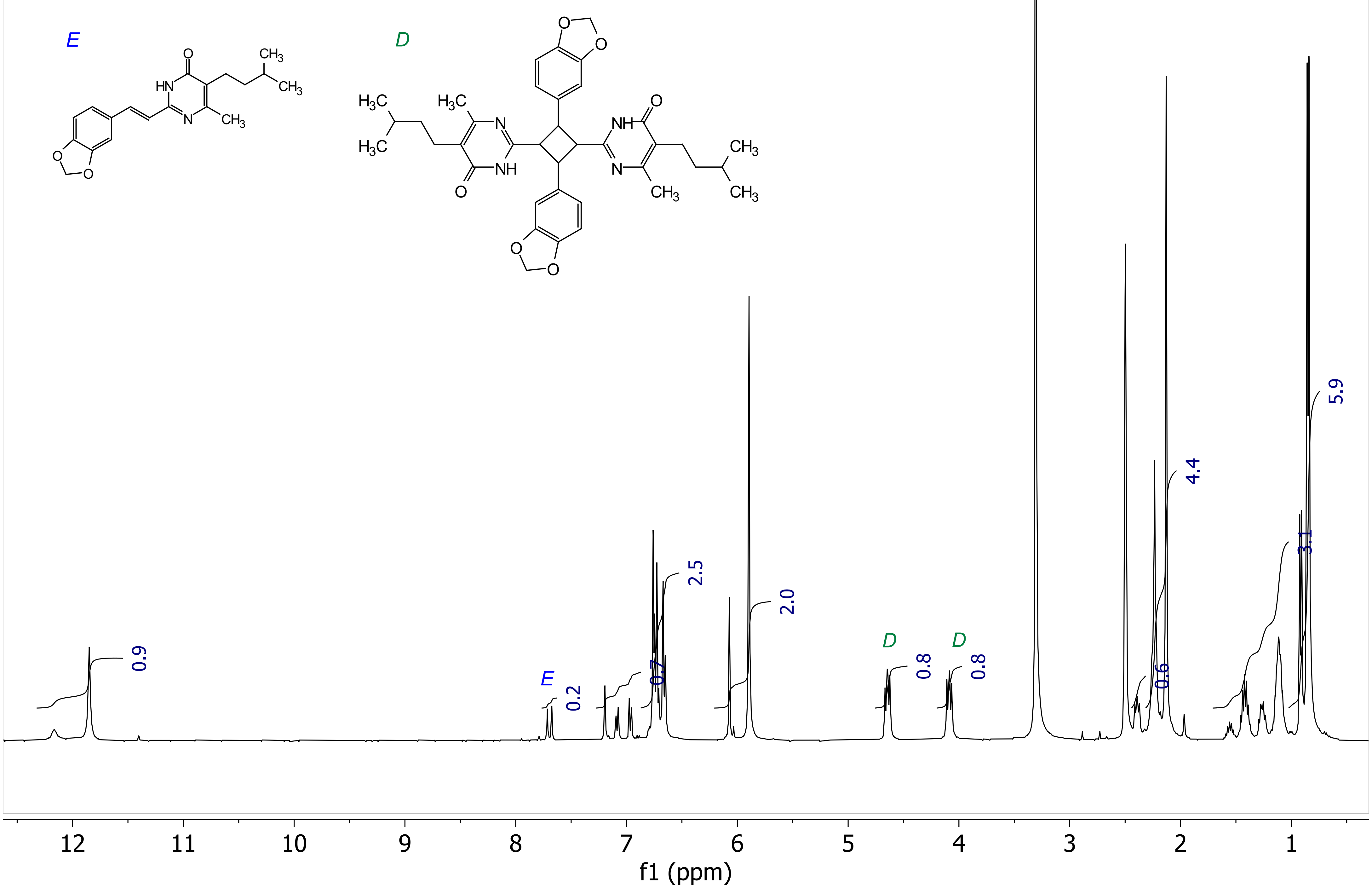

^{1}H NMR spectrum of compound 6b, irr. 1h at 420 nm in DMSO-d_6/CCl4 1/1, 41% *Z*-isomer

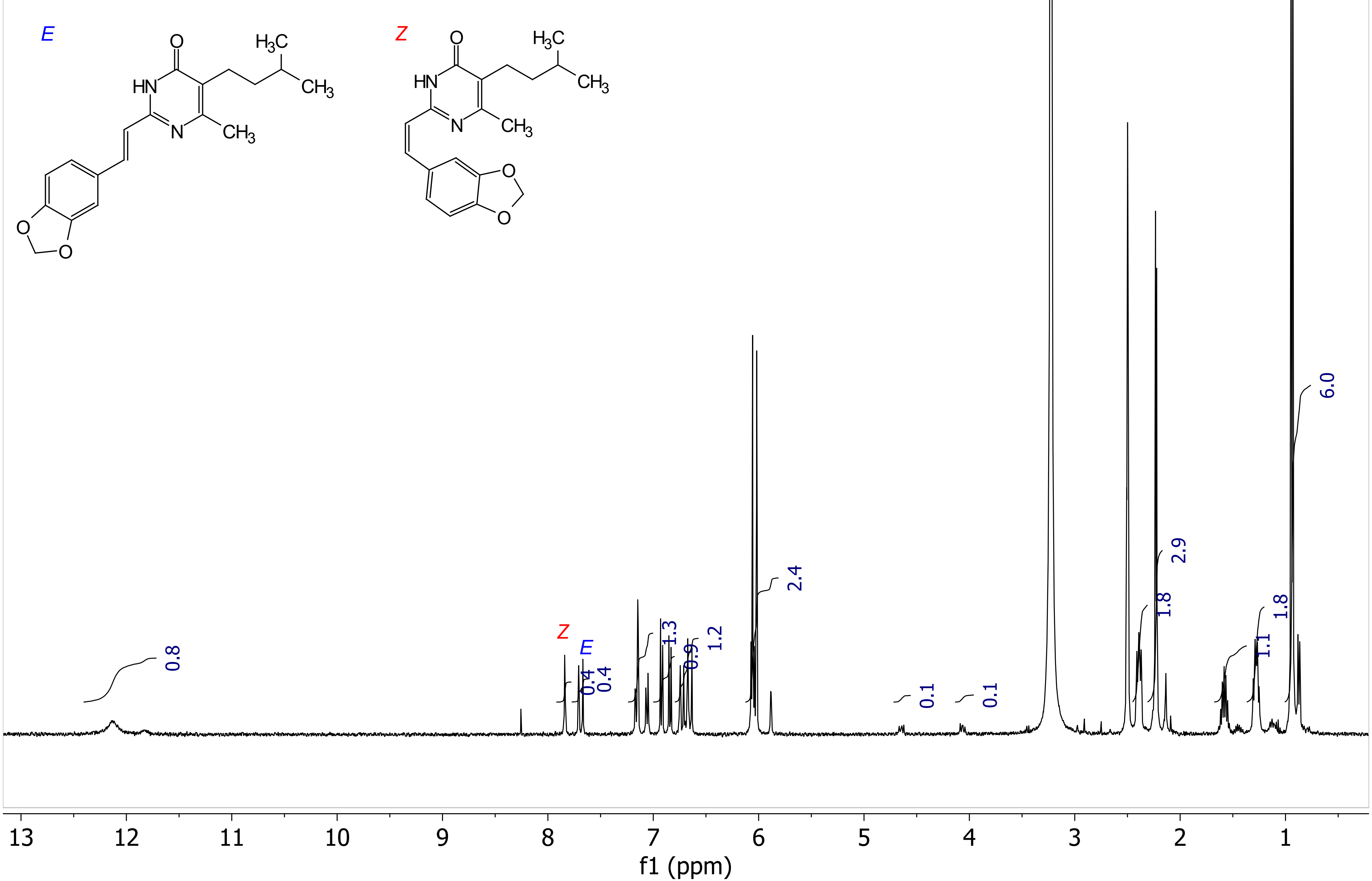

^{1}H NMR spectrum of compound 6c, irr. 3h at 405 nm in DMSO-d_6/CCl4 1/1, 50% *Z*-isomer

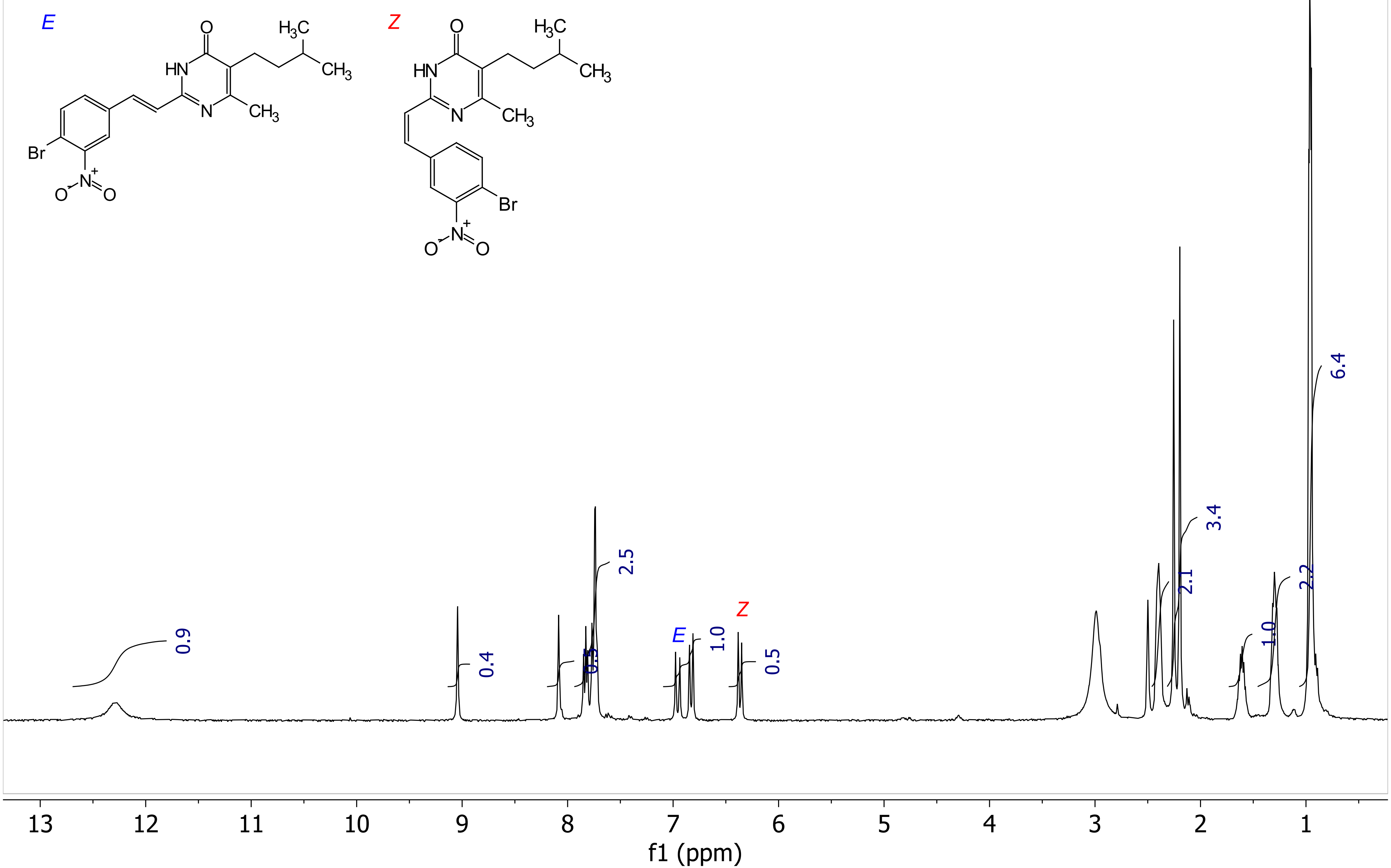

11.21.2025

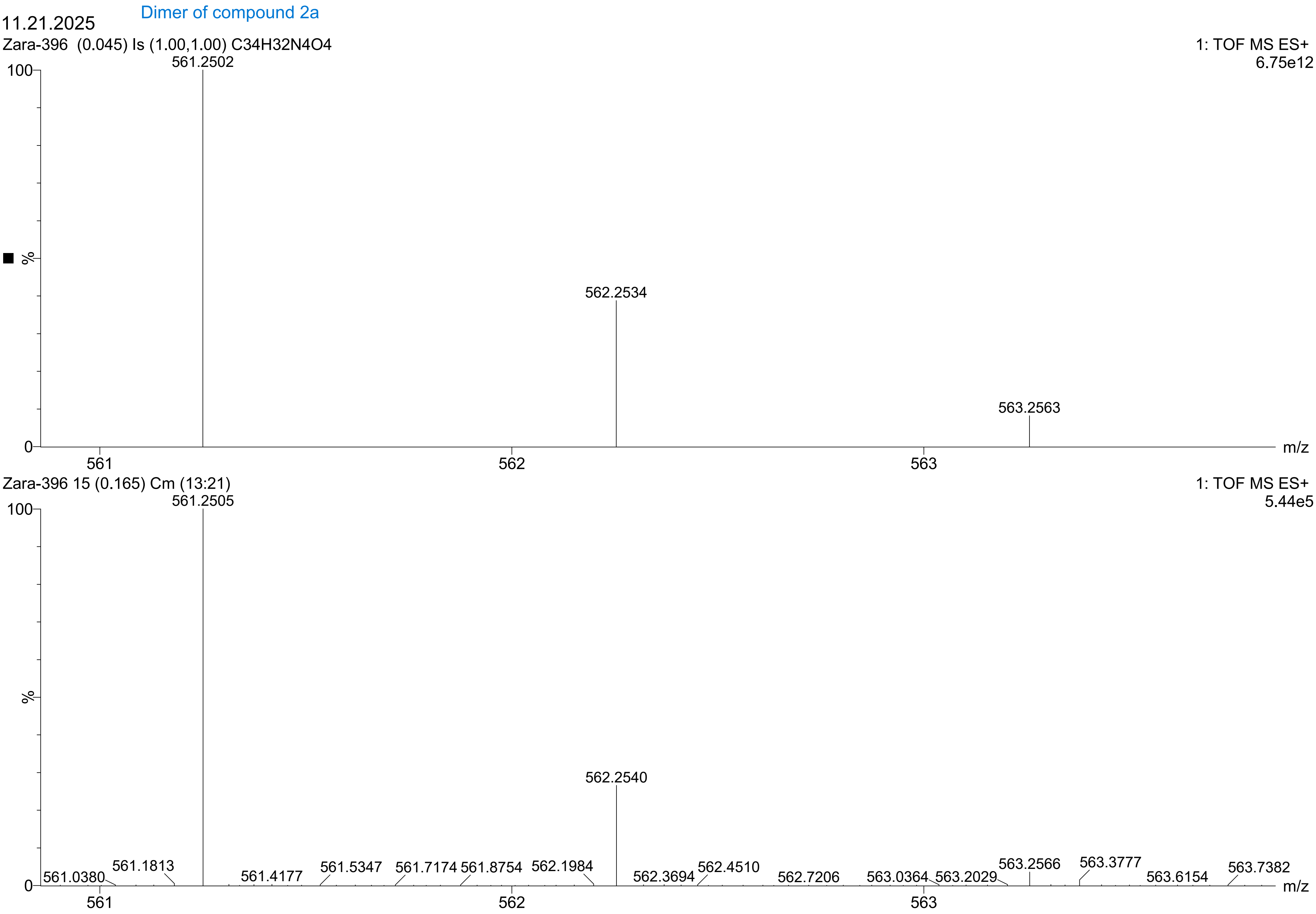

Dimer of compound 2a
Zara-396 (0.045) Is (1.00,1.00) C34H32N4O4
1: TOF MS ES+
6.75e12
561.2502
562.2534
563.2563
100
%
0
561
562
563
m/z
Zara-396 15 (0.165) Cm (13:21)
1: TOF MS ES+
5.44e5
561.2505
562.2540
561.0380
561.1813
561.4177
561.5347
561.7174
561.8754
562.1984
562.3694
562.4510
562.7206
563.0364
563.2029
563.2566
563.3777
563.6154
563.7382

11.21.2025 Dimer of compound 3b

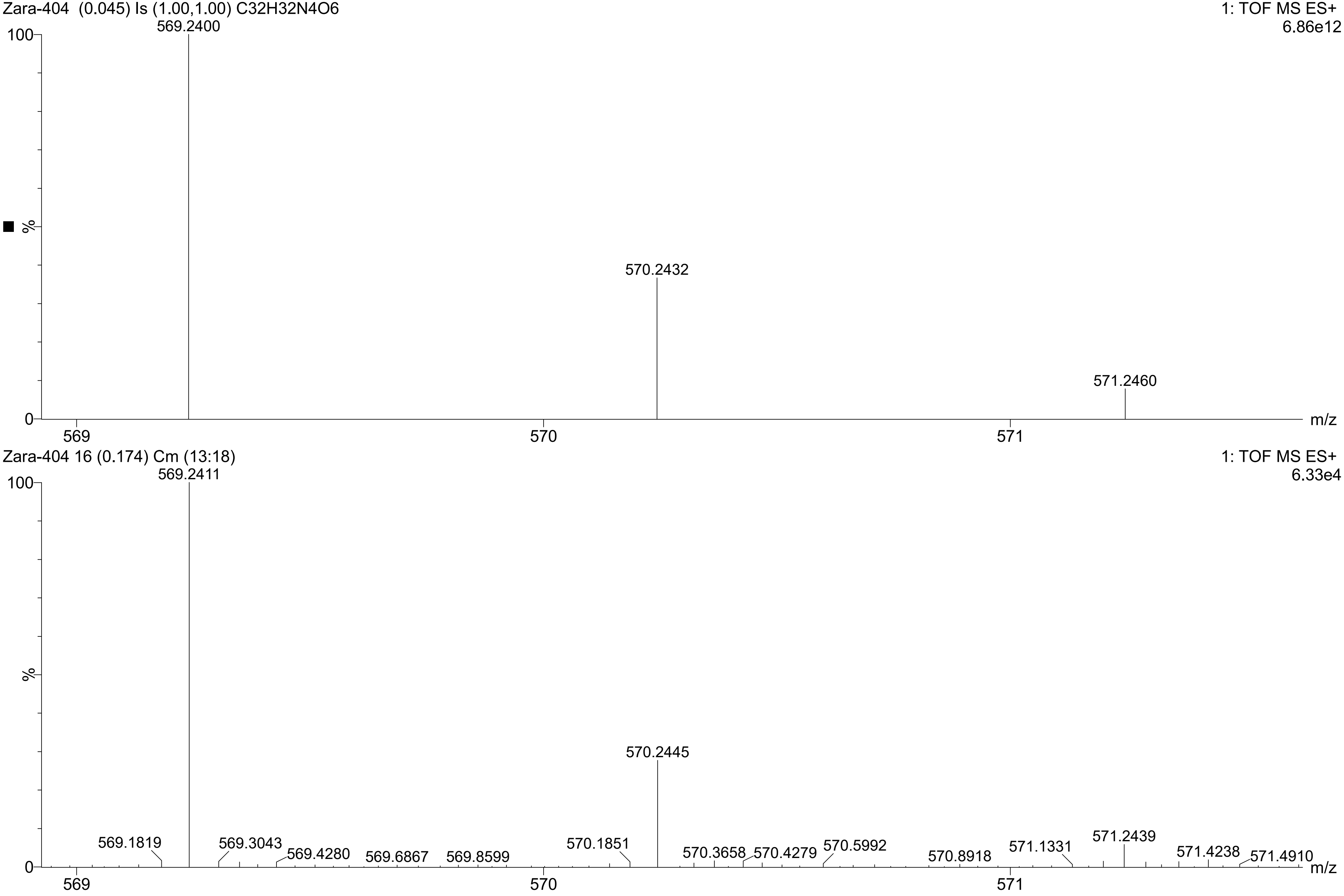

7.09.2025

Compound 4b after irradiation

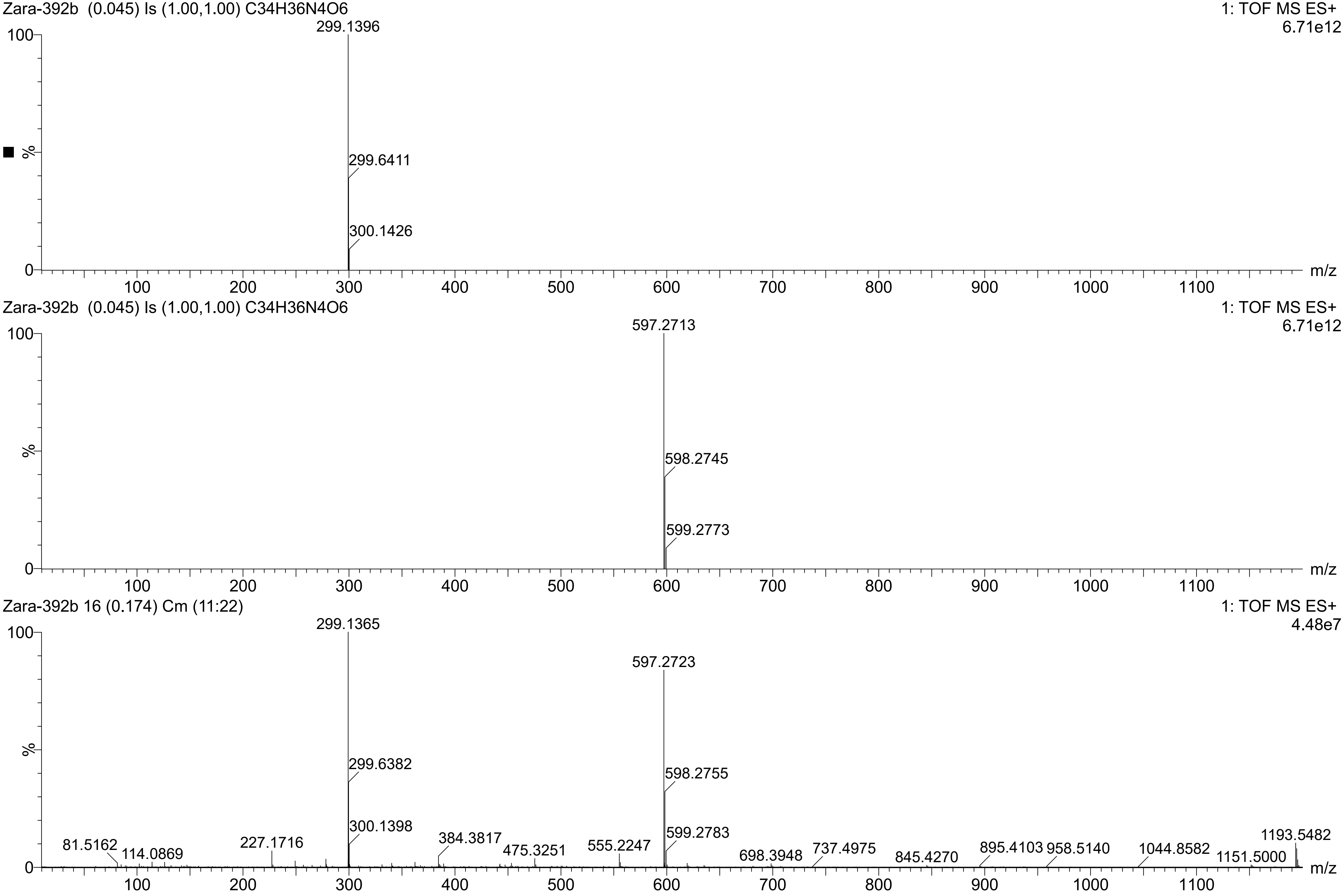

7.09.2025

Dimer of Compound 4b

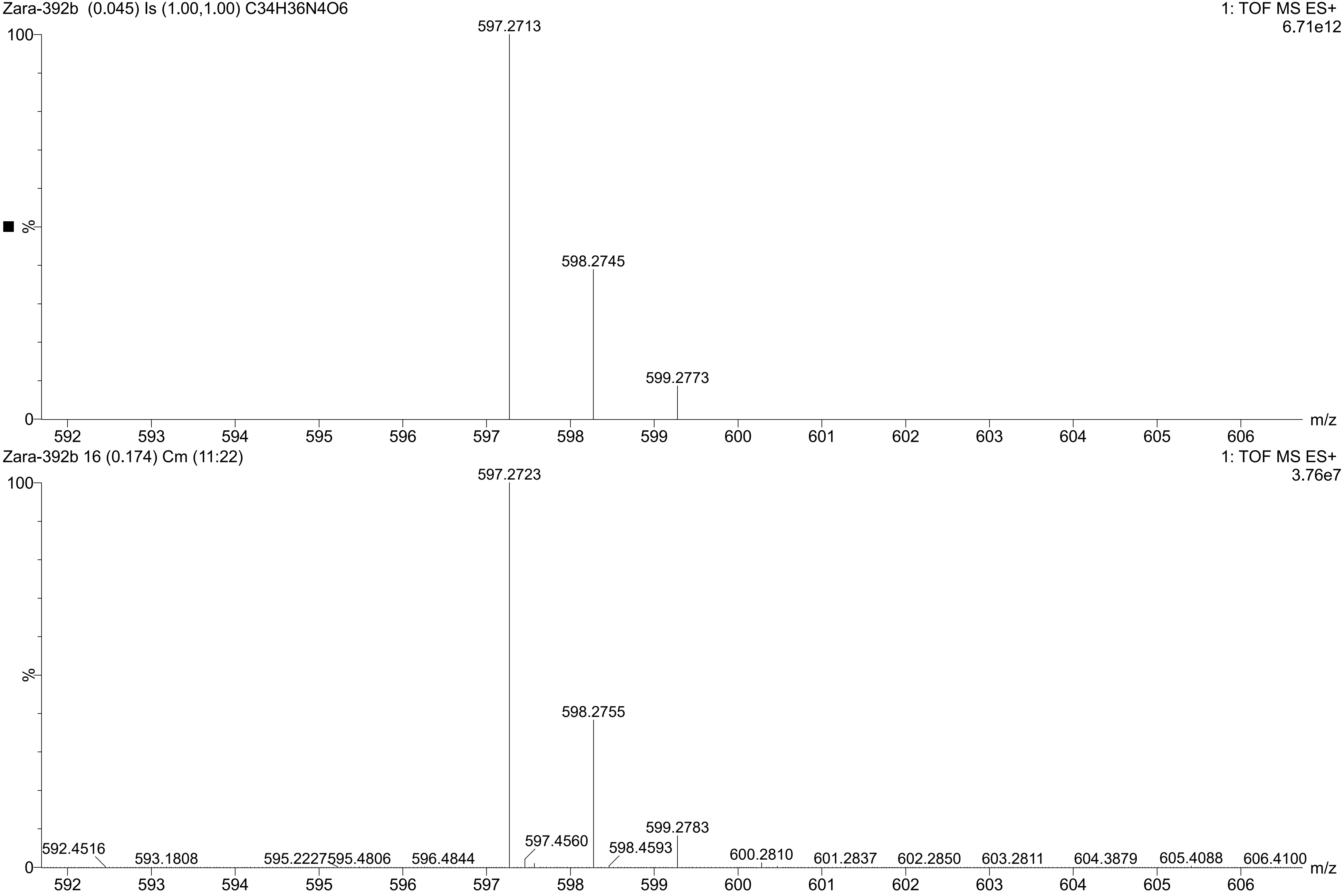

11.21.2025 Dimer of compound 6b

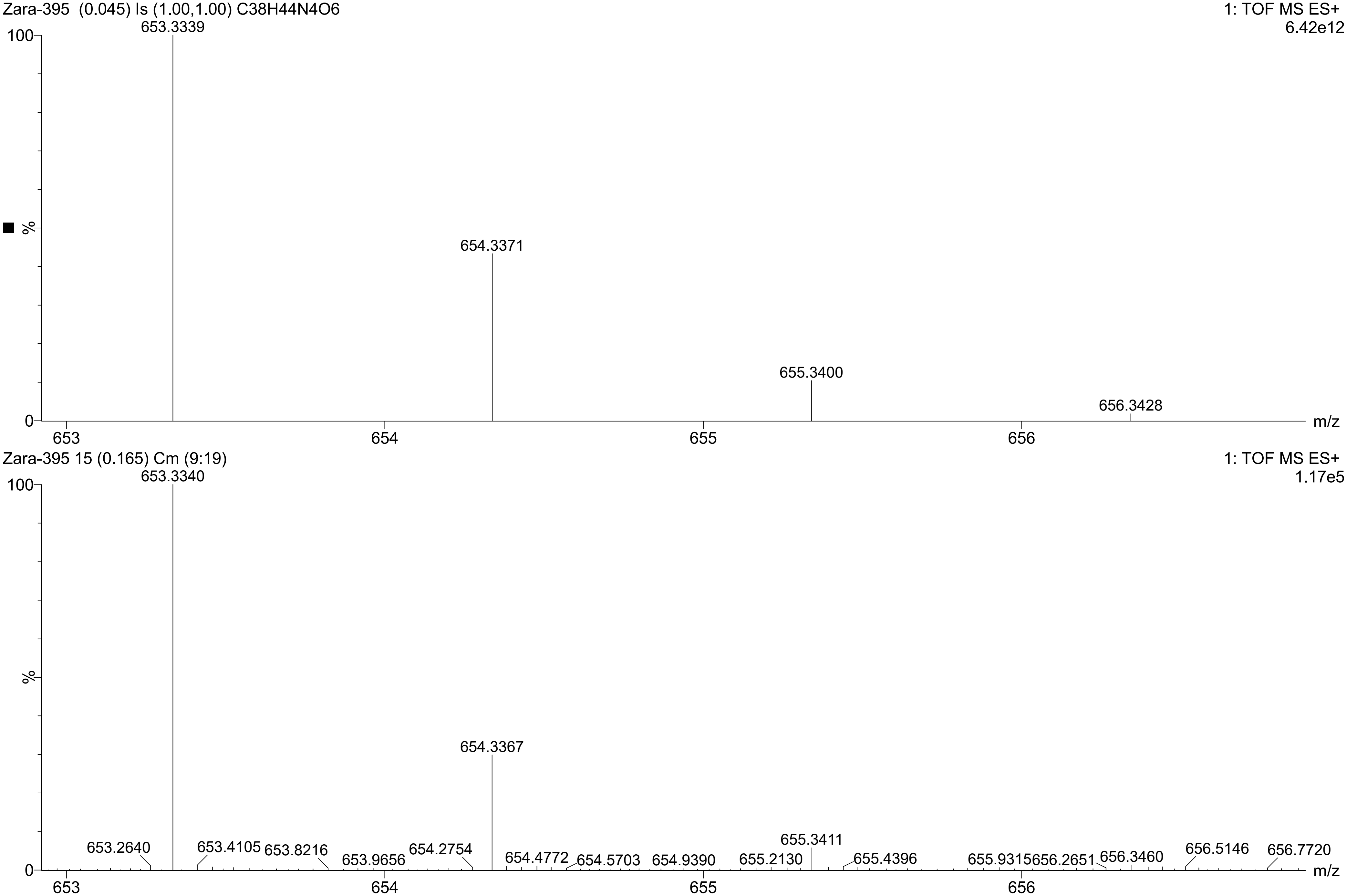

REFERENCES

1 N. M. O'Boyle, M. Banck, C. A. James, C. Morley, T. Vandermeersch and G. R. Hutchison, Open Babel: An Open chemical toolbox, *J. Cheminform.*, DOI:10.1186/1758-2946-3-33.

2 A. K. Rappé, C. J. Casewit, K. S. Colwell, W. A. Goddard and W. M. Skiff, UFF, a Full Periodic Table Force Field for Molecular Mechanics and Molecular Dynamics Simulations, *J. Am. Chem. Soc.*, DOI:10.1021/ja00051a040.

3 P. Pracht, S. Grimme, C. Bannwarth, F. Bohle, S. Ehlert, G. Feldmann, J. Gorges, M. Müller, T. Neudecker, C. Plett, S. Spicher, P. Steinbach, P. A. Wesołowski and F. Zeller, CREST—A program for the exploration of low-energy molecular chemical space, *Journal of Chemical Physics*, DOI:10.1063/5.0197592.

4 C. Bannwarth, S. Ehlert and S. Grimme, GFN2-xTB - An Accurate and Broadly Parametrized Self-Consistent Tight-Binding Quantum Chemical Method with Multipole Electrostatics and Density-Dependent Dispersion Contributions, *J. Chem. Theory Comput.*, DOI:10.1021/acs.jctc.8b01176.

5 S. Ehlert, M. Stahn, S. Spicher and S. Grimme, Robust and efficient implicit solvation model for fast semiempirical methods, *J. Chem. Theory Comput.*, DOI:10.1021/acs.jctc.1c00471.

6 C. Bannwarth, E. Caldeweyher, S. Ehlert, A. Hansen, P. Pracht, J. Seibert, S. Spicher and S. Grimme, Extended tight-binding quantum chemistry methods, 2021, DOI: 10.1002/wcms.1493.

7 S. Grimme and C. Bannwarth, Ultra-fast computation of electronic spectra for large systems by tight-binding based simplified Tamm-Dancoff approximation (sTDA-xTB), *Journal of Chemical Physics*, DOI:10.1063/1.4959605.

8 F. Neese, The ORCA program system, *Wiley Interdiscip. Rev. Comput. Mol. Sci.*, DOI:10.1002/wcms.81.

9 Duarte Group, otherm, University of Oxford, Oxford, UK, 2023, https://github.com/duartegroup/otherm.

10 The pandas development team, Pandas, *Zenodo*, 2020, https://doi.org/10.5281/zenodo.3509134

11 C. R. Harris, K. J. Millman, S. J. van der Walt, R. Gommers, P. Virtanen, D. Cournapeau, E. Wieser, J. Taylor, S. Berg, N. J. Smith, R. Kern, M. Picus, S. Hoyer, M. H. van Kerkwijk, M. Brett, A. Haldane, J. F. del Río, M. Wiebe, P. Peterson, P. Gérard-Marchant, K. Sheppard, T. Reddy, W. Weckesser, H. Abbasi, C. Gohlke and T. E. Oliphant, Array programming with NumPy, 2020, preprint, DOI: 10.1038/s41586-020-2649-2.

12 S. Seabold and J. Perktold, in *Proceedings of the 9th Python in Science Conference*, 2010.

13 Plotly Technologies Inc., Collaborative data science, *Plotly Technologies Inc*, 2015, https://plot.ly

14 F. Pedregosa, G. Varoquaux, A. Gramfort, V. Michel, B. Thirion, O. Grisel, M. Blondel, P. Prettenhofer, R. Weiss, V. Dubourg, J. Vanderplas, A. Passos, D. Cournapeau, M. Brucher, M. Perrot and É. Duchesnay, Scikit-learn: Machine learning in Python, *Journal of Machine Learning Research*.

15 G. Gurzadyan and H. Görner, Picosecond transient absorption spectroscopy of trans-4-R-4′-nitrostilbenes with R: OMe, NH2 and NMe2, *Chem. Phys. Lett.*, DOI:10.1016/S0009-2614(00)00097-X.

16 J. Saltiel, A. Marinari, D. W. L. Chang, J. C. Mitchener and E. D. Megarity, Trans-cis photoisomerization of the stilbenes and a reexamination of the positional dependence of the heavy-atom effect, *J. Am. Chem. Soc.*, DOI:10.1021/ja00505a027.

17 C. Hansch, A. Leo and R. W. Taft, A Survey of Hammett Substituent Constants and Resonance and Field Parameters, *Chem. Rev.*, DOI:10.1021/cr00002a004.
